# New Pyrimidine and Pyridine Derivatives as Multitarget Cholinesterase Inhibitors: Design, Synthesis, and *In Vitro* and *In Cellulo* Evaluation

**DOI:** 10.1021/acschemneuro.1c00485

**Published:** 2021-10-15

**Authors:** Martina Bortolami, Fabiana Pandolfi, Valeria Tudino, Antonella Messore, Valentina Noemi Madia, Daniela De Vita, Roberto Di Santo, Roberta Costi, Isabella Romeo, Stefano Alcaro, Marisa Colone, Annarita Stringaro, Alba Espargaró, Raimon Sabatè, Luigi Scipione

**Affiliations:** † Department of Scienze di Base e Applicate per l’Ingegneria, Sapienza University of Rome, via Castro Laurenziano 7, I-00161 Rome, Italy; ‡ Department of Chimica e Tecnologia del Farmaco, Sapienza University of Rome, Piazzale Aldo Moro 5, 00185 Rome, Italy; § Department of Environmental Biology, Sapienza University of Rome, Piazzale Aldo Moro 5, 00185 Rome, Italy; ∥ Istituto Pasteur, Fondazione Cenci Bolognetti, Department of Chemistry and Technology of Drug, Sapienza University of Rome, Piazzale Aldo Moro 5, 00185 Rome, Italy; ⊥ Net4Science s.r.l., Campus universitario ″S. Venuta″, Viale Europa, 88100 Catanzaro, Italy; # Dipartimento di Scienze della Salute, Università ″Magna Græcia″ di Catanzaro, Viale Europa, 88100 Catanzaro, Italy; ∇ National Center for Drug Research and Evaluation, Istituto Superiore di Sanità, Viale Regina Elena, 00161 Rome, Italy; ○ Department of Pharmacy and Pharmaceutical Technology and Physical-Chemistry, Faculty of Pharmacy and Food Sciences, University of Barcelona, Avda. Joan XXIII, 27-31 Barcelona, Catalonia, Spain

**Keywords:** acetylcholinesterase inhibitors, butyrylcholinesterase inhibitors, multifunctional compounds, amyloid aggregation, tau aggregation, metal chelation, antioxidant

## Abstract

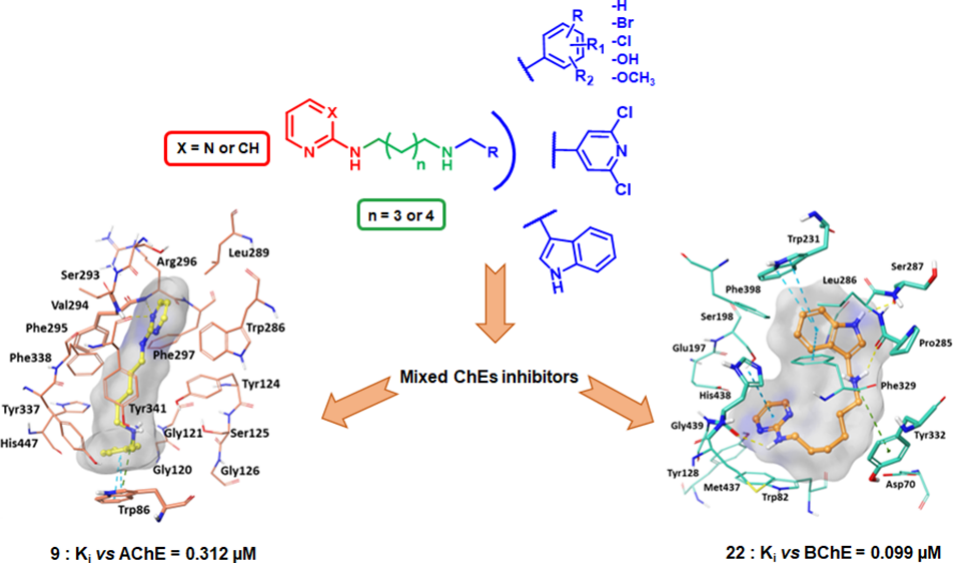

A new series of pyrimidine and pyridine diamines was designed as dual binding site inhibitors of cholinesterases (ChEs), characterized by two small aromatic moieties separated by a diaminoalkyl flexible linker. Many compounds are mixed or uncompetitive acetylcholinesterase (AChE) and/or butyrylcholinesterase (BChE) nanomolar inhibitors, with compound **9** being the most active on *Electrophorus electricus* AChE (*Ee*AChE) (*K*_i_ = 0.312 μM) and compound **22** on equine BChE (*eq*BChE) (*K*_i_ = 0.099 μM). Molecular docking and molecular dynamic studies confirmed the interaction mode of our compounds with the enzymatic active site. UV–vis spectroscopic studies showed that these compounds can form complexes with Cu^2+^ and Fe^3+^ and that compounds **18**, **20**, and **30** have antioxidant properties. Interestingly, some compounds were also able to reduce Aβ_42_ and tau aggregation, with compound **28** being the most potent (22.3 and 17.0% inhibition at 100 μM on Aβ_42_ and tau, respectively). Moreover, the most active compounds showed low cytotoxicity on a human brain cell line and they were predicted as BBB-permeable.

## Introduction

Alzheimer’s disease (AD) is one of the most serious and prevalent neurodegenerative diseases; it accounts for 50–75% of dementia cases in humans, currently affecting 44 million people worldwide,^1^ and causes huge social economic losses.^2^ In 2015, the World Alzheimer Report estimated over 9.9 million new cases of dementia each year (approximately one new case every 3 s), which allows one to predict a population of 70 million by 2030 that could rise to 130 million by 2050.^3^ AD begins with short-term memory loss followed by a progressive decline in memory and cognitive ability associated with severe behavioral abnormalities, such as irritability, anxiety, and depression, and the outcome is always fatal.

Although the precise pathogenetic mechanisms of AD remain to be elucidated, several factors appear to play key roles in the onset and progression of the AD, and they comprise the following: the cholinergic deficit, with reduced levels of acetylcholine (ACh) and many cholinergic markers; the β-amyloid peptide (Aβ) deposition; the hyperphosphorylation and the deposition of the tau protein; the dysregulation of energy metabolism and the increase in oxidative stress; the dyshomeostasis of biometals; and neuroinflammation.^4^ The cholinergic hypothesis, the first approach developed to describe the pathophysiology of AD, is based on three main evidences observed in AD patients: (i) a severe neurodegeneration of the nucleus basalis of Meynert, the main source of cortical cholinergic innervation; (ii) a severe depletion of the presynaptic cholinergic markers, such as choline acetyl transferase (ChAT); and (iii) the cholinergic antagonist drugs induce memory impairment and cognitive deficits that are alleviated by cholinergic agonists.^5−8^

These evidences have led to the therapeutic use in AD of cholinesterase inhibitors (ChEIs), able to restore cholinergic tone by blocking the cholinesterase (ChE) activity and consequently increasing the availability of ACh at the synaptic cleft.

To date, the ChEIs remain the primary therapy for AD, with donepezil, galantamine, and rivastigmine, currently approved by the Food and Drug Administration (FDA), along with the NMDA receptor antagonist memantine.^9^ Unfortunately, these drugs have shown limited clinical results, with short-lasting positive effects and the lack of ability to stop the progression of the disease. The absence of decisive and long-term effects stimulates researchers to develop new therapeutic strategies with the objective of restoring cerebral cholinergic activity, as the degeneration of the cortical cholinergic projection represents a key irreversible event in the progression of AD.

At the neuronal level, there are two types of ChEs: acetylcholinesterase (AChE) and butyrylcholinesterase (BChE). AChE activity is dominant in the healthy brain (80%), while BChE exerts a supportive role. However, with the progression of the disease, brain levels of AChE decline to approximately 60%, while those of BChE increase to 120% of normal levels, thus suggesting for the latter a major role in the severe forms of the disease and the possibility of considering it an adequate therapeutic target of AD.^10,11^

AChE and BChE have 65% amino acid sequence identity; they possess a catalytic active site (CAS), located at the bottom of a 20 Å gorge, constituted by a catalytic triad: Ser203, His447, and Glu202 in human AChE (*h*AChE) and Ser198, His438, and Glu197 in human BChE (*h*BChE). An anionic subsite is also present that is constituted by Trp86 in *h*AChE and Trp82 in *h*BChE, which bind the quaternary ammonium of the ACh moiety by means of cation−π interaction.^12,13^ These enzymes exhibit an allosteric modulation site, the peripheral anionic site (PAS), located at the entrance of the active site gorge, which in *h*AChE consists of five residues, Tyr72, Asp74, Tyr124, Trp286, and Tyr341, while in *h*BChE is constituted by Tyr332 and Asp70.^14,15^ Besides its role in the modulation of the catalytic activity, PAS has been reported to be involved in the proaggregating action of AChE toward the Aβ peptide, another important actor in the onset and progression of AD.^16,17^

The growing scientific evidences highlighting the close correlation between the severe cholinergic depletion and the other pathophysiological events observed in AD, particularly with the abnormal Aβ and tau cascade, give a new interest to the development of new ChEIs for the treatment of AD and, in particular, toward multitarget compounds able to act simultaneously at more levels on the different mechanisms involved in the pathogenesis of AD.^18^ Many reviews available in the scientific literature summarize the main research lines followed in the development of multitarget compounds for the therapy of AD in both the academic and industrial fields. The majority of articles and patents report studies on dual binding site ChEIs that are able to interact with both CAS and PAS of these enzymes and are endowed with the activity on one or more additional targets, such as a second neurotransmitter system (*i.e.*, serotonergic or monoaminergic), or Aβ and tau production and deposition, the reduction of oxidative stress, or biometal dyshomeostasis.^19−21^

In our previous studies, we synthesized and *in vitro* evaluated new series of dual binding site ChEIs, characterized by two small aromatic moieties separated by various functionalized linkers, some of which resulted in nanomolar mixed or uncompetitive inhibitors of both AChE and BChE, able to bind CAS and PAS, able to reduce Aβ aggregation, or endowed with metal chelating properties.^22,23^

**Figure 1:**
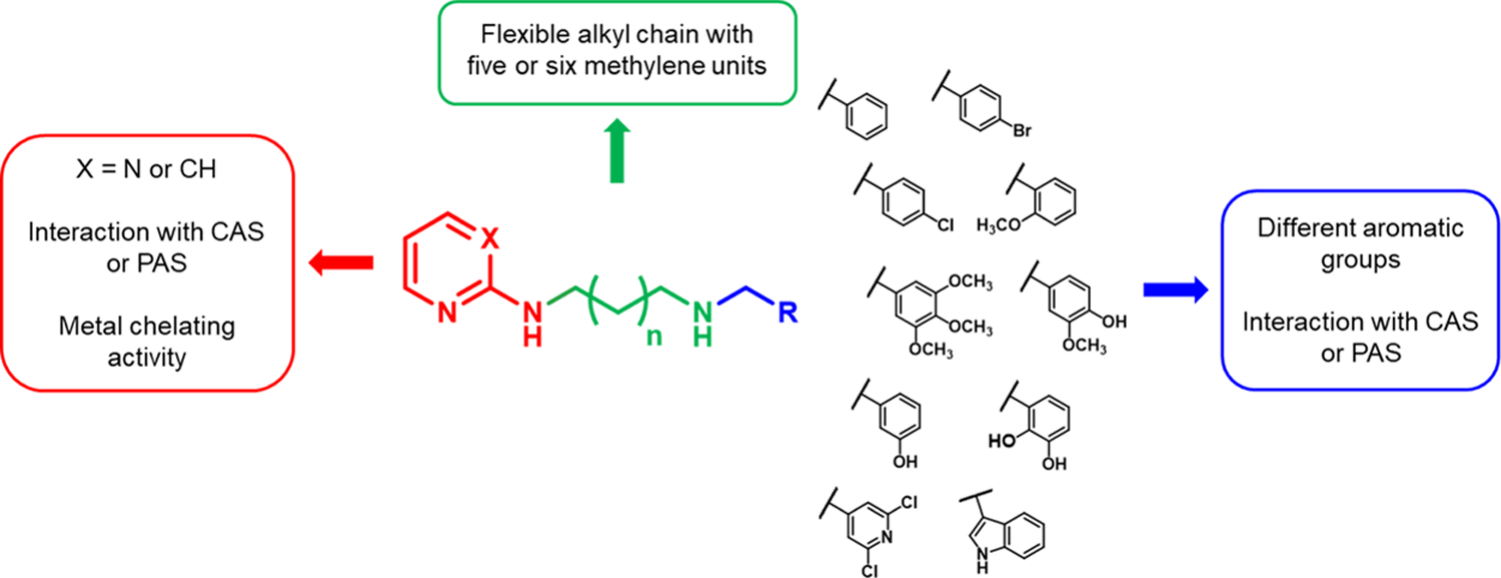
Rational design of the 2-amino-pyrimidine or 2-amino-pyridine derivatives.

Based on these considerations and as a development of previous works, we decided to study a new series of pyrimidine and pyridine diamine derivatives and evaluate them both *in vitro* and *in cellulo* as potential ChEIs endowed with chelating and antioxidant activities as well as direct anti-amyloid aggregation properties, with the aim to develop new multifunctional compounds for AD. These molecules were designed as dual binding site inhibitors, based on the structure of the ChE enzymatic pocket, inserting two small aromatic groups separated by an aliphatic linker (Figure 1). The aromatic moieties are potentially able to interact with the aromatic amino acids located adjacent to the CAS and in the PAS of the enzymes by means of π–π interactions, while the aliphatic linker covers the distance between CAS and PAS and gives flexibility to the compounds. Due to the difficulty to predict the conformational behavior of linear alkyl chains, different length linkers were used, in particular with five or six methylene units. Within the chain, a protonable amine group, both acceptor and donor of hydrogen bond and able to establish cationic−π interactions with the aromatic residues of the enzymes, was inserted to promote interactions with the amino acids of the enzyme gorge. A 2-amino-pyrimidinic or 2-amino-pyridinic moiety was introduced as one of the two aromatic groups since the presence of the two adjacent nitrogen atoms could confer chelating activity to the compounds. On the other side of the aliphatic chain, different aromatic groups were chosen, with the purpose of acquiring further information about the structural requirements for targeting ChEs. Among these groups, phenolic or catecholic rings were also selected to enhance chelating properties and to confer direct antioxidant activity.

## Results and Discussion

### Chemistry

#### General Procedure for the Synthesis of Pyrimidine Diamine Derivatives **9**–**22**

**Scheme 1:**
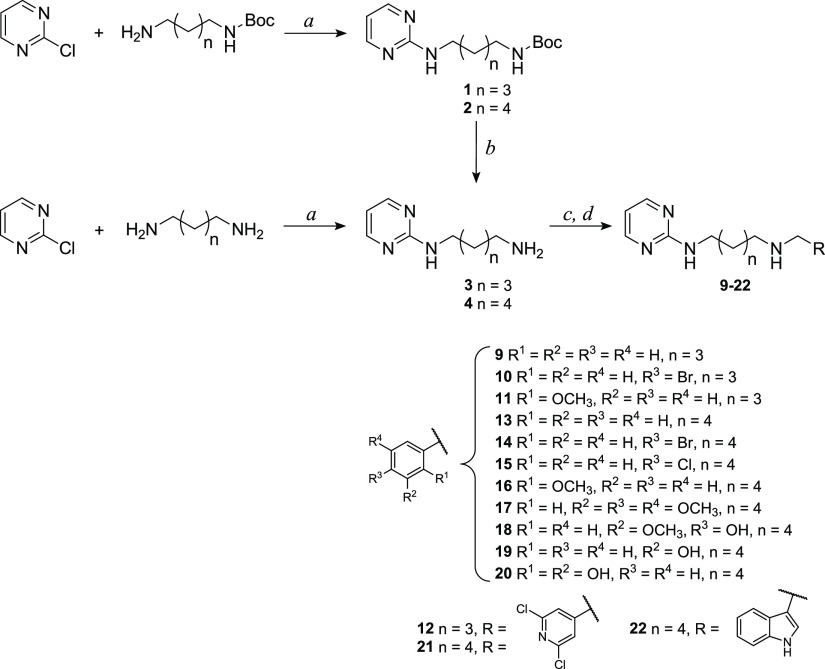
Synthetic Route to Pyrimidine Diamine **9**–**22** Derivatives Reagents and conditions: (*a*) TEA (1 equiv), MeOH, reflux, 20 h; (*b*) TFA (20 equiv), dry CH_2_Cl_2_, room temp, 3.5 h; (*c*) opportune aldehyde (1 equiv), dry CH_2_Cl_2_, dry K_2_CO_3_ (for **9**–**16** and **21**) or activated molecular sieves (for **17**–**20** and **22**), room temp, 12 h; and (*d*) NaBH_4_ (3 equiv), MeOH, room temp, 2 h.

The pyrimidine diamine derivatives **9**–**22** were synthetized following the procedures described in Scheme 1. Initially, the intermediates **3** and **4** were synthesized through two reaction steps (first method, GP-A and procedure 1 or 2 of GP-B in the Experimental Section); subsequently, intermediate **4** was also obtained directly through a single reaction (second method, procedure 3 of GP-B in the Experimental Section). Following the first synthetic method, equimolar amounts of 2-chloropyrimidine, *N*-Boc-1,5-diaminopentane, or *N*-Boc-1,6-diaminohexane and triethylamine (TEA) were reacted in methanol at reflux overnight to give compounds **1** or **2**, which were purified by column chromatography on a silica gel. Then, intermediates **1** and **2** were deprotected from Boc by treatment with a high excess of trifluoroacetic acid (TFA) in anhydrous dichloromethane to obtain compounds **3** and **4**, which were used either as trifluoroacetic salts or as free amines. According to the second synthetic method, intermediate **4** was synthetized directly by the reaction between 2-chloropyrimidine and an excess of 1,6-diaminohexane in the presence of TEA in methanol at reflux overnight. The excess of diamine allowed one to obtain mainly the product of interest, in which only one amino group reacted with 2-chloropyrimidine. The reaction was monitored by ESI-MS, and the crude residue was purified by column chromatography on a silica gel. The second method proved to be more advantageous than the first, as through a single step it was possible to synthetize the desired pyrimidine intermediate with a higher yield.

Equimolar amounts of amine intermediates **3** or **4** and the appropriate aldehyde were dissolved in dry dichloromethane in the presence of potassium carbonate or molecular sieves as desiccant agents. The choice of the suitable drying agent was based on the starting reagents: when compound **3** or **4** was used as trifluoroacetic salt, potassium carbonate was utilized both as a desiccant, either as a base to deprotonate the amino groups of the intermediates; when the reaction was carried out using aldehydes containing acidic groups susceptible to react with potassium carbonate (such as indole −NH or phenol −OH), molecular sieves were selected as desiccant agents. These reactions were monitored by IR spectroscopy to confirm the imine formation, with the appearance of a stretching absorption band around 1640 cm^–1^ and the disappearance of the aldehyde band at about 1700 cm^–1^. The obtained imine intermediates were reduced by treatment with sodium borohydride in methanol, and after the reaction, the excess of sodium borohydride was decomposed by the addition of water or 1 M HCl. The addition of hydrochloric acid was necessary to avoid the oxidation of phenolic derivatives at basic pH.

The residues obtained were purified by column chromatography on a silica gel and/or by crystallization. Moreover, the structures of final compounds were confirmed by spectroscopic analysis; as an example, in the ^1^H NMR spectra, the appearance of the methylene singlet between 3.89 and 3.63 ppm was observed. The detailed synthetic procedures and the analytical and spectroscopic data of synthesized compounds are reported in the Experimental Section and agree with the proposed structures.

#### General Procedure for the Synthesis of Pyridine Diamine Derivatives **23**–**33**

**Scheme 2:**
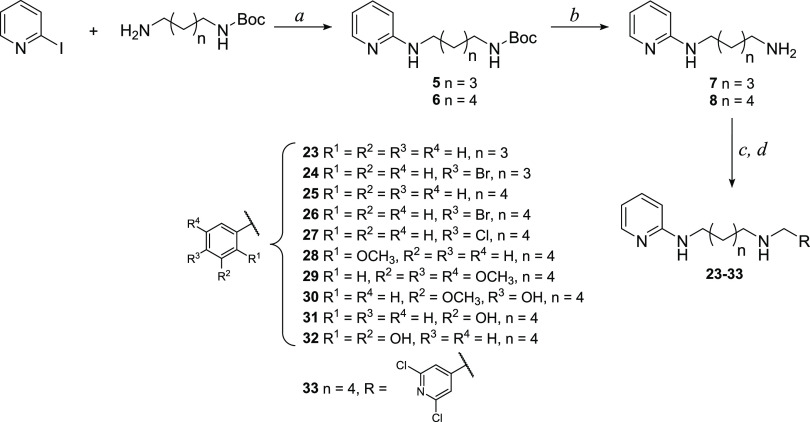
Synthetic Route to Pyridine Diamine **23**–**33** Derivatives Reagents and conditions: (*a*) CuI (0.05 equiv), Cs_2_CO_3_ (2 equiv), 2-isobutyrylcyclohexanone (0.2 equiv), DMF, N_2_, 40 °C, 21 h; (*b*) TFA (20 equiv), dry CH_2_Cl_2_, room temp, 3.5 h; (*c*) opportune aldehyde (1 equiv), dry CH_2_Cl_2_, dry K_2_CO_3_ (for **23**–**28** and **33**) or activated molecular sieves (for **29**–**32**), room temp, 12 h; and (*d*) NaBH_4_ (3 equiv), MeOH, room temp, 2 h.

Pyridine diamine derivatives **23**–**33** were synthetized following the procedures described in Scheme 2. Intermediates **5** and **6** were obtained by Ullmann coupling catalyzed by CuI and 2-isobutyrylcyclohexanone, as described by Shafir and Buchwald.^24^ A Schlenk flask was charged with *N*-Boc-1,5-diaminopentane or *N*-Boc-1,6-diaminohexane and solid reactants (CuI and Cs_2_CO_3_), and then it was evacuated and backfilled with N_2_. Under a counter flow of N_2_, 2-iodopyridine, dimethylformamide (DMF), and finally 2-isobutyrylcyclohexanone were added. The reaction was monitored by ESI-MS, and the best yield was obtained stirring the mixture at 40 °C for 21 h. Purification by column chromatography on a silica gel led to the isolation of the products of interest with quantitative yield. Then, intermediate **5** and **6** were deprotected from Boc by treatment with a high excess of TFA in anhydrous dichloromethane to obtain compounds **7** and **8**, which were used either as trifluoroacetic salts or as free amines. Differently from what has been said for the pyrimidine intermediates, in this case, it was not possible to synthetize compounds **5** and **6** directly through a single reaction between 2-iodopyridine and an excess of the opportune diamine. In fact, in this way, the product in which both amino groups reacted with 2-iodopyridine was mainly obtained.

Then, intermediates **7** and **8** were reacted with the appropriate aldehydes, and the imines obtained were reduced with sodium borohydride, as previously described for the pyrimidine derivatives. The residues obtained were purified by column chromatography on a silica gel or by crystallization. The structures of final compounds were confirmed by spectroscopic analysis; as an example, in the ^1^H NMR spectra, the appearance of the methylene singlet between 3.90 and 3.63 ppm was observed. The detailed synthetic procedures and the analytical and spectroscopic data of synthesized compounds are reported in the Experimental Section and agree with the proposed structures.

### Enzymatic Assays

On the synthesized compounds, enzymatic inhibition studies were carried out toward *Electrophorus electricus* AChE (*Ee*AChE) and equine BChE (*eq*BChE) according to Ellman’s spectrophotometric method.^25^ Initially, for each compound, the percentages of inhibition were determined at the inhibitor concentration equal to 9 μM and, for the most potent compounds, also at 900 and 90 nM in the presence of 0.0833 U/mL of the enzyme and 100 μM of acetylthiocholine.

Based on the results illustrated in Table 1, related to pyrimidine diamine derivatives, there are no significant differences between the percentages of inhibition toward *Ee*AChE of the compounds having an aliphatic linker with five methylene units (**9**–**12**) compared to the corresponding derivatives with six methylene units (**13**, **14**, **16**, and **21**). On the other hand, toward *eq*BChE, the compounds having a five-methylene chain show a lower inhibitory potency compared to the six methylene derivatives. As a consequence of this observation, for the synthesis of the subsequent compounds, we decided to use an aliphatic linker with six methylene units.

**Table 1:**
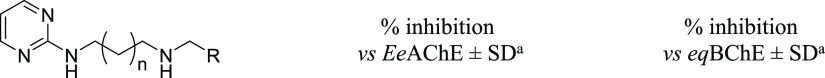

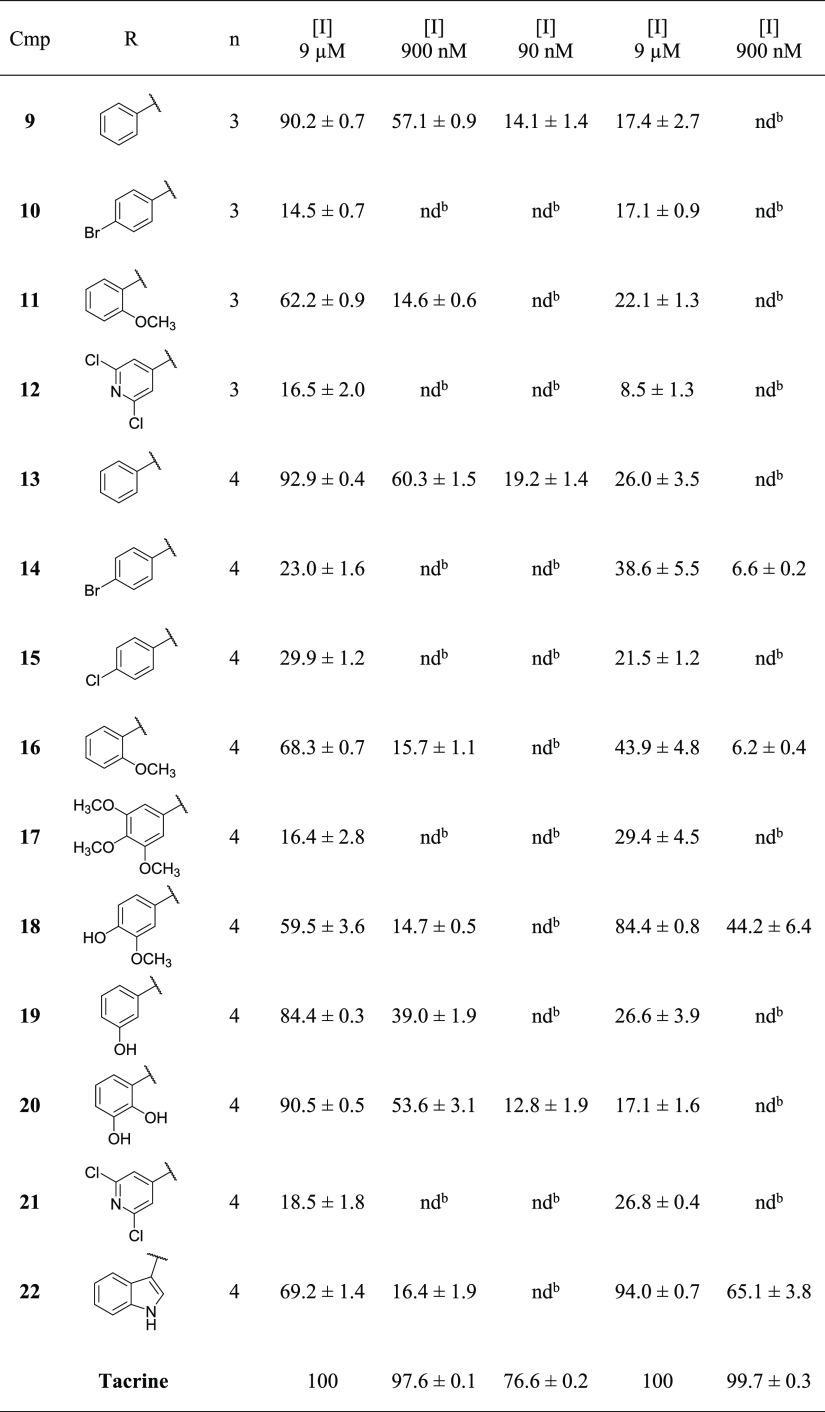
Inhibition of *Ee*AChE and *eq*BChE Activities by Pyrimidine Diamine Derivatives **9**–**22**

a Data are the average of three replicates.

b nd stands for not determined.

Among pyrimidine diamine derivatives, the most potent compounds on *Ee*AChE, with approximately 90% of inhibition at 9 μM and between 40 and 60% at 900 nM, are **9** and **13**, both with an unsubstituted phenyl ring on one side of the aliphatic chain, **19**, with a 3-hydroxyphenyl ring, and **20**, having a 2,3-dihydroxyphenyl ring. The insertion of a halogen atom in the para position of the phenyl ring (compounds **10**, **14**, and **15**) as well as the replacement of the phenyl with the 3,4,5-trimethoxyphenyl (**17**) or 2,6-dichloropyridine ring (**12** and **21**) causes a drastic reduction of the inhibitory potency on *Ee*AChE; the replacement of the phenyl with the 2-methoxyphenyl (**11** and **16**), 3-methoxy-4-hydroxyphenyl (**18**), or indole (**22**) ring reduces the percentages of inhibition toward *Ee*AChE from 90% to 60–70% at an inhibitor concentration of 9 μM.

Generally, these pyrimidine diamine derivatives have low inhibitory potency toward *eq*BChE (8–45% at 9 μM), with the exception of compound **18**, with the 3-methoxy-4-hydroxyphenyl ring, which shows 84% inhibition at 9 μM and 44% at 900 nM, and **22**, with the indole group, having a 94% inhibition at 9 μM and 65% at 900 nM.

Among pyridine diamine derivatives, whose data are reported in Table 2, the compounds having an aliphatic chain with five methylene units (**23** and **24**) show a lower inhibitory potency toward both enzymes compared to the corresponding derivatives with a six-methylene chain (**25** and **26**). Generally, pyridine diamine derivatives have similar or lower percentages of inhibition on *Ee*AChE compared to the corresponding pyrimidine derivatives (Tables 1 and 2) with the exception of **29** and **33**, having the 3,4,5-trimethoxyphenyl or 2,6-dichloropyridine ring, respectively. On the contrary, pyridine derivatives are more potent on *eq*BChE than the corresponding pyrimidine compounds. Among pyridine derivatives, the most potent compound on *Ee*AChE is **25**, having an unsubstituted phenyl ring on one side of the aliphatic chain, with a 73% inhibition at 9 μM. Among the compounds with six methylene units, the insertion of a halogen atom in the para position on the phenyl ring (**26** and **27**) and the replacement of the phenyl with the 3,4,5-trimethoxyphenyl group (**29**) cause a reduction of the percent inhibition at 9 μM on *Ee*AChE from 73% to 24–30%. Furthermore, the replacement of the phenyl with the 2-methoxyphenyl (**28**), 3-methoxy-4-hydroxyphenyl (**30**), or 2,3-dihydroxyphenyl (**32**) ring reduces the inhibition at 9 μM to about 60%, while the replacement with the 3-hydroxyphenyl (**31**) or 2,6-dichlropyridine (**33**) ring reduces inhibition to about 40%. On *eq*BChE, the most potent compound is **30**, with the 3-methoxy-4-hydroxyphenyl ring, that shows 91% inhibition at 9 μM and 51% at 900 nM.

**Table 2:**
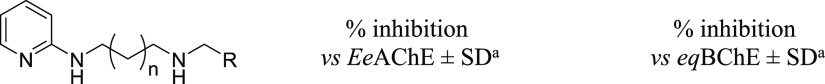

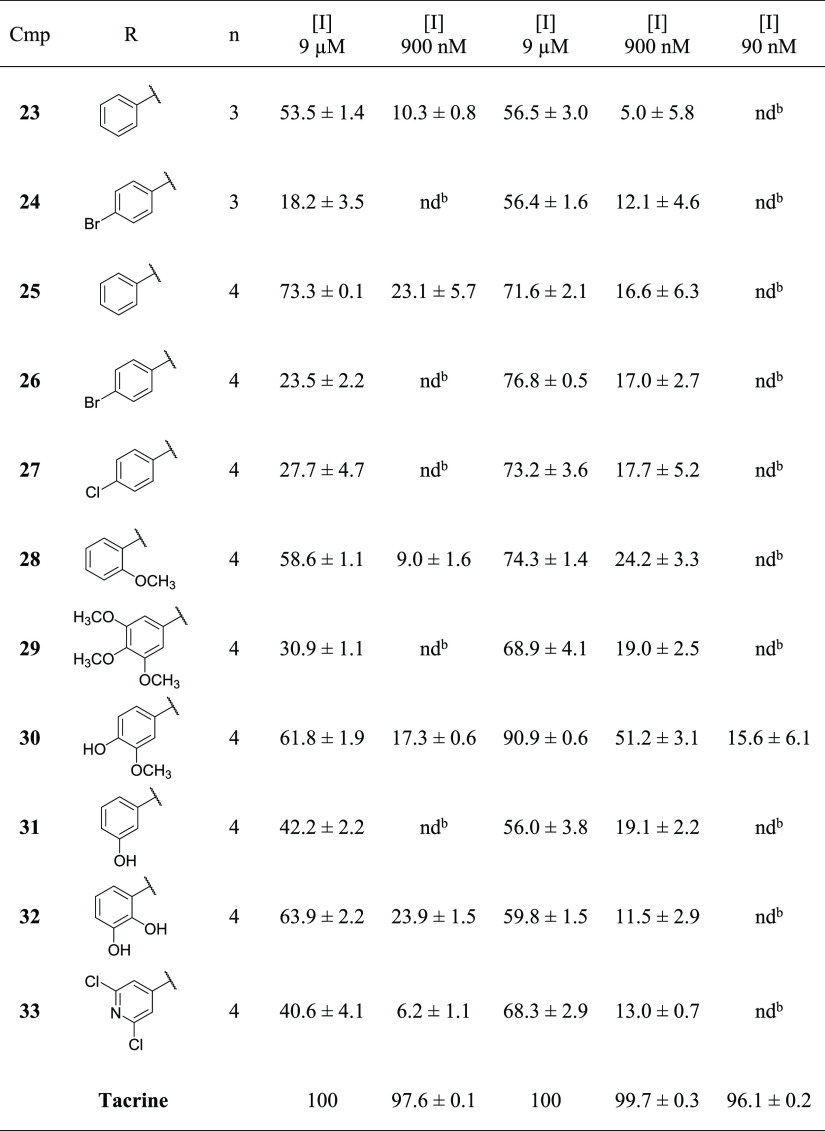
Inhibition of *Ee*AChE and *eq*BChE Activities by Pyridine Diamine Derivatives **23**–**33**

a Data are the average of three replicates.

b nd stands for not determined.

For a selection of compounds, among the most potent as inhibitors of *Ee*AChE and/or *eq*BChE, the inhibition constant (*K*_i_) and the corresponding inhibition mechanism were determined according to Dixon’s method,^26^ reporting in graph the reciprocal of the hydrolysis rate *vs* the inhibitor concentrations at a fixed concentration of the substrate. The recorded data were analyzed with the enzyme kinetic module of SigmaPlot to find the best fitting model of inhibition using the linear regression analysis. The reference kinetic models used in the regression analysis were competitive, noncompetitive, uncompetitive, and mixed. Each determination was repeated five times, and incorrect values were discarded to reduce the standard deviation within the limit of 5%. In this way, the regression lines obtained have a linear regression coefficient (*R*^2^) higher than 0.95. Dixon’s plots of all tested compounds are reported in the Supporting Information (Figures S1–S12).

The tested compounds revealed a mixed or uncompetitive inhibition mechanism toward *Ee*AChE and *eq*BChE, with *K*_i_ according to the order of low micromolar or nanomolar (Table 3). The mixed inhibition mechanism might suggest an interaction of the compound with both CAS and PAS of the enzyme and a possible involvement in the inhibition of Aβ plaque formation induced by AChE. For compounds inhibiting the enzymes with a mixed mechanism, Table 3 also reports the values of α, which indicate whether the inhibitor preferentially binds to the free enzyme (α > 1) or to the enzyme–substrate complex (α < 1).

**Table 3:** Inhibition Mechanisms Determined on *Ee*AChE and *eq*BChE, Inhibition Constants (*K*_i_), and α Values

	*Ee*AChE						*eq*BChE			
cmpd	mechanism	*K*_i_ ± SD^a^ (μM)	*R*^2^	α		cmpd	mechanism	*K*_i_ ± SD^a^ (μM)	*R*^2^	α
**9**	mixed	0.312 ± 0.108	0.982	7.5		**18**	mixed	3.034 ± 0.604	0.986	7.3
**13**	mixed	0.426 ± 0.132	0.991	0.3		**22**	mixed	0.099 ± 0.071	0.990	10
**19**	uncomp.	0.509 ± 0.018	0.992			**25**	mixed	2.373 ± 0.304	0.992	2.2
**23**	mixed	0.743 ± 0.316	0.983	2.2		**26**	mixed	3.465 ± 1.480	0.950	0.5
**25**	mixed	0.995 ± 0.374	0.988	0.8		**28**	mixed	3.434 ± 0.701	0.988	0.8
**28**	mixed	1.323 ± 0.622	0.990	1.2		**30**	mixed	1.105 ± 0.189	0.983	1.4

a Each data was obtained with three substrate and five inhibitor concentrations; each measurement was carried out in five replicates (see the Experimental Section).

The pyrimidine amine compounds are more potent on *Ee*AChE than the pyridine analogs, as confirmed by *K*_i_ values of **9** and **13** (pyrimidine derivatives with the phenyl ring on one side of the aliphatic chain, respectively, with five or six methylene units) compared to *K*_i_ values of **23** and **25** (pyridine derivatives with the phenyl ring on one side of the aliphatic chain, respectively, with five or six methylene units). All the compounds tested on *Ee*AChE show a mixed inhibition mechanism, with the exception of **19**, which acts with an uncompetitive mechanism. The most potent inhibitors of *Ee*AChE are compounds **9** and **13**, with *K*_i_ values of 312 ± 108 and 426 ± 132 nM, respectively.

Generally, pyridine amine derivatives are more potent on *eq*BChE than the corresponding pyrimidine derivatives. All the compounds tested on *eq*BChE show a mixed inhibition mechanism. Compound **22** (a pyrimidine derivative with the indole group on one side of the aliphatic chain) is the most potent inhibitor of *eq*BChE, with *K*_i_ equal to 99 ± 71 nM.

For the pyrimidine amine derivative **20**, with a 2,3-dihydroxyphenyl ring on one side of the aliphatic chain, it was necessary to determine the IC_50_ on *Ee*AChE (the concentration of inhibitor required to inhibit 50% of the enzyme) instead of the *K*_i_ due to the instability of this molecule in the aqueous solution for the time required for *K*_i_ determination. The IC_50_ value was obtained by plotting the percentages of inhibition toward *Ee*AChE *vs* the concentration of inhibitor expressed in a logarithmic scale at a fixed substrate concentration (100 μM). The recorded data were analyzed with the enzyme kinetic module of SigmaPlot. All measurements were replicated three times, and the IC_50_ value obtained was confirmed by repeating the experiment twice. For compound **20**, the IC_50_ on *Ee*AChE is 622 ± 30 nM, as shown in Table 4. The IC_50_ plot of the tested compound is reported in the Supporting Information (Figure S13).

**Table 4:** IC_50_ on *Ee*AChE for Compound **20** and on Both ChEs for Tacrine and Donepezil, Used as the Reference Standard

cmpd	*Ee*AChE, IC_50_ ± SE (nM)	*eq*BChE, IC_50_ ± SE (nM)
**20**	621.9 ± 29.5	nd^a^
tacrine	41.0 ± 4.9	3.7 ± 0.5
donepezil	16.2 ± 1.8	1727 ± 300

a nd stands for not determined.

### Molecular Docking Studies

Molecular modeling analyses were performed to provide structural insights on the binding mode of the investigated compounds into the *h*AChE and *h*BChE catalytic pocket. For the current study, the X-ray crystallographic structures of the *h*AChE in complex to donepezil (PDB code:4EY7)^13^ and the *h*BChE in complex to the naphthamide derivative (PDB code:5NN0)^27^ were used. As there is no crystal structure of *eq*BChE in the protein data bank (PDB), *h*BChE was used for the computational study. Indeed, the sequence of *eq*BChE derived from the Uniprot Database shares 90% sequence identity with that of the adopted *h*BChE (PDB code:5NN0), above all compared residues of the binding site (Figure S14). Regarding AChE, even if the apo-crystal structures of *Ee*AChE are present in PDB, they cannot be considered as good quality models. After the alignment of *Ee*AChE (PDB code:1C2O) with the adopted *h*AChE structure (PDB code:4EY7), 94.7% of the identity of the aligned sequences within a range of 7 Å from the binding site is obtained (Figure S15). According to a previous work^23^ and the criteria for the identification of reliable complexes reported in the literature,^28^ the selected models were considered as a good starting point for *in silico* investigation.

Our docking protocol was validated by docking co-crystallized ligands into the binding site. Root mean square deviation (RMSD) values between the native pose of *h*AChE and *h*BChE ligands and the related best redocked conformations were found to be 0.12 and 0.96 Å, respectively, thus revealing the reliability of the docking protocol (Figure S16). According to previously published studies,^23,29^ no linear correlation between docking score and the experimental data is expected (Table S1). Nevertheless, the obtained docking poses for the analyzed compounds were comparable with the binding mode of donepezil into AChE (Figure S17). It is widely known that donepezil is a dual binding site inhibitor, i.e., engaging simultaneously π–cation and π–π interactions with Trp86 and Trp286 of the CAS and the PAS, respectively. Moreover, the ketone group of the indanone ring of donepezil formed a hydrogen bond with Phe295 of the mid-gorge binding site. In detail, the CAS region is characterized by Trp86, Tyr119, Tyr124, Tyr133, Glu202, Ser203, Trp439, His447, and Tyr449, and the PAS region is composed of Tyr72, Asp74, Thr75, Trp286, Leu289, Tyr341, and Val365 residues. The mid-gorge pocket of *h*AChE consists of Leu76, Phe295, Arg296, Phe297, Tyr337, and Phe338, and it is about 20 Å deep by an average of 13 Å wide.

Particularly, it is shown that, regardless of the aliphatic chain length, the protonated amine group interacted with Trp86 of the anionic site of CAS for all the docked compounds except for compound **29**, which interacted with Tyr341 and Tyr337 of the mid-gorge site.

**Figure 2:**
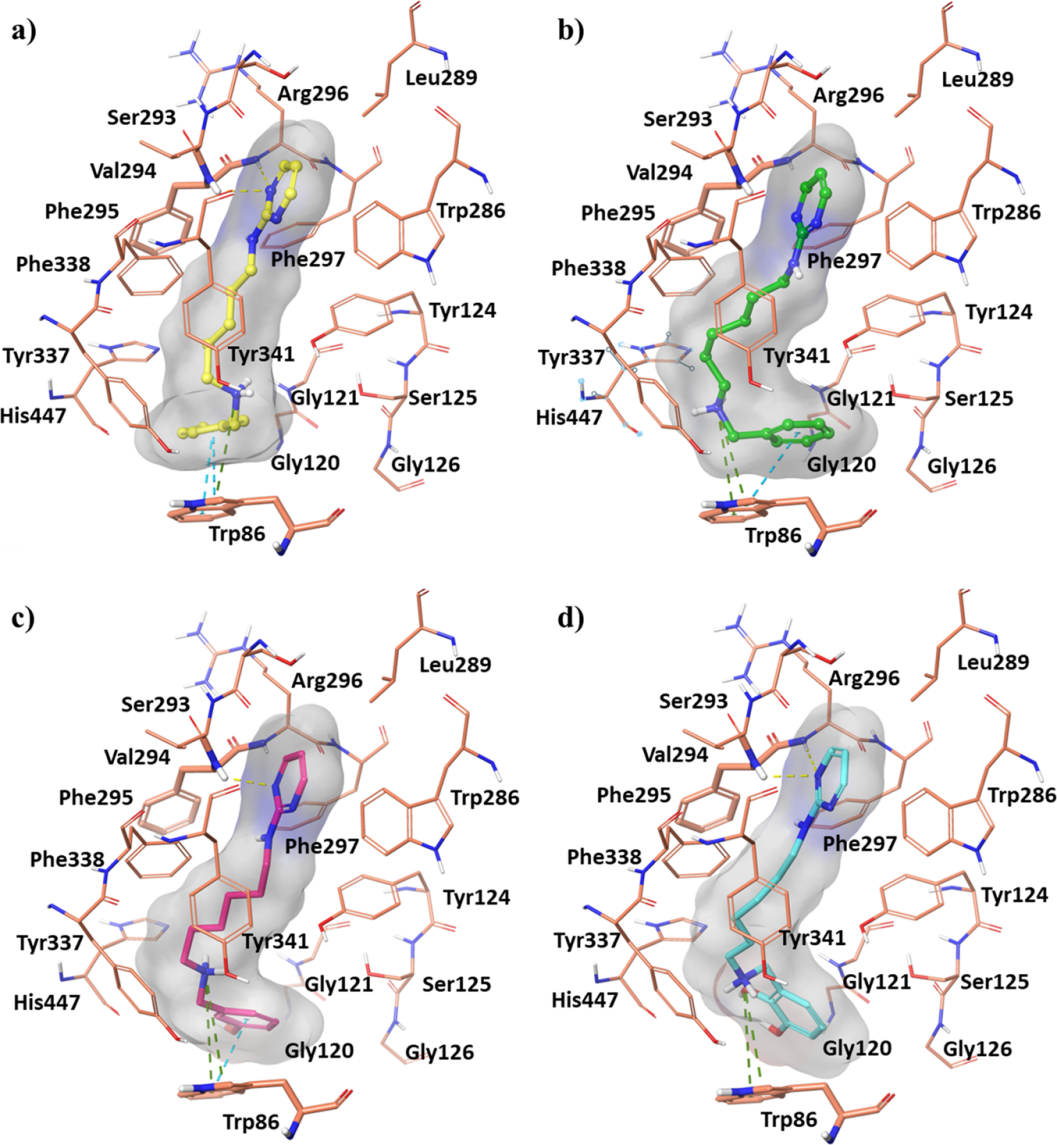
Best docking pose of compounds: (a) **9**, (b) **13**, (c) **19**, and (d) **20** into the *h*AChE active site shown as wire representation. Most relevant interacting residues are displayed as thin salmon tubes. Compounds **9**, **13**, **19**, and **20** are illustrated in yellow, green, pink, and blue carbon ball and stick representation, respectively. Hydrogen bonds, π–π interactions, and π–cation contacts are, respectively, represented by yellow, blue, and green dotted lines.

**Figure 3:**
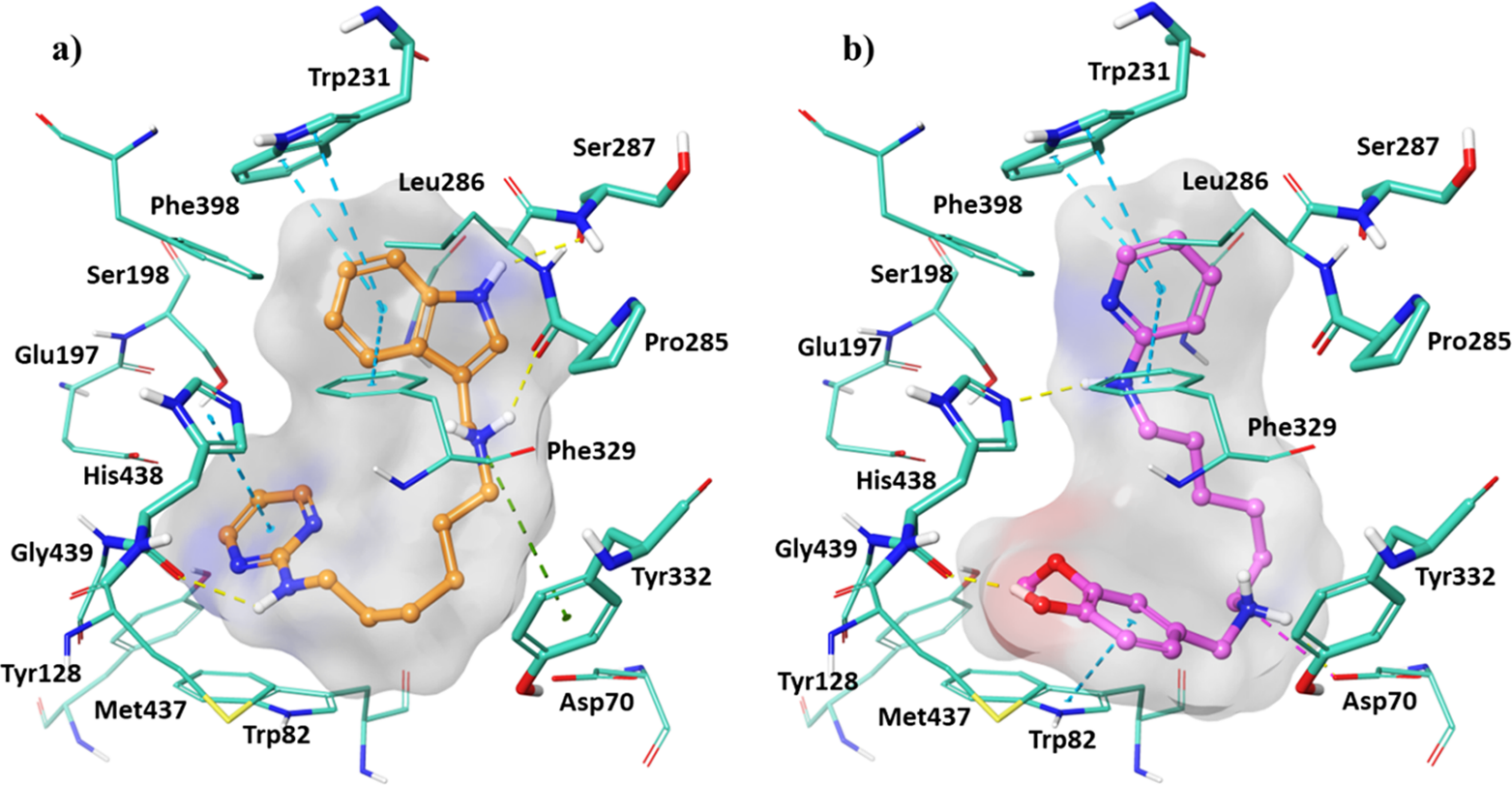
Best docking pose of compounds: (a) **22** and (b) **30** into *h*BChE active site shown as wire representation. The most relevant interacting residues are displayed as thin light green tubes. Compounds **22** and **30** are illustrated in orange and pink carbon ball and stick representation, respectively. Hydrogen bonds, π–π interactions, and π–cation contacts are, respectively, represented by yellow, blue, and green dotted lines.

Both 2-amino-pyrimidinic and 2-amino-pyridinic moieties exhibited hydrogen bonds with Phe295 and Arg296 in the mid-gorge pocket, except for compound **13**, whose pyrimidine cycle was more exposed to the solvent. The introduction of the other aromatic ring contributed to the stabilization of the compounds in the CAS region. Molecular recognition studies revealed the major binding interactions between our most potent inhibitors with *h*AChE (compounds **9**, **13**, **19**, and **20**) (Figure 2) and *h*BChE (compounds **22** and **30**) (Figure 3).

The compound **9** pyrimidine group formed two hydrogen bonds with Phe295 and Arg296 (Figure 2a). The phenyl ring also created a π–π stacking interaction with Trp86, while the protonated amine contributed to the accommodation at the CAS and PAS sites by means of a π–cation with Trp86 and a salt bridge with Asp74, respectively. The aliphatic extension of one methylene unit in compound **13** (Figure 2b) induced a destabilization of the hydrogen bonds between the pyrimidine portion and mid-gorge amino acids. Conversely, this orientation allowed a π–cation interaction between the protonated amine group and the indole ring of Trp86, with the loss of the interaction with Asp74 observed for compound **9**, still maintaining the π–π interaction between the phenyl group and Trp86.

Concerning compounds **19** and **20** (Figure 2c,d), they showed very similar docking poses orientating the protonated amine group toward Trp86 and interacting with Tyr133 and Glu202 through two and three hydrogen bonds with the phenolic and cathecolic group, respectively.

As regard *hB*ChE, all the investigated compounds were able to accommodate into the binding cavity with a lower binding affinity with respect to that of *h*AChE due to the presence of more hydrophilic and charged residues in the PAS and mid-gorge site.

In detail, the pyrimidine moiety of compound **22** (Figure 3a) created a π–π interaction with His438, and this residue also engaged a hydrogen bond with the amine group. Furthermore, a π–cation and a hydrogen bond were formed between the ligand protonated amine and Tyr332 and Pro285, respectively. The indole moiety pointed to Trp231 in CAS and Phe329 in mid-gorge regions, providing also a hydrogen bond with Ser287. Compound **30** (Figure 3b) showed a different docking pose orientating the pyridine toward Trp231 and Phe329. It interacted with His438 by means of the hydroxyphenyl at position 4, while the amine group bound to the pyridine. Its 3-methoxy-4-hydroxyphenyl ring was able to engage a π–π stacking interaction with Trp82. With Asp70, belonging to the PAS region, that phenolic moiety established one hydrogen bond and the protonated amine group an additional salt bridge.

### Molecular Dynamic (MD) Studies

The best docked poses of compounds **9**, **13**, **19**, and **20** into binding pockets of *h*AChE and compounds **22** and **30** into that of *h*BChE were submitted to 250 ns of molecular dynamic (MD) simulations. The results of MDs were investigated in terms of stability of the complexes and conformational flexibility of *h*ChEs in the presence of the promising inhibitors by monitoring the single contributions of hydrophobic, water-bridge, and hydrogen bonding interactions.

**Figure 4:**
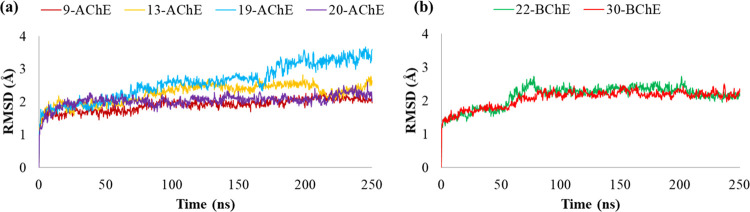
(a) RMSD of compounds **9**, **13**, **19**, and **20** and backbone atoms of *h*AChE. (b) RMSD of compounds **22** and **30** and backbone atoms of *h*BChE.

The stability of the *h*ChEs complexes trajectories can be monitored by the RMSD of the protein’s backbone atoms from its initial to final conformation over an MD time of 250 ns (Figure 4). As shown, the RMSD plot indicated that all compounds in complex to *h*AChE (Figure 4a) and *h*BChE (Figure 4b) maintained overall stability during the whole MD, thus ensuring a good equilibrium for the systems. Particularly, compounds **9**, **13**, and **20** in complex to *h*AChE were associated with increased conformational stability with RMSD average values of 1.89, 2.25, and 2.04 Å, respectively, in comparison to compound **19** (2.60 Å). As to compounds **22** and **30** in complex to *h*BChE, a similar stability trend was observed, resulting in very low fluctuations and RMSD average values of 2.13 and 2.06 Å, respectively.

The compound **9** protonated amine group interacted mainly with the amino acids of both CAS and PAS regions of the *h*AChE pocket. Indeed, Asp74, Trp86, His447, and Tyr337 for 46, 53, 38, and 46% of the MD time were engaged, respectively. The pyrimidine ring also formed a π–π interaction with Tyr341 (35%) of the mid-gorge site. By carefully looking at compound **13** accommodation into the *h*AChE pocket during MD, it can be observed that the protonated amine group participated in major interactions with Trp86 (24%), Glu202 (96%), His447 (17%), and Gly448 (97%). Tyr124 also provided a significant hydrogen bond in the overall simulation (96%), while Phe297 and Tyr341 engaged π–π stacking interactions with the pyrimidine moiety and one of its nitrogen atoms displayed two water bridges, with Phe295 (42%) and Arg296 (21%), located at the mid-gorge. Finally, the phenyl ring was surrounded by the aromatic residues of the CAS region through a hydrophobic interaction with Trp86 and Tyr449 for 35 and 41% of the MD time. The hydroxyphenyl in compound **19** and the 2,3-dihydroxyphenyl ring in compound **20** were inserted into the CAS region, interacting with Tyr133 for 84% and with Glu202 for 67% of the MD time, respectively. Also, the 2,3-dihydroxyphenyl ring formed a strong π–π stacking interaction with Trp86 during the whole trajectory. The pyrimidine moiety formed a water bridge with Phe295 for 33 and 58% of MDs for compounds **19** and **20**, respectively. The last one was also stabilized by the hydrogen bond between the nitrogen atom of the pyrimidine ring and Tyr124 at the CAS site (62%). The protonated nitrogen of compound **19** exhibited a noticeable 96% π–cation with Trp86, 89% hydrogen bond with Glu202, and 36% water-bridge interaction with His447 residue, while, in compound **20**, the positively charged group displayed long persistency in the interaction with Asp74 (69%), Trp86 (66%), and Ser125 (86%).

Analyzing the main interactions of compounds **22** and **30** during MDs, it was possible to observe the effect of the different orientation of the former *vs* the latter in *h*BChE.

Indeed, the indole moiety of compound **22** engaged hydrophobic interactions with Trp231 (45%) and Phe329 (28%), a hydrogen bond with Ser287 (44%), and a water bridge with the Pro285 residue in CAS and mid-gorge pockets. The flexibility of the ligand allowed one to orient the protonated amine group toward the catalytic region forming a hydrogen bond with His438, a water bridge with Glu197, and a salt bridge with Trp82 at the CAS site. The insertion of pyrimidine contributed to the stabilization of *h*BChE into the CAS region due to the interaction with Trp82, Tyr440, and Trp430. Instead, the opposite orientation of compound **30** favored the interaction of the pyridine ring in the π–π stacking interactions with Trp231 and Phe329 for 50 and 69% of the time of the simulation. This also directed the amino group toward Ser198 (20%) of the catalytic region and the 3-methoxy-4-hydroxyphenyl ring toward His438 (37%). Such rearrangement led to the protonated amine group anchoring to Asp70 (10%) and Trp82 (35%) at PAS and CAS sites, respectively.

In summary, MD results reveal the role of the pyrimidine ring, protonated amine group, and substituted aromatic moiety of the pyrimidine derivatives in occupying CAS, PAS, and mid-gorge cavities, thus suggesting their promising role as dual binding site inhibitors.

The protonated amine group inserted into linear alkyl chains of five or six methylene units is one of the crucial pharmacophore features for ChEs inhibition, and it mainly establishes strong interactions with the catalytic site and PAS region.

The pyrimidine diamine scaffold bearing the indole moiety and 3-methoxy-4-hydroxyphenyl ring drives the selectivity of the molecule toward BChE inhibition.

Instead, phenolic and catecholic rings represent high selectivity for AChE inhibition, strongly supported by the interaction with Tyr133 and Glu202 of the AChE binding pocket.

Finally, the phenyl ring of compounds **9** and **13**, which differ only in the length of the alkyl chain, is well stabilized into the bottom of the gorge.

### Chelation Studies

For a selection of compounds, among the most potent as inhibitors of *Ee*AChE and/or *eq*BChE, metal chelation studies were performed by using UV–vis spectrophotometric analysis, a method widely used for investigating chelates, to define their possible multitarget profile.^30,31^

The chelating ability of pyrimidine and pyridine compounds **9**, **13**, **18**, **19**, **20**, **25**, **28**, and **30** on bio-metals Fe^3+^, Cu^2+^, and Zn^2+^ was evaluated. Initially, the UV–vis spectrum of the ligand was recorded and compared with the spectra obtained by adding an excess of metal to the ligand solution, maintaining the same concentration of ligand (ligand/metal ratio 1:3, 1:5, or 1:10). The variation of the UV–vis spectrum of the ligand in the presence of metal ions is indicative of the formation of the complex. Based on this, it has been observed that all the tested pyrimidine and pyridine derivatives have the ability to chelate Fe^3+^ and Cu^2+^ ions, while only compound **20** shows a chelating capacity also on Zn^2+^. Based on these results, it can be stated that the chelating activity of these compounds is due to the presence of two adjacent nitrogen atoms in the 2-aminopyrimidine or 2-aminopyridine moiety. For derivatives **18**–**20** and **30**, it is also possible that the phenolic portion participates in the formation of the complex. In particular, the catechol ring in **20** could be responsible for the ability of this molecule to chelate also Zn^2+^.

**Figure 5:**
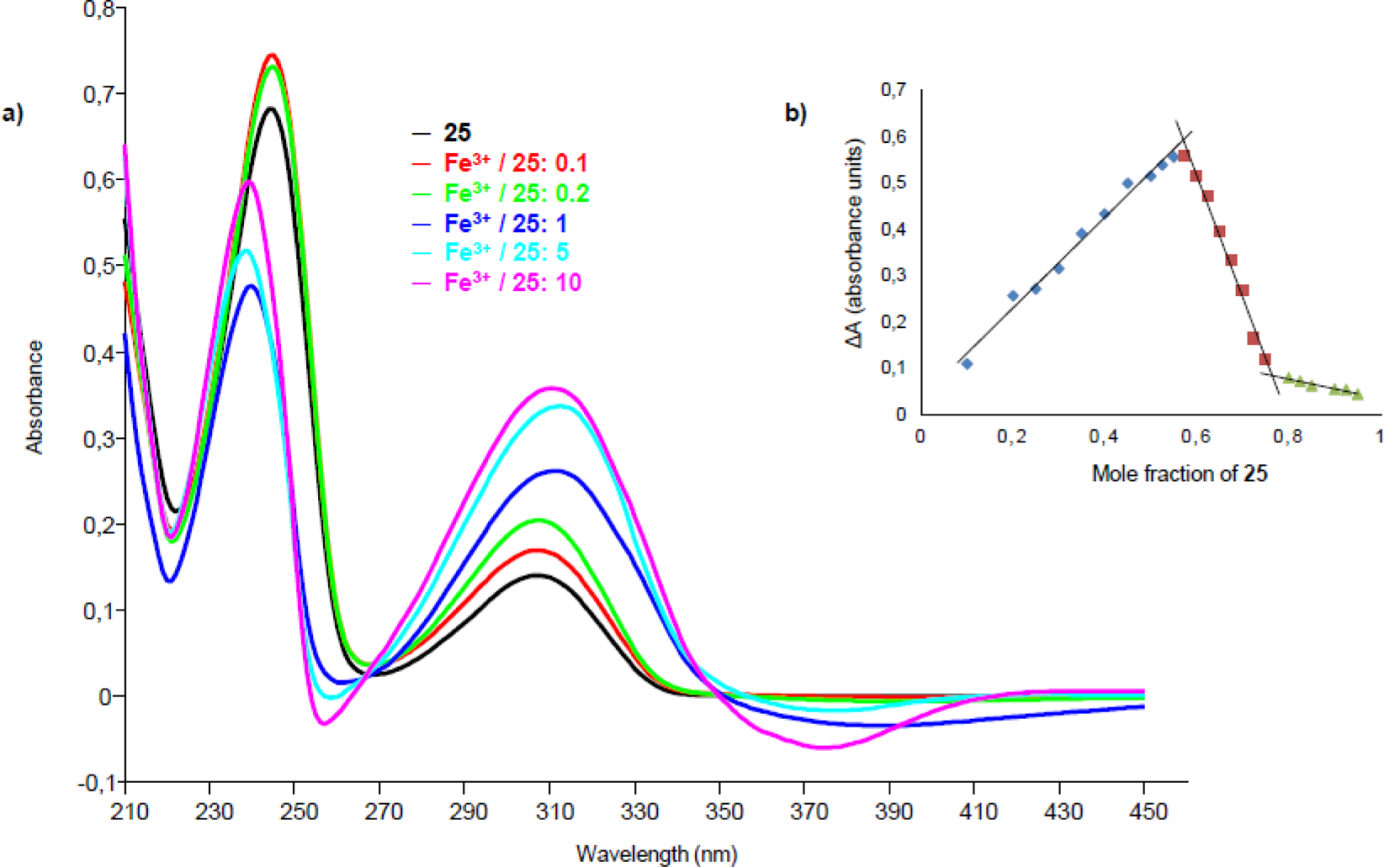
(a) UV–vis titration of ligand **25** with Fe^3+^. The greatest variations in spectra with increasing amount of metal are observed at 251 and 374 nm, with a reduction of absorbance, and at 318 nm, with an increase of absorbance. There are two isosbestic points at 270 and 350 nm. (b) Job’s plot of compound **25** in the presence of Fe^3+^: variation of the absorbance (Δ*A*) at the wavelength of 318 nm, in the ordinate, *vs* the mole fraction of **25**, in the abscissa. *X*_1_ (mole fraction that causes the maximum variation of absorbance) = 0.57; *X*_2_ = 0.77; *n*_1_ (number of ligand molecules per cation) = 1; and *n*_2_ = 3.

UV–vis titrations of these compounds were carried out with the metal ions with which they form complexes, recording first the UV–vis spectrum of the ligand and then the spectra obtained by mixing solutions of the ligand and metal according to increasing metal/ligand molar ratios (Figure 5a).

The stoichiometries of metal–ligand complexes were determined through Job’s method.^32,33^ In Job’s plot, the values of Δ*A*, measured at the wavelengths where evident absorbance variations were observed in the titration spectra, are in the ordinate and the mole fractions of the ligand are in the abscissa. The mole fraction *X*, which causes the maximum variation of absorbance, is extracted from the graph and then the value of the coefficient *n*, which corresponds to the number of ligand molecules per cation, is obtained (Figure 5b). The UV–vis titration spectra and Job’s plots of all the tested compounds are reported in the Supporting Information (Figures S18–S46), while the data extrapolated from the Job’s plots are summarized in Table 5.

**Table 5:** Job’s Plot Data for the Tested Compounds in the Presence of Fe^3+^ and Cu^2+^^a^

	Fe^3+^			Cu^2+^		
cmpd	λ (nm)	*X*	*n*	λ (nm)	*X*	*n*
**18**	375	0.69	2	330	0.70	2
**19**	375	0.68	2	330	0.51	1
**20**	370	*X*_1_ = 0.53	*n*_1_ = 1	330	0.53	1
		*X*_2_ = 0.80	*n*_2_ = 4			
**25**	318	*X*_1_ = 0.57	*n*_1_ = 1	330	0.44	1
		*X*_2_ = 0.77	*n*_2_ = 3			
**28**	316	*X*_1_ = 0.53	*n*_1_ = 1	330	0.53	1
		*X*_2_ = 0.74	*n*_2_ = 3			
**30**	310	*X*_1_ = 0.53	*n*_1_ = 1	312	0.50	1
		*X*_2_ = 0.74	*n*_2_ = 3			

a The table shows the wavelength values (λ) in which the absorbance variation was measured, the molar ratios (*X*) that cause the maximum variation of absorbance, and the coordination values (*n*) that correspond to the number of ligand molecules per cation.

### Antioxidant Activity

To characterize multitarget compounds, antioxidant activity assays were carried out on **18**, **19**, **20**, and **30** since they are the most potent ChEIs among phenolic derivatives, they exert chelating activity on metal ions involved in AD, and they have a structure that could justify an antioxidant activity.

The antioxidant activity assays were carried out according to the 2,2-diphenyl-1-picrylhydrazyl (DPPH) spectrophotometric method.^34^ Initially, to assess whether the compounds had antioxidant activity and to define the time required by each antioxidant to reach the steady state, the reduction in absorbance over time at 515 nm of a solution of DPPH mixed with the tested compound was recorded until reaching the plateau. From these measurements, compound **19** did not show a significant ability to interact with DPPH since a reduction of the absorbance over time was not observed; otherwise, compounds **18**, **20**, and **30** possess antioxidant activity as a reduction in absorbance over time, until reaching the plateau (in 1 min for **2** and 90 min for **18** and **30**), was observed. Subsequently, the EC_50_, defined as the ratio of moles of antioxidant that reduce by 50% the initial concentration of DPPH to initial moles of DPPH,^35^ was determined for compounds **18**, **20**, and **30**. The value of EC_50_ can be extrapolated by plotting the percentage of residual DPPH at the steady state as a function of the molar ratio of the antioxidant to initial DPPH.

Based on the obtained results reported in Table 6, the more efficient antioxidant, even better than ascorbic acid, which was used as the positive control, is the catechol derivative **20** both for the high reaction rate with DPPH and for the EC_50_ value equal to 0.1366 ± 0.0060. Compounds **18** and **30**, having the 3-methoxy-4-hydroxyphenyl ring, show antioxidant capacity lower than **20** and comparable to each other, with EC_50_ respectively equal to 0.5997 ± 0.0940 and 0.6419 ± 0.0730.

**Table 6:** EC_50_ Values and Times for Reaching the Plateau for the Tested Compounds

compound	EC_50_ ± SD^a^	reaction time (min)
**18**	0.5997 ± 0.0940	90
**20**	0.1366 ± 0.0060	1
**30**	0.6419 ± 0.0730	90
ascorbic acid	0.2650 ± 0.0070	2

a EC_50_, defined as the ratio of antioxidant moles necessary to reduce the initial concentration of DPPH by 50% to the initial moles of DPPH. Data are the average of three independent assays.

### Inhibition of Amyloid and Tau Aggregation

For the most potent ChEIs, the direct anti-amyloid aggregation activity against Aβ_42_ and tau aggregation was evaluated. The anti-aggregating effect of the tested compounds was monitored by a cell-based assay in intact *Escherichia coli* cells that overexpress either the Aβ_42_ peptide or tau protein, which upon overexpression form insoluble inclusion bodies that were stained with thioflavin-S, an amyloid specific dye.^36^ The percentages of inhibition toward Aβ_42_ and tau aggregation were determined at an inhibitor concentration of 100 μM, and the obtained results are reported in Table 7. Low percentages of inhibition were found both on Aβ_42_ (up to 22%) and on tau (up to 17%), so these compounds have a weak anti-aggregating activity when compared with the reference compound DP-128.^37^ The pyridine derivative **28** is the best inhibitor of aggregation of both Aβ_42_ and tau, while the pyrimidine **13** showed a similar percentage of inhibition on Aβ_42_ aggregation but lower toward tau.

**Table 7:** Inhibition of Aβ_42_ Aggregation and Tau Aggregation by the Tested Compounds

	Aβ_42_ aggregation		tau aggregation	
cmpd	% inhibition [I] = 100 μM	SEM^a^	% inhibition [I] = 100 μM	SEM^a^
**9**	5.8	1.6	2.6	6.7
**13**	21.0	4.2	12.6	6.6
**18**	1.7	2.1	1.0	4.1
**19**	16.7	2.7	9.9	3.9
**20**	7.2	2.4	4.6	1.0
**22**	9.7	1.8	6.8	6.1
**23**	10.4	3.9	7.0	4.2
**25**	16.8	2.9	6.7	2.9
**26**	7.3	3.7	7.3	4.8
**28**	22.3	3.3	17.0	6.4
**30**	13.6	5.3	12.8	2.7
ref	98.8	1.0	94.7	3.1

a A minimum of five independent assays (with three replicates for assay) was performed for each tested compound. More assays were performed to obtain a SEM < 5% with a maximum of 10 independent assays. As a reference compound, we have used the known anti-amyloid drug DP-128 at 100 μM.

### Computation of Physicochemical Descriptors and ADME Parameters

Physicochemical descriptors and ADME parameters of the most interesting compounds, by virtue of their inhibitory activity toward ChEs, as well as chelating and antioxidant ability, were predicted by means of the SwissADME public server,^38^ and the obtained data are reported in Table 8. All the studied compounds fit Lipinski’s rule of five (MW ≤ 500; MLogP ≤ 4.15; H bond acceptor ≤ 10; and H bond donor ≤ 5).^39^ They should be soluble or moderately soluble in water. They should have high gastrointestinal absorption after oral administration. Worthily, these compounds should be BBB accessible, with the exception of **18** and **20**.

**Table 8:** Predicted Physicochemical Properties and ADME Parameters: Molecular Weight (MW); Number of H-Bond Acceptors (H-b Acc); Number of H-Bond Donors (H-b Don); Number of Heavy Atoms (Heavy Atoms); Number of Rotatable Bonds (Rot Bonds); Topological Polar Surface Area in Å^2^ (TPSA); Octanol/Water Partition Coefficient (MLogP); Water Solubility (LogS ESOL); Water Solubility Class (Sol Class); Gastrointestinal Absorption (GI); Blood–Brain Barrier Permeation (BBB); and Number of Lipinski’s Rule of Five Violations (Lipinski Viol)^a^

cmpd	MW	H-b acc	H-b don	heavy atoms	rot bonds	TPSA	MLogP	logS ESOL	sol class	GI	BBB	Lipinski viol
**9**	270.37	3	2	20	9	49.84	1.88	–3.04	soluble	high	yes	0
**13**	284.40	3	2	21	10	49.84	2.12	–3.26	soluble	high	yes	0
**18**	330.42	5	3	24	11	79.30	1.25	–3.19	soluble	high	no	0
**19**	300.40	4	3	22	10	70.07	1.56	–3.12	soluble	high	yes	0
**20**	316.40	5	4	23	10	90.30	1.01	–3.32	soluble	high	no	0
**22**	323.44	3	3	24	10	65.63	1.87	–3.63	soluble	high	yes	0
**23**	269.38	2	2	20	9	36.95	2.56	–3.45	soluble	high	yes	0
**25**	283.41	2	2	21	10	36.95	2.80	–3.67	soluble	high	yes	0
**26**	362.31	2	2	22	10	36.95	3.42	–4.57	moderately soluble	high	yes	0
**28**	313.44	3	2	23	11	46.18	2.45	–3.73	soluble	high	yes	0
**30**	329.44	4	3	24	11	66.41	1.89	–3.59	soluble	high	yes	0

a Data were obtained from the SwissADME public server.^38^

Furthermore, all synthesized final compounds were screened using *in silico* public tools, specified in the Experimental Section, and were not found to be pan assay interference compounds (PAINS),^40^ except for **20** and **32**. Nevertheless, the activities of these compounds are in line with those of the other pyrimidine and pyridine molecules studied and they can be framed in logical SAR, as previously discussed. Moreover, compound **20** showed a strongly different activity toward the related targets *Ee*AChE and *eq*BChE (percentages of inhibitions at 9 μM equal to 90.5 ± 0.5 and 17.1 ± 1.6, respectively; Table 1) and it was not cytotoxic at concentrations up to 10 μM, as reported in the subsequent section. Overall, these evidences suggested that these molecules do not act *via* a PAINS mechanism.

### Cytotoxicity Assays

**Figure 6:**
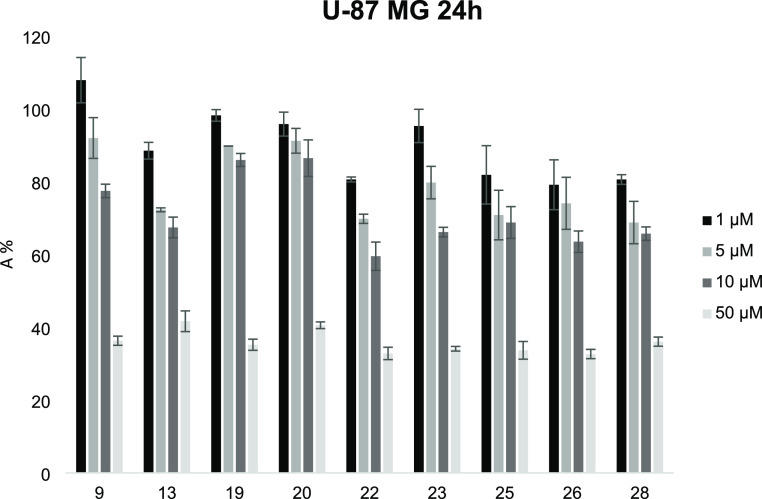
Cell viability assay of U-87 MG cells in the presence of increasing concentrations of studied compounds, evaluated using the MTT method. Data represent the absorbance % (± SD) relative to untreated cells in the same experiment and standardized to 100%. All data points were performed in triplicate and at least three independent experiments.

A selection of the most active inhibitors of *Ee*AChE and *eq*BChE (**9**, **13**, **19**, **20**, **22**, **23**, **25**, **26**, and **28**) was tested to evaluate the cytotoxic effects on the U-87 MG cell line from the human brain (glioblastoma astrocytoma) at concentrations ranging from 1 to 50 μM. The obtained data, represented in the histogram of Figure 6, suggest that the tested compounds are characterized by low toxicity toward the studied cells, in particular at concentrations up to 10 μM. In general, the pyrimidine derivatives appear to be less cytotoxic than pyridine derivatives, as evidenced by the comparison of pyrimidines **9** and **13** with the corresponding pyridines **23** and **25**. The pyridines tested showed a very similar toxicity profile, with the exception of compound **23** (having the spacer chain with five methylene units), which at concentrations of 1 and 5 μM showed a lower cytotoxic effect. Also, among pyrimidine derivatives, compound **9**, with five methylene units, exerted a minor effect on cell viability than the corresponding compound **13**, with six methylene units. Moreover, among pyrimidine derivatives, compound **22**, with an indole group on one side of the aliphatic chain, showed a more pronounced cytotoxic effect, with a toxicity profile similar to the tested pyridines.

## Conclusions

Series of pyrimidine and pyridine derivatives were designed as potential multifunctional compounds for AD, whose purpose could be to restore the cholinergic tone by inhibition of ChEs, attenuate the dyshomeostasis of the metals mainly involved in the pathology, reduce the oxidative stress, and contrast the toxicity and deposition of the Aβ peptide. These compounds contain two π systems, one of which is represented by a 2-aminopyrimidine or 2-aminopyridine moiety, connected to a small aromatic group by a flexible amino-alkyl linker.

On the synthesized compounds, enzymatic inhibition studies were carried out toward *Ee*AChE and *eq*BChE. The initial determination of the percentages of inhibition at concentrations equal to 9–0.09 μM led to the identification of the most potent inhibitors. For a selection of these compounds, the *K*_i_ were determined, obtaining values in the range of 0.312–1.323 μM *vs Ee*AChE and 0.099–3.465 μM *vs eq*BChE. All the tested compounds result in mixed inhibitors of ChEs, with the only exception of **19**, which acts with an uncompetitive mechanism toward *Ee*AChE. The uncompetitive inhibitors might interact with an allosteric modulation site such as PAS, while mixed inhibitors interact simultaneously with both CAS and PAS of the enzyme; these evidences may suggest that these compounds could interfere with the aggregation of Aβ plaques induced by AChE through interactions with PAS.

In general, the pyrimidine amine compounds are more potent on *Ee*AChE than the pyridine analogs. On the contrary, pyridine amine derivatives are more potent on *eq*BChE than the corresponding pyrimidine derivatives. The greater inhibitory potency toward *Ee*AChE is shown by compounds **9** (*K*_i_ = 0.312 ± 0.108 μM) and **13** (*K*_i_ = 0.426 ± 0.132 μM), both having the unsubstituted phenyl ring on one side of the aliphatic chain; indeed, modifications of the phenyl group, both adding substituents and changing the type of aromatic ring, reduce the inhibition activity toward *Ee*AChE. Differently, the most potent inhibitor of *eq*BChE is **22** (*K*_i_ = 0.099 ± 0.071 μM), the pyrimidine derivative with the indole ring on one side of the aliphatic chain.

*In silico* analyses highlighted that the protonated amine group inserted into the linear alkyl chain is the pivotal pharmacophore feature for ChE inhibition due to its strong interaction with the catalytic site and PAS region. Moreover, the pyrimidine diamine scaffold, bearing the indole moiety and 3-methoxy-4-hydroxyphenyl ring, resulted to drive the selectivity of the molecule toward BChE inhibition. On the other hand, phenolic and catecholic rings were responsible for the AChE inhibition selectivity, strongly supported by the interaction with Tyr133 and Glu202 of this enzymatic binding pocket. Additionally, the phenyl ring of compounds **9** and **13**, which differ only in the length of the alkyl chain, is well stabilized into the bottom of the gorge. Based on the computational results, the involvement of the pyrimidine ring, protonated amine group, and substituted aromatic moiety of these derivatives in occupying CAS, PAS, and mid-gorge cavities suggested their potential role as dual binding site inhibitors.

Metal chelation studies revealed that all the tested compounds have the ability to chelate Fe^3+^ and Cu^2+^ ions; this chelating activity can be attributed to the presence of two adjacent nitrogen atoms in the 2-aminopyrimidine or 2-aminopyridine moieties. The only 2-aminopyrimidine derivative that shows a chelating ability also toward Zn^2+^ ion is **20**, which contains a catechol group.

Moreover, antioxidant activity assays were carried out on compounds **18**, **19**, **20**, and **30** since they are the most potent ChEIs among phenolic derivatives. Catechol **20** is the most efficient antioxidant, even better than ascorbic acid, both for the high reaction rate with DPPH and for its EC_50_ value. Compounds **18** and **30**, having the 3-methoxy-4-hydroxyphenyl ring, show an antioxidant capacity lower than **20** and comparable to each other, while compound **19**, having the 3-hydroxyphenyl ring, has no significant antioxidant capacity.

Unfortunately, the tested compounds show low percentages of inhibition toward both Aβ_42_ and tau aggregation, proving to have a weak anti-aggregating activity in *E. coli.* However, the low anti-amyloid activity reported could also be due to an unusually low ability of the compounds to cross the bacterial wall. Although it is unusual, this effect has been observed recently and should not be ruled out.^41^

In conclusion, this research has allowed one to identify interesting lead compounds for the development of new multifunctional molecules useful for AD by virtue of their inhibitory activity toward ChEs, as well as for chelating and antioxidant abilities, low toxicity, and good predicted ADME parameters.

## Experimental Section

### Chemistry: General

All reagents and solvents were of analytical grade and were purchased from Sigma-Aldrich (Milano, Italy) or from Fluorochem (Hadfield, UK). Dichloromethane was dried by storing it over activated 4 Å molecular sieves (10% m/v). Triethylamine was freshly purified by distillation over potassium hydroxide. Column chromatographies were performed on a silica gel (Merck; 63–200 μm particle size).^1^H NMR and ^13^C NMR spectra were acquired at 25 °C, unless otherwise specified, on a Bruker AVANCE-400 spectrometer at 9.4 T operating at 400 MHz (^1^H NMR) and 100 MHz (^13^C- MR); chemical shift values (δ) are given in ppm relative to TMS, using the solvent as the internal reference; coupling constants are given in Hz. The following abbreviation were used: s = singlet, d = doublet, t = triplet, q = quartet, dd = double doublet, ddd = double double doublet, bs = broad singlet, and m = multiplet. Mass spectra were recorded on a ThermoFinnigan LCQ Classic LC/MS/MS ion trap equipped with an ESI source and a syringe pump; samples (10^–4^–10^–5^ M in MeOH/H_2_O 80:20) were infused in the electrospray system at a flow rate of 5–10 μL min^–1^; when necessary, 50 μL of 10^–2^ M HCOOH or 10^–2^ M NH_3_ was added to the sample solutions to promote the analyte ionization; the ESI-MS data are given as *m*/*z*, with mass expressed in amu. Melting points were determined on a FALC Mod. 360 D apparatus and are uncorrected. Infrared spectra were recorded on a Perkin Elmer Spectrum One FT-IR spectrometer equipped with an ATR system. The purity of the compounds was determined by elemental analyses obtained by a PE 2400 (Perkin-Elmer) analyzer, and the analytical results were within ±0.4% of the theoretical values for all compounds.

### General Experimental Procedures

#### General Procedure A (GP-A) for the Synthesis of Pyrimidine Intermediates **1** and **2**

To a solution of *N*-Boc-1,5-diaminopentane (1 equiv) or *N*-Boc-1,6-diaminohexane (1 equiv) in 20 mL of MeOH, TEA (1 equiv) and 2-chloropyrimidine (1 equiv) were added. The reaction mixture was stirred at reflux for 20 h. Then it was cooled to room temperature and the solvent was removed under reduced pressure. The residue was diluted with a saturated aqueous solution of Na_2_CO_3_ (25 mL) and extracted with CH_2_Cl_2_ (3 × 25 mL). The combined organic layer was dried over Na_2_SO_4_ and filtered, and the solvent was evaporated under reduced pressure. The crude material was purified by column chromatography on a silica gel (CH_2_Cl_2_/MeOH 9.5:0.5). For each compound, *R*_f_, yield (%), ^1^H NMR, ESI-MS, and elemental analysis are reported.

#### General Procedure B (GP-B) for the Synthesis of *N*^1^-(Pyrimidin-2-yl)pentane-1,5-diamine (**3**), *N*^1^-(Pyrimidin-2-yl)hexane-1,6-diamine (**4**), *N*^1^-(Pyridin-2-yl)pentane-1,5-diamine (**7**), and *N*^1^-(Pyridin-2-yl)hexane-1,6-diamine (**8**)

##### Procedure 1

Intermediates **1**, **2**, **5**, or **6** (1 equiv), used for the synthesis of compounds **3**, **4**, **7**, and **8**, respectively, were dissolved in 20 mL of dry CH_2_Cl_2_, and to the solution, TFA (20 equiv) was added. The mixture was stirred at room temperature for 3.5 h, and then the solvent and the excess of TFA were removed under reduced pressure to give trifluoroacetic salts of compounds **3**, **4**, **7**, or **8** as yellow oils, which were used for the subsequent reactions without further purification.

##### Procedure 2

To a solution of intermediate **2** or **6** (1 equiv) in 20 mL of dry CH_2_Cl_2_, TFA (20 equiv) was added. The mixture was stirred at room temperature for 3.5 h, and then it was extracted twice with 5 N NaOH solution (2 × 20 mL) and once with H_2_O (20 mL). The organic layer was dried over Na_2_SO_4_ and filtered, and the solvent was removed under vacuum to give compounds **4** (77% yield) or **8** (88% yield) as yellow oils, which were used for the subsequent reactions without further purification.

##### Procedure 3

To a solution of 1,6-diaminohexane (1.516 g, 13.05 mmol) in 10 mL of MeOH, TEA (605 μL, *d* = 0.726 g/mL, 4.35 mmol) and 2-chloropyrimidine (0.498 g, 4.35 mmol) were added. The reaction mixture was stirred at reflux for 20 h. Then it was cooled to room temperature, and the solvent was removed under reduced pressure. The residue was diluted with a saturated aqueous solution of Na_2_CO_3_ (15 mL) and extracted with CH_2_Cl_2_ (3 × 20 mL). The combined organic layer was dried over Na_2_SO_4_, filtered, and concentrated under reduced pressure. The crude material was purified by column chromatography on a silica gel (MeOH/CH_2_Cl_2_/TEA 8:2:0.2, *R*_f_ = 0.17), and subsequently, the product was filtered with warm acetonitrile to afford compound **4**.

#### General Procedure C (GP-C) for the Synthesis of Pyridine Intermediates **5** and **6**

An oven-dried Schlenk flask equipped with a Teflon valve was charged with *N*-Boc-1,5-diaminopentane or *N*-Boc-1,6-diaminohexane (1.5 equiv), CuI (0.05 equiv), Cs_2_CO_3_ (2 equiv), and a magnetic stir bar. The flask was closed, evacuated, and backfilled with N_2_ for at least 10 min. Under a counter flow of N_2_, DMF (0.5 mL), 2-iodopyridine (1 equiv), and finally 2-isobutyrylcyclohexanone (0.2 equiv) were added by a syringe. The mixture was allowed to stir under N_2_ at 40 °C for 21 h. After this period, the mixture was diluted with ethyl acetate, transferred to a centrifuge tube, and centrifuged to separate the inorganic salts. The solvent was removed under reduced pressure. The crude residue was purified by column chromatography on a silica gel (CH_2_Cl_2_/MeOH 9.5:0.5). For each compound, *R*_f_, yield (%), ^1^H NMR, ESI-MS, and elemental analysis are reported.

#### General Procedures D (GP-D) for the Synthesis of Pyrimidine and Pyridine Diamine Derivatives **9**–**33**

##### Procedure 1

Intermediate **3**, **4**, **7**, or **8** (1 equiv), obtained following procedure 1 of GP-B described above, was dissolved in 20 mL of dry CH_2_Cl_2_, and to the solution, the opportune aldehyde (1 equiv) and dry K_2_CO_3_ (20 equiv) were added. The mixture was stirred at room temperature for 12 h and then filtered. The solvent was removed under reduced pressure, and the residue was dissolved in 30 mL of MeOH and treated with NaBH_4_ (3 equiv) at room temperature for 2 h. Then 5 mL of H_2_O was added; after 5 min under stirring, MeOH was evaporated under reduced pressure, H_2_O (20 mL) was added, and the mixture was extracted with CH_2_Cl_2_ (3 × 20 mL). The organic layer was dried over Na_2_SO_4_, and the solvent was removed under vacuum. The obtained residue was purified by column chromatography on a silica gel. For each compound chromatography system, *R*_f_, yield (%), ^1^H NMR, ^13^C NMR, ESI-MS, IR, melting point (°C), and elemental analysis are reported.

##### Procedure 2

Intermediate **4** (1 equiv), obtained following procedure 2 of GP-B described above, was dissolved in 20 mL of dry CH_2_Cl_2_, and to the solution, the opportune aldehyde (1 equiv) and molecular sieves (4 Å) were added. The mixture was stirred at room temperature for 12 h and then filtered. The solvent was removed under reduced pressure, and the residue was dissolved in 30 mL of MeOH and treated with NaBH_4_ (3 equiv) at room temperature for 2 h. Then 5 mL of H_2_O was added; after 5 min under stirring, MeOH was evaporated under reduced pressure, H_2_O (20 mL) was added, and the mixture was extracted with CH_2_Cl_2_ (3 × 20 mL). The organic layer was dried over Na_2_SO_4_, and the solvent was removed under vacuum. The obtained residue was purified by column chromatography on a silica gel. For each compound chromatography system, *R*_f_, yield (%), ^1^H NMR, ^13^C NMR, ESI-MS, IR, melting point (°C), and elemental analysis are reported.

##### Procedure 3

Intermediate **4** or **8** (1 equiv), obtained following, respectively, procedure 3 or 2 of GP-B described above, was dissolved in 20 mL of dry CH_2_Cl_2_, and to the solution, the opportune aldehyde (1 equiv) and molecular sieves (4 Å) were added. The mixture was stirred at room temperature for 12 h and then filtered. The solvent was removed under reduced pressure, and the residue was dissolved in 30 mL of MeOH and treated with NaBH_4_ (3 equiv) at room temperature for 2 h. Then 5 mL of 1 M HCl was added; after 5 min under stirring, the solvent was evaporated under reduced pressure, and the residue was diluted with 20 mL of saturated aqueous solution of NaHCO_3_ and extracted with CH_2_Cl_2_ (3 × 20 mL). The organic layer was dried over Na_2_SO_4_, and the solvent was removed under vacuum. The obtained residue was purified by column chromatography on a silica gel and/or by crystallization. For each compound chromatography system (and/or crystallization solvent), *R*_f_, yield (%), ^1^H NMR, ^13^C NMR, ESI-MS, IR, melting point (°C), and elemental analysis are reported.

##### *tert*-Butyl {5**-[(**Pyrimidin-2-yl)amino]pentyl}carbamate (**1**)

Compound **1** was prepared using *N*-Boc-1,5-diaminopentane (0.773 g, 3.82 mmol), TEA (531 μL, *d* = 0.726 g/mL, 3.82 mmol), and 2-chloropyrimidine (0.438 g, 3.82 mmol) following GP-A. *R*_f_ (CH_2_Cl_2_/MeOH 9.5:0.5) = 0.34. White solid, 0.539 g, 50% yield. ^1^H NMR (400 MHz) (CD_3_CN) δ (ppm): 8.22 (d, 2H, *J* = 4.8 Hz, pyrimidine); 6.51 (t, 1H, *J* = 4.8 Hz, pyrimidine); 5.68 (bs, 1H, pyrimidine-NH-); 5.26 (bs, 1H, Boc-NH-); 3.32 (q, 2H, *J* = 6.9 Hz, pyrimidine-NH-CH_2_-); 3.00 (q, 2H, *J* = 6.6 Hz, Boc-NH-CH_2_-); 1.60–1.53 (m, 2H, pyrimidine-NH-CH_2_-CH_2_-); 1.49–1.30 (m, 13H, pyrimidine-NH-CH_2_-CH_2_-CH_2_-CH_2_-CH_2_-NH-COO-C(CH_3_)_3_). ESI-MS (*m*/*z*): [M + H]^+^ = 280.79. Anal. (C_14_H_24_N_4_) C, H, N calcd: C 59.98%, H 8.63%, N 19.98%; found: C 59.89%, H 8.59%, N 19.90%.

##### *tert*-Butyl {6-[(Pyrimidin-2-yl)amino]hexyl}carbamate (**2**)

Compound **2** was prepared using *N*-Boc-1,6-diaminohexane (0.437 g, 2.02 mmol), TEA (281 μL, *d* = 0.726 g/mL, 2.02 mmol), and 2-chloropyrimidine (0.231 g, 2.02 mmol) following GP-A. *R*_f_ (CH_2_Cl_2_/MeOH 9.5:0.5) = 0.5. White solid, 0.358 g, 60% yield. ^1^H NMR (400 MHz) (CD_3_CN) δ (ppm): 8.22 (d, 2H, *J* = 4.4 Hz, pyrimidine); 6.51 (t, 1H, *J* = 4.2 Hz, pyrimidine); 5.69 (bs, 1H, pyrimidine-NH-); 5.24 (bs, 1H, Boc-NH-); 3.32 (q, 2H, *J* = 6.5 Hz, pyrimidine-NH-CH_2_-); 3.00 (q, 2H, *J* = 6.2 Hz, Boc-NH-CH_2_-); 1.59–1.52 (m, 2H, pyrimidine-NH-CH_2_-CH_2_-); 1.46–1.23 (m, 15H, pyrimidine-NH-CH_2_-CH_2_-CH_2_-CH_2_-CH_2_-CH_2_-NH-COO-C(CH_3_)_3_). ESI-MS (*m*/*z*): [M + H]^+^ = 294.73. Anal. (C_15_H_26_N_4_) C, H, N calcd: C 61.20%, H 8.90%, N 19.03%; found: C 60.97%, H 8.86%, N 18.96%.

##### *N*^1^-(Pyrimidin-2-yl)pentane-1,5-diamine (**3**)

Compound **3** was prepared following procedure 1 of GP-B as trifluoroacetic salt and used for the subsequent reactions without purification.

##### *N*^1^-(Pyrimidin-2-yl)hexane-1,6-diamine (**4**)

Compound **4** was prepared following procedure 1 of GP-B as trifluoroacetic salt (used for the subsequent reactions without purification) or following procedure 2 or 3 of GP-B as free amine. Yellow wax, 0.590 g, 70% yield. ^1^H NMR (400 MHz) (MeOD) δ (ppm): 8.24 (d, 2H, *J* = 4.8 Hz, pyrimidine); 6.57 (t, 1H, *J* = 4.9 Hz, pyrimidine); 3.35 (t, 2H, *J* = 7.1 Hz, pyrimidine-NH-CH_2_-); 2.71 (t, 2H, *J* = 7.2 Hz; -CH_2_-NH_2_); 1.66–1.40 (m, 8H, pyrimidine-NH-CH_2_-CH_2_-CH_2_-CH_2_-CH_2_-CH_2_-NH_2_). ESI-MS (*m*/*z*): [M + H]^+^ = 194.70. Anal. (C_10_H_18_N_4_) C, H, N calcd: C 61.82%, H 9.34%, N 28.84%; found: C 61.58%, H 9.30%, N 28.73%.

##### *tert*-Butyl {5-[(Pyridin-2-yl)amino]pentyl}carbamate (**5**)

Compound **5** was prepared using *N*-Boc-1,5-diaminopentane (0.303 g, 1.5 mmol), CuI (0.095 g, 0.05 mmol), Cs_2_CO_3_ (0.652 g, 2 mmol), DMF (0.5 mL), 2-iodopyridine (107 μL, *d* = 1.928 g/mL, 1 mmol), and 2-isobutyrylcyclohexanone (33 μL, *d* = 1.008 g/mL, 0.2 mmol) following GP-C. *R*_f_ (CH_2_Cl_2_/MeOH 9.5:0.5) = 0.38. Yellowish solid, 0.279 g, 100% yield. ^1^H NMR (400 MHz) (CD_3_CN) δ (ppm): 7.97 (d, 1H, *J* = 4.1 Hz, pyridine); 7.39–7.35 (m, 1H, pyridine); 6.50–6.47 (m, 1H, pyridine); 6.41 (d, 1H, *J* = 8.4 Hz, pyridine); 5.26 (bs, 1H, Boc-NH-); 5.09 (bs, 1H, pyridine-NH-); 3.23 (q, 2H, *J* = 6.9 Hz, pyridine-NH-CH_2_-); 3.01 (q, 2H, *J* = 6.6 Hz, Boc-NH-CH_2_-); 1.60–1.53 (m, 2H, pyridine-NH-CH_2_-CH_2_-); 1.50–1.31 (m, 13H, pyridine-NH-CH_2_-CH_2_-CH_2_-CH_2_-CH_2_-NH-COO-C(CH_3_)_3_). ESI-MS (*m*/*z*): [M + H]^+^ = 280.00. Anal. (C_15_H_25_N_3_) C, H, N calcd: C 64.49%, H 9.02%, N 15.04%; found: C 64.25%, H 8.98%, N 14.98%.

##### *tert*-Butyl {6-[(Pyridin-2-yl)amino]hexyl}carbamate (**6**)

Compound **6** was prepared using *N*-Boc-1,6-diaminohexane (0.811 g, 3.75 mmol), CuI (0.024 g, 0.125 mmol), Cs_2_CO_3_ (1.63 g, 5 mmol), DMF (1.25 mL), 2-iodopyridine (267 μL, *d* = 1.928 g/mL, 2.5 mmol), and 2-isobutyrylcyclohexanone (83 μL, *d* = 1.008 g/mL, 0.5 mmol) following GP-C. *R*_f_ (CH_2_Cl_2_/MeOH 9.5:0.5) = 0.38. Yellowish solid, 0.734 g, 100% yield. ^1^H NMR (400 MHz) (CD_3_CN) δ (ppm): 7.97 (dd, 1H, *J_1_* = 5.0 Hz, *J_2_* = 1.1 Hz, pyridine); 7.39–7.35 (m, 1H, pyridine); 6.50–6.47 (m, 1H, pyridine); 6.40 (d, 1H, *J* = 8.4 Hz, pyridine); 5.25 (bs, 1H, Boc-NH-); 5.09 (bs, 1H, pyridine-NH-); 3.23 (q, 2H, *J* = 7.0 Hz, pyridine-NH-CH_2_-); 3.00 (q, 2H, *J* = 6.6 Hz, Boc-NH-CH_2_-); 1.59–1.52 (m, 2H, pyridine-NH-CH_2_-CH_2_-); 1.46–1.27 (m, 15H, pyridine-NH-CH_2_-CH_2_-CH_2_-CH_2_-CH_2_-CH_2_-NH-COO-C(CH_3_)_3_). ESI-MS (*m*/*z*): [M + H]^+^ = 293.87. Anal. (C_16_H_27_N_3_) C, H, N calcd: C 65.50%, H 9.28%, N 14.32%; found: C 65.24%, H 9.24%, N 14.26%.

##### *N*^1^-(Pyridin-2-yl)pentane-1,5-diamine (**7**)

Compound **7** was prepared following procedure 1 of GP-B as trifluoroacetic salt and used for the subsequent reactions without purification.

##### *N*^1^-(Pyridin-2-yl)hexane-1,6-diamine (**8**)

Compound **8** was prepared following procedure 1 of GP-B as trifluoroacetic salt or following procedure 2 of GP-B as free amine and used for the subsequent reactions without purification.

##### *N*^1^-Benzyl-*N*^5^-(pyrimidin-2-yl)pentane-1,5-diamine (**9**)

Compound **9** was prepared using *N*^1^-(pyrimidin-2-yl)pentane-1,5-diamine (**3**) (0.108 g, 0.60 mmol), benzaldehyde (61 μL, *d* = 1.045 g/mL, 0.60 mmol), K_2_CO_3_ (1.659 g, 12.0 mmol), and NaBH_4_ (0.068 g, 1.8 mmol) following procedure 1 of GP-D described above. Column chromatography: silica gel, CH_2_Cl_2_/MeOH 1:1, *R*_f_ = 0.4. White solid, 0.093 g, 57% yield. ^1^H NMR (400 MHz) (CD_3_CN) δ (ppm): 8.22 (d, 2H, *J* = 4.7 Hz, pyrimidine); 7.31–7.20 (m, 5H, aromatic); 6.50 (t, 1H, *J* = 4.8 Hz, pyrimidine); 5.69 (bs, 1H, pyrimidine-NH-); 3.71 (s, 2H, Ar-CH_2_-NH-); 3.32 (q, 2H, *J* = 6.9 Hz, pyrimidine-NH-CH_2_-); 2.54 (t, 2H, *J* = 6.9 Hz, Ar-CH_2_-NH-CH_2_-); 1.59–1.45 (m, 4H, -NH-CH_2_-CH_2_-CH_2_-CH_2_-CH_2_-NH-); 1.41–1.34 (m, 2H, -NH-CH_2_-CH_2_-CH_2_-CH_2_-CH_2_-NH-). ^13^C NMR (100 MHz) (CD_3_CN) δ (ppm): 163.7; 158.9; 142.4; 129.1; 128.9; 127.5; 111.0; 54.3; 49.9; 41.8; 30.5; 30.1; 25.4. ESI-MS (*m*/*z*): [M + H]^+^ = 270.87. I.R. (cm^–1^): 3255; 1124; 1100; 1074; 1027. m.p. = 58.0–59.8 °C. Anal. (C_16_H_22_N_4_) C, H, N calcd: C 71.08%, H 8.20%, N 20.72%; found: C 70.98%, H 8.23%, N 20.79%.

##### *N*^1^-(4-Bromobenzyl)-*N*^5^-(pyrimidin-2-yl)pentane-1,5-diamine (**10**)

Compound **10** was prepared using *N*^1^-(pyrimidin-2-yl)pentane-1,5-diamine (**3**) (0.097 g, 0.54 mmol), 4-bromobenzaldehyde (0.100 g, 0.54 mmol), K_2_CO_3_ (1.493 g, 10.8 mmol), and NaBH_4_ (0.061 g, 1.62 mmol) following procedure 1 of GP-D described above. Column chromatography: silica gel, CH_2_Cl_2_/MeOH 1:1, *R*_f_ = 0.5. White solid, 0.093 g, 50% yield. ^1^H NMR (400 MHz) (CD_3_CN) δ (ppm): 8.22 (d, 2H, *J* = 4.8 Hz, pyrimidine); 7.45 (m, 2H, aromatic); 7.25 (m, 2H, aromatic); 6.51 (t, 1H, *J* = 4.8 Hz, pyrimidine); 5.68 (bs, 1H, pyrimidine-NH-); 3.68 (s, 2H, Ar-CH_2_-NH-); 3.32 (q, 2H, *J* = 6.9 Hz, pyrimidine-NH-CH_2_-); 2.52 (t, 2H, *J* = 6.8 Hz, Ar-CH_2_-NH-CH_2_-); 1.59–1.44 (m, 4H, -NH-CH_2_-CH_2_-CH_2_-CH_2_-CH_2_-NH-); 1.41–1.33 (m, 2H, -NH-CH_2_-CH_2_-CH_2_-CH_2_-CH_2_-NH-). ^13^C NMR (100 MHz) (MeOD) δ (ppm): 163.5; 159.2; 140.0; 132.5; 131.5; 121.8; 111.0; 53.7; 50.0; 42.0; 30.3; 30.1; 25.7. ESI-MS (*m*/*z*): [M + H]^+^ = 348.80 (95); 350.80 (100). I.R. (cm^–1^): 3235; 1265; 1129; 1066; 1007. m.p. = 75.2–77.0 °C. Anal. (C_16_H_21_BrN_4_) C, H, N calcd: C 55.02%, H 6.06%, N 16.04%; found: C 54.95%, H 6.08%, N 16.09%.

##### *N*^1^-(2-Methoxybenzyl)-*N*^5^-(pyrimidin-2-yl)pentane-1,5-diamine (**11**)

Compound **11** was prepared using *N*^1^-(pyrimidin-2-yl)pentane-1,5-diamine (**3**) (0.108 g, 0.60 mmol), 2-methoxybenzaldehyde (0.082 g, 0.60 mmol), K_2_CO_3_ (1.659 g, 12.0 mmol), and NaBH_4_ (0.068 g, 1.8 mmol) following procedure 1 of GP-D described above. Column chromatography: silica gel, CH_2_Cl_2_/MeOH 1:1, *R*_f_ = 0.3. Pale yellow solid, 0.091 g, 50% yield. ^1^H NMR (400 MHz) (CD_3_CN) δ (ppm): 8.22 (d, 2H, *J* = 4.8 Hz, pyrimidine); 7.26–7.20 (m, 2H, aromatic); 6.94–6.87 (m, 2H, aromatic); 6.50 (t, 1H, *J* = 4.8 Hz, pyrimidine); 5.68 (bs, 1H, pyrimidine-NH-); 3.80 (s, 3H, -O-CH_3_); 3.68 (s, 2H, Ar-CH_2_-NH-); 3.32 (q, 2H, *J* = 6.9 Hz, pyrimidine-NH-CH_2_-); 2.53 (t, 2H, *J* = 6.9 Hz, Ar-CH_2_-NH-CH_2_-); 1.59–1.45 (m, 4H, -NH-CH_2_-CH_2_-CH_2_-CH_2_-CH_2_-NH-); 1.40–1.33 (m, 2H, -NH-CH_2_-CH_2_-CH_2_-CH_2_-CH_2_-NH-). ^13^C NMR (100 MHz) (MeOD) δ (ppm): 163.5; 159.23; 159.19; 131.2; 129.8; 128.2; 121.5; 111.5; 111.0; 55.8; 49.8; 49.6; 42.0; 30.3; 30.0; 25.7. ESI-MS (*m*/*z*): [M + H]^+^ = 301.02. I.R. (cm^–1^): 3247; 1293; 1266; 1235; 1100; 1050; 1023; 932. m.p. = 44.8–46.8 °C. Anal. (C_17_H_24_N_4_O) C, H, N calcd: C 67.97%, H 8.05%, N 18.65%; found: C 67.98%, H 8.07%, N 18.60%.

##### *N*^1^-((2,6-Dichloropyridin-4-yl)methyl)-*N*^5^-(pyrimidin-2-yl)pentane-1,5-diamine (**12**)

Compound **12** was prepared using *N*^1^-(pyrimidin-2-yl)pentane-1,5-diamine (**3**) (0.090 g, 0.50 mmol), 2,6-dichloropyridine-4-carbaldehyde (0.088 g, 0.50 mmol), K_2_CO_3_ (1.382 g, 10.0 mmol), and NaBH_4_ (0.057 g, 1.5 mmol) following procedure 1 of GP-D described above. Column chromatography: silica gel, AcOEt/MeOH 1:1, *R*_f_ = 0.5. Pale yellow solid, 0.094 g, 55% yield. ^1^H NMR (400 MHz) (CD_3_CN) δ (ppm): 8.22 (d, 2H, *J* = 4.7 Hz, pyrimidine); 7.36 (s, 2H, pyridine); 6.50 (t, 1H, *J* = 4.8 Hz, pyrimidine); 5.67 (bs, 1H, pyrimidine-NH-); 3.75 (s, 2H, pyridine-CH_2_-NH-); 3.32 (q, 2H, *J* = 6.9 Hz, pyrimidine-NH-CH_2_-); 2.52 (t, 2H, *J* = 6.8 Hz, pyridine-CH_2_-NH-CH_2_-); 1.60–1.45 (m, 4H, -NH-CH_2_-CH_2_-CH_2_-CH_2_-CH_2_-NH-); 1.42–1.34 (m, 2H -NH-CH_2_-CH_2_-CH_2_-CH_2_-CH_2_-NH-). ^13^C NMR (100 MHz) (MeOD) δ (ppm): 163.5; 159.2; 157.7; 151.4; 123.6; 111.0; 52.4; 50.1; 42.1; 30.5; 30.6; 25.9. ESI-MS (*m*/*z*): [M + H]^+^ = 340.09 (100); 342.08 (65); 344.06 (10). Anal. (C_15_H_19_Cl_2_N_5_) C, H, N calcd: C 52.95%, H 5.63%, N 20.58%; found: C 52.91%, H 5.62%, N 20.61%.

##### *N*^1^-Benzyl-*N*^6^-(pyrimidin-2-yl)hexane-1,6-diamine (**13**)

Compound **13** was prepared using *N*^1^-(pyrimidin-2-yl)hexane-1,6-diamine (**4**) (0.103 g, 0.53 mmol), benzaldehyde (54 μL, *d* = 1.045 g/mL, 0.53 mmol), K_2_CO_3_ (1.465 g, 10.6 mmol), and NaBH_4_ (0.060 g, 1.59 mmol) following procedure 1 of GP-D described above. Column chromatography: silica gel, CH_2_Cl_2_/MeOH 7:3, *R*_f_ = 0.42. White solid, 0.066 g, 43% yield. ^1^H NMR (400 MHz) (CD_3_CN) δ (ppm): 8.22 (d, 2H, *J* = 4.8 Hz, pyrimidine); 7.33–7.20 (m, 5H, aromatic); 6.50 (t, 1H, *J* = 4.8 Hz, pyrimidine); 5.74 (bs, 1H, pyrimidine-NH-); 3.71 (s, 2H, Ar-CH_2_-NH-); 3.31 (q, 2H, *J* = 6.8 Hz, pyrimidine-NH-CH_2_-); 2.53 (t, 2H, *J* = 7.0 Hz, Ar-CH_2_-NH-CH_2_-); 1.59–1.52 (m, 2H, -NH-CH_2_-CH_2_-); 1.49–1.41 (m, 2H, -NH-CH_2_-CH_2_-); 1.38–1.27 (m, 4H -NH-CH_2_-CH_2_-CH_2_-CH_2_-CH_2_-CH_2_-NH-). ^13^C NMR (100 MHz) (CD_3_CN) δ (ppm): 163.7; 158.9; 142.2; 129.1; 129.0; 127.5; 111.0; 54.3; 49.9; 41.8; 30.6; 30.2; 27.8; 27.5. ESI-MS (*m*/*z*): [M + H]^+^ = 285.13. I.R. (cm^–1^): 3301; 3267; 1323; 1258; 1129; 1027; 984; 975. m.p. = 51.8–54.4 °C. Anal. (C_17_H_24_N_4_) C, H, N calcd: C 71.79%, H 8.51%, N 19.70%; found: C 71.89%, H 8.47%, N 19.64%.

##### *N*^1^-(4-Bromobenzyl)-*N*^6^-(pyrimidin-2-yl)hexane-1,6-diamine (**14**)

Compound **14** was prepared using *N*^1^-(pyrimidin-2-yl)hexane-1,6-diamine (**4**) (0.136 g, 0.70 mmol), 4-bromobenzaldehyde (0.130 g, 0.70 mmol), K_2_CO_3_ (1.935 g, 14.0 mmol), and NaBH_4_ (0.079 g, 2.1 mmol) following procedure 1 of GP-D described above. Column chromatography: silica gel, CH_2_Cl_2_/MeOH 1:1, *R*_f_ = 0.4. White solid, 0.152 g, 60% yield. ^1^H NMR (400 MHz) (CD_3_CN) δ (ppm): 8.21 (d, 2H, *J* = 4.7 Hz, pyrimidine); 7.45 (m, 2H, aromatic); 7.24 (m, 2H, aromatic); 6.50 (t, 1H, *J* = 4.8 Hz, pyrimidine); 5.77 (bs, 1H, pyrimidine-NH-); 3.67 (s, 2H, Ar-CH_2_-NH-); 3.31 (q, 2H, *J* = 6.7 Hz, pyrimidine-NH-CH_2_-); 2.50 (t, 2H, *J* = 6.9 Hz, Ar-CH_2_-NH-CH_2_-); 1.58–1.51 (m, 2H, -NH-CH_2_-CH_2_-); 1.47–1.41 (m, 2H, -NH-CH_2_-CH_2_-); 1.37–1.26 (m, 4H -NH-CH_2_-CH_2_-CH_2_-CH_2_-CH_2_-CH_2_-NH-). ^13^C NMR (100 MHz) (CD_3_CN) δ (ppm): 163.7; 158.9; 141.8; 132.0; 130.9; 120.6; 111.0; 53.5; 49.8; 41.8; 30.7; 30.2; 27.7; 27.5. ESI-MS (*m*/*z*): [M + H]^+^ = 363.07 (95); 365.00 (100). I.R. (cm^–1^): 3267; 1255; 1132; 1068; 1010. m.p. = 43–46 °C. Anal. (C_17_H_23_BrN_4_) C, H, N calcd: C 56.20%, H 6.38%, N 15.42%; found: C 56.21%, H 6.39%, N 15.45%.

##### *N*^1^-(4-Chlorobenzyl)-*N*^6^-(pyrimidin-2-yl)hexane-1,6-diamine (**15**)

Compound **15** was prepared using *N*^1^-(pyrimidin-2-yl)hexane-1,6-diamine (**4**) (0.107 g, 0.55 mmol), 4-chlorobenzaldehyde (0.077 g, 0.55 mmol), K_2_CO_3_ (1.520 g, 11.0 mmol), and NaBH_4_ (0.062 g, 1.65 mmol) following procedure 1 of GP-D described above. Column chromatography: silica gel, CH_2_Cl_2_/MeOH 7:3, *R*_f_ = 0.53. White solid, 0.085 g, 50% yield. ^1^H NMR (400 MHz) (CD_3_CN) δ (ppm): 8.22 (d, 2H, *J* = 4.8 Hz, pyrimidine); 7.31 (s, 4H, aromatic); 6.50 (t, 1H, *J* = 4.8 Hz, pyrimidine); 5.71 (bs, 1H, pyrimidine-NH-); 3.69 (s, 2H, Ar-CH_2_-NH-); 3.31 (q, 2H, *J* = 6.8 Hz, pyrimidine-NH-CH_2_-); 2.51 (t, 2H, *J* = 6.9 Hz, Ar-CH_2_-NH-CH_2_-); 1.58–1.51 (m, 2H, -NH-CH_2_-CH_2_-); 1.48–1.41 (m, 2H, -NH-CH_2_-CH_2_-); 1.38–1.27 (m, 4H -NH-CH_2_-CH_2_-CH_2_-CH_2_-CH_2_-CH_2_-NH-). ^13^C NMR (100 MHz) (CD_3_CN) δ (ppm): 163.7; 158.9; 141.3; 132.5; 130.6; 129.0; 111.0; 53.5; 49.8; 41.8; 30.6; 30.2; 27.7; 27.5. ESI-MS (*m*/*z*): [M + H]^+^ = 318.87 (100); 320.87 (30). I.R. (cm^–1^): 3264; 1256; 1132; 1123; 1088; 1012; 987. m.p. = 53–56 °C. Anal. (C_17_H_23_ClN_4_) C, H, N calcd: C 64.04%, H 7.27%, N 17.57%; found: C 64.08%, H 7.26%, N 17.52%.

##### *N*^1^-(2-Methoxybenzyl)-*N*^6^-(pyrimidin-2-yl)hexane-1,6-diamine (**16**)

Compound **16** was prepared using *N*^1^-(pyrimidin-2-yl)hexane-1,6-diamine (**4**) (0.185 g, 0.95 mmol), 2-methoxybenzaldehyde (0.129 g, 0.95 mmol), K_2_CO_3_ (2.626 g, 19.0 mmol), and NaBH_4_ (0.108 g, 2.85 mmol) following procedure 1 of GP-D described above. Column chromatography: silica gel, CH_2_Cl_2_/MeOH 1:1, *R*_f_ = 0.23. Pale yellow oil, 0.184 g, 62% yield. ^1^H NMR (400 MHz) (CD_3_CN) δ (ppm): 8.22 (d, 2H, *J* = 4.8 Hz, pyrimidine); 7.28–7.21 (m, 2H, aromatic); 6.94–6.88 (m, 2H, aromatic); 6.50 (t, 1H, *J* = 4.8 Hz, pyrimidine); 5.70 (bs, 1H, pyrimidine-NH-); 3.81 (s, 3H, -O-CH_3_); 3.71 (s, 2H, Ar-CH_2_-NH-); 3.32 (q, 2H, *J* = 6.8 Hz, pyrimidine-NH-CH_2_-); 2.54 (t, 2H, *J* = 7.0 Hz, Ar-CH_2_-NH-CH_2_-); 1.58–1.44 (m, 4H, -NH-CH_2_-CH_2_-CH_2_-CH_2_-CH_2_-CH_2_-NH-); 1.40–1.28 (m, 4H, -NH-CH_2_-CH_2_-CH_2_-CH_2_-CH_2_-CH_2_-). ^13^C NMR (100 MHz) (CD_3_CN) δ (ppm): 163.9; 159.0; 158.8; 130.5; 129.6; 129.1; 121.3; 111.6; 111.1; 56.1; 49.9; 49.3; 42.0; 30.6; 30.3; 27.9; 27.6. ESI-MS (*m*/*z*): [M + H]^+^ = 315.13. I.R. (cm^–1^): 3265; 1240; 1104; 1031. Anal. (C_18_H_26_N_4_O) C, H, N calcd: C 68.76%, H 8.33%, N 17.82%; found: C 68.70%, H 8.30%, N 17.78%.

##### *N*^1^-(Pyrimidin-2-yl)-*N*^6^-(3,4,5-trimethoxybenzyl)hexane-1,6-diamine (**17**)

Compound **17** was prepared using *N*^1^-(pyrimidin-2-yl)hexane-1,6-diamine (**4**) (0.101 g, 0.52 mmol), 3,4,5-trimethoxybenzaldehyde (0.102 g, 0.52 mmol), and NaBH_4_ (0.059 g, 1.56 mmol) following procedure 3 of GP-D described above. Column chromatography: silica gel, AcOEt/MeOH 1:1, *R*_f_ = 0.23. Yellow oil, 0.138 g, 71% yield. ^1^H NMR (400 MHz) (acetone-*d*_6_) δ (ppm): 8.22 (d, 2H, *J* = 4.7 Hz, pyrimidine); 6.67 (s, 2H, aromatic); 6.50 (t, 1H, *J* = 4.8 Hz, pyrimidine); 6.26 (bs, 1H, pyrimidine-NH-); 3.79 (s, 6H, Ar-(OCH_3_)_2_); 3.69 (s, 3H, Ar-OCH_3_); 3.68 (s, 2H, Ar-CH_2_-NH-); 3.40–3.35 (m, 2H, pyrimidine-NH-CH_2_-); 2.57 (t, 2H, *J* = 6.8 Hz, Ar-CH_2_-NH-CH_2_-); 1.64–1.57 (m, 2H, -NH-CH_2_-CH_2_-); 1.54–1.47 (m, 2H, -NH-CH_2_-CH_2_-); 1.43–1.37 (m, 4H, -NH-CH_2_-CH_2_-CH_2–_CH_2_-CH_2_-CH_2_-NH-). ^13^C NMR (100 MHz) (MeOD) δ (ppm): 163.5; 159.2; 154.5; 138.2; 136.3; 111.0; 106.9; 61.1; 56.6; 54.5; 49.8; 42.1; 30.4; 30.1; 28.2; 27.9. ESI-MS (*m*/*z*): [M + H]^+^ = 374.67. I.R. (cm^–1^): 3276; 1233; 1121; 1007. Anal. (C_20_H_30_N_4_O_3_) C, H, N calcd: C 64.15%, H 8.07%, N 14.96%; found: C 64.09%, H 8.09%, N 15.01%.

##### 2-Methoxy-4-(((6-(pyrimidin-2-ylamino)hexyl)amino)methyl)phenol (**18**)

Compound **18** was prepared using *N*^1^-(pyrimidin-2-yl)hexane-1,6-diamine (**4**) (0.117 g, 0.60 mmol), 4-hydroxy-3-methoxybenzaldehyde (0.091 g, 0.60 mmol), and NaBH_4_ (0.068 g, 1.80 mmol) following procedure 3 of GP-D described above. Column chromatography: silica gel, AcOEt/MeOH/TEA 5:5:0.1, *R*_f_ = 0.37. White solid, 0.135 g, 68% yield. ^1^H NMR (400 MHz) (CD_3_CN) δ (ppm): 8.21 (d, 2H, *J* = 4.8 Hz, pyrimidine); 6.91 (s, 1H, aromatic); 6.73 (s, 2H, aromatic); 6.50 (t, 1H, *J* = 4.8 Hz, pyrimidine); 5.72 (bs, 1H, pyrimidine-NH-); 3.80 (s, 3H, Ar-OCH_3_); 3.63 (s, 2H, Ar-CH_2_-NH-); 3.31 (q, 2H, *J* = 6.9 Hz, pyrimidine-NH-CH_2_-); 2.53 (t, 2H, *J* = 7.0 Hz, Ar-CH_2_-NH-CH_2_-); 1.58–1.51 (m, 2H, -NH-CH_2_-CH_2_-); 1.49–1.42 (m, 2H, -NH-CH_2_-CH_2_-); 1.39–1.32 (m, 4H, -NH-CH_2_-CH_2_-CH_2_-CH_2_-CH_2_-CH_2_-NH-). ^13^C NMR (100 MHz) (MeOD) δ (ppm): 163.5; 159.2; 149.2; 147.9; 128.2; 123.1; 116.3; 113.7; 111.0; 56.4; 53.4; 49.0; 42.0; 30.3; 28.8; 27.8; 27.7. ESI-MS (*m*/*z*): [M + H]^+^ = 330.60. I.R. (cm^–1^): 3261; 1284; 1258; 1158; 1130; 1034. m.p. = 87–89 °C. Anal. (C_18_H_26_N_4_O_2_) C, H, N calcd: C 65.43%, H 7.93%, N 16.96%; found: C 65.41%, H 7.92%, N 17.00%.

##### 3-(((6-(Pyrimidin-2-ylamino)hexyl)amino)methyl)phenol (**19**)

Compound **19** was prepared using *N*^1^-(pyrimidin-2-yl)hexane-1,6-diamine (**4**) (0.117 g, 0.60 mmol), 3-hydroxybenzaldehyde (0.073 g, 0.60 mmol), and NaBH_4_ (0.068 g, 1.80 mmol) following procedure 3 of GP-D described above. In this case, the extraction was carried out with AcOEt instead of CH_2_Cl_2_. Column chromatography: silica gel, initially CH_2_Cl_2_/MeOH 1:1 (until the separation of the spot with *R*_f_ = 0.9) and subsequently CH_2_Cl_2_/MeOH/TEA 5:5:0.1, *R*_f_ = 0.67. After the chromatography, the compound was purified by crystallization in CH_3_CN. Pinkish solid, 0.104 g, 57% yield. ^1^H NMR (400 MHz) (acetone-*d*_6_) δ (ppm): 8.22 (d, 2H, *J* = 4.6 Hz, pyrimidine); 7.09 (t, 1H, *J* = 7.8 Hz, aromatic); 6.85 (s, 1H, aromatic); 6.79 (d, 1H, *J* = 7.5 Hz, aromatic); 6.67 (dd, 1H, *J_1_* = 8.0 Hz, *J_2_* = 1.8 Hz, aromatic); 6.50 (t, 1H, *J* = 4.8 Hz, pyrimidine); 6.22 (bs, 1H, pyrimidine-NH-); 3.67 (s, 2H, Ar-CH_2_-NH-); 3.40–3.36 (m, 2H, pyrimidine-NH-CH_2_-); 2.56 (t, 2H, 6.88 Hz, Ar-CH_2_-NH-CH_2_-); 1.64–1.57 (m, 2H, -NH-CH_2_-CH_2_-); 1.53–1.45 (m, 2H, -NH-CH_2_-CH_2_-); 1.43–1.37 (m, 4H, -NH-CH_2_-CH_2_-CH_2_-CH_2_-CH_2_-CH_2_-NH-). ^13^C NMR (100 MHz) (MeOD) δ (ppm): 163.5; 159.2; 158.8; 141.8; 130.5; 120.5; 116.4; 115.2; 111.0; 54.3; 49.7; 42.1; 30.4; 30.2; 28.1; 27.8. ESI-MS (*m*/*z*): [M + H]^+^ = 301.02. I.R. (cm^–1^): 3289; 1287; 1239; 1228; 1160; 927. m.p. = 75–77 °C. Anal. (C_17_H_24_N_4_O) C, H, N calcd: C 67.97%, H 8.05%, N 18.65%; found: C 68.03%, H 8.04%, N 18.61%.

##### 3-(((6-(Pyrimidin-2-ylamino)hexyl)amino)methyl)benzene-**1,2-**diol (**20**)

Compound **20** was prepared using *N*^1^-(pyrimidin-2-yl)hexane-1,6-diamine (**4**) (0.152 g, 0.78 mmol), 2,3-dihydroxybenzaldehyde (0.108 g, 0.78 mmol), and NaBH_4_ (0.089 g, 2.34 mmol) following procedure 3 of GP-D described above. The compound was purified by crystallization in CH_3_CN. Pinkish solid, 0.145 g, 59% yield. ^1^H NMR (400 MHz) (*d*_6_-DMSO) δ (ppm): 8.22 (d, 2H, *J* = 4.7 Hz, pyrimidine); 7.08 (t, 1H, *J* = 5.6 Hz, pyrimidine-NH-); 6.61 (dd, 1H, *J_1_* = 7.5 Hz, *J_2_* = 1.9 Hz, aromatic); 6.52–6.46 (m, 3H, 2H aromatic and 1H pyrimidine); 3.80 (s, 2H, Ar-CH_2_-NH-); 3.24 (q, 2H, *J* = 6.5 Hz, pyrimidine-NH-CH_2_-); 1.53–1.41 (m, 4H, -NH-CH_2_-CH_2_-CH_2_-CH_2_-CH_2_-CH_2_-NH-); 1.31–1.29 (m, 4H, -NH-CH_2_-CH_2_-CH_2_-CH_2_-CH_2_-CH_2_-NH-). *Ar-CH_2_-NH-CH_2_- covered by *d*_6_-DMSO. ^13^C NMR (100 MHz) (MeOD) δ (ppm): 163.5; 159.2; 147.7; 146.9; 123.6; 121.1; 119.4; 115.6; 111.0; 51.4; 48.9; 42.0; 30.3; 29.5; 27.8; 27.6. ESI-MS (*m*/*z*): [M + H]^+^ = 316.87. I.R. (cm^–1^): 1289; 1194; 1102; 1068; 936. m.p. = 99–101 °C. Anal. (C_17_H_24_N_4_O_2_) C, H, N calcd: C 64.53%, H 7.65%, N 17.71%; found: C 64.56%, H 7.68%, N 17.67%.

##### *N*^1^-((2,6-Dichloropyridin-4-yl)methyl)-*N*^6^-(pyrimidin-2-yl)hexane-1,6-diamine (**21**)

Compound **21** was prepared using *N*^1^-(pyrimidin-2-yl)hexane-1,6-diamine (**4**) (0.117 g, 0.60 mmol), 2,6-dichloropyridine-4-carbaldehyde (0.106 g, 0.60 mmol), K_2_CO_3_ (1.659 g, 12.0 mmol), and NaBH_4_ (0.068 g, 1.8 mmol) following procedure 1 of GP-D described above. Column chromatography: silica gel, AcOEt/MeOH 9:1, *R*_f_ = 0.4. White solid, 0.119 g, 57% yield. ^1^H NMR (400 MHz) (CD_3_CN) δ (ppm): 8.21 (d, 2H, *J* = 4.8 Hz, pyrimidine); 7.36 (s, 2H, pyridine); 6.50 (t, 1H, *J* = 4.8 Hz, pyrimidine); 5.69 (bs, 1H, pyrimidine-NH-); 3.74 (s, 2H, pyridine-CH_2_-NH-); 3.32 (q, 2H, *J* = 6.8 Hz, pyrimidine-NH-CH_2_-); 2.51 (t, 2H, *J* = 6.9 Hz, pyridine-CH_2_-NH-CH_2_-); 1.59–1.52 (m, 2H, -NH-CH_2_-CH_2_-); 1.49–1.42 (m, 2H, -NH-CH_2_-CH_2_-); 1.39–1.33 (m, 4H, -NH-CH_2_-CH_2_-CH_2_-CH_2_-CH_2_-CH_2_-NH-). ^13^C NMR (100 MHz) (MeOD) δ (ppm): 163.5; 159.2; 157.8; 151.5; 123.6; 111.0; 52.4; 50.1; 42.1; 30.5; 30.4; 28.1; 27.9. ESI-MS (*m*/*z*): [M + H]^+^ = 353.98 (100); 355.91 (65); 356.97 (10). I.R. (cm^–1^): 3235; 1216; 1155; 1129; 987. m.p. = 68–70 °C. Anal. (C_16_H_21_Cl_2_N_5_) C, H, N calcd: C 54.24%, H 5.97%, N 19.77%; found: C 54.17%, H 5.95%, N 19.82%.

##### *N*^1^-((1*H*-Indol-3-yl)methyl)-*N*^6^-(pyrimidin-2-yl)hexane-1,6-diamine (**22**)

Compound **22** was prepared using *N*^1^-(pyrimidin-2-yl)hexane-1,6-diamine (**4**) (0.076 g, 0.39 mmol), 1*H*-indole-3-carbaldehyde (0.057 g, 0.39 mmol), and NaBH_4_ (0.044 g, 1.17 mmol) following procedure 2 of GP-D described above. Column chromatography: silica gel, initially MeOH (until the separation of the spot with *R*_f_ = 0.77) and subsequently MeOH/TEA 10:0.2, *R*_f_ = 0.20. After the chromatography, the compound was filtered with CH_2_Cl_2_ to remove silica. Orange oil, 0.044 g, 36% yield. ^1^H NMR (400 MHz) (CD_3_CN) δ (ppm): 9.18 (bs, 1H, indole-NH-); 8.21 (d, 2H, *J* = 4.8 Hz, pyrimidine); 7.62 (d, 1H, *J* = 7.9 Hz, indole); 7.38 (d, 1H, *J* = 8.1 Hz, indole); 7.15–7.10 (m, 2H, indole); 7.05–7.00 (m, 1H, indole); 6.50 (t, 1H, *J* = 4.8 Hz, pyrimidine); 5.70 (bs, 1H, pyrimidine-NH-); 3.89 (s, 2H, indole-CH_2_-NH-); 3.30 (q, 2H, *J* = 6.9 Hz, pyrimidine-NH-CH_2_-); 2.60 (t, 2H, *J* = 7.0 Hz, indole-CH_2_-NH-CH_2_-); 1.58–1.44 (m, 4H, -NH-CH_2_-CH_2_-CH_2_-CH_2_-CH_2_-CH_2_-NH-); 1.39–1.32 (m, 4H, -NH-CH_2_-CH_2_-CH_2_-CH_2_-CH_2_-CH_2_-NH-). ^13^C NMR (100 MHz) (CDCl_3_) δ (ppm): 162.5; 158.1; 136.4; 127.2; 123.4; 122.2; 119.7; 118.7; 113.5; 111.4; 110.4; 49.0; 44.3; 41.5; 29.5; 29.4; 27.1; 26.8. ESI-MS (*m*/*z*): [M + H]^+^ = 323.67. I.R. (cm^–1^): 3259; 1236; 1100; 1074; 1010; 983. Anal. (C_19_H_25_N_5_) C, H, N calcd: C 70.56%, H 7.79%, N 21.65%; found: C 70.47%, H 7.81%, N 21.72%.

##### *N*^1^-Benzyl-*N*^5^-(pyridin-2-yl)pentane-1,5-diamine (**23**)

Compound **23** was prepared using *N*^1^-(pyridin-2-yl)pentane-1,5-diamine (**7**) (0.086 g, 0.48 mmol), benzaldehyde (49 μL, *d* = 1.045 g/mL, 0.48 mmol), K_2_CO_3_ (1.327 g, 9.6 mmol), and NaBH_4_ (0.054 g, 1.44 mmol) following procedure 1 of GP-D described above. Column chromatography: silica gel, CH_2_Cl_2_/MeOH 1:1, *R*_f_ = 0.36. Yellow oil, 0.071 g, 55% yield. ^1^H NMR (400 MHz) (CD_3_CN) δ (ppm): 7.96 (dd, 1H, *J_1_* = 5.0 Hz, *J_2_* = 1.0 Hz, pyridine); 7.39–7.34 (m, 1H, pyridine); 7.33–7.20 (m, 5H, aromatic); 6.48 (ddd, 1H, *J_1_* = 7.0 Hz, *J_2_* = 5.0 Hz, *J_3_* = 0.8 Hz, pyridine); 6.40 (d, 1H, *J* = 8.4 Hz, pyridine); 5.09 (s, 1H, pyridine-NH-); 3.72 (s, 2H, Ar-CH_2_-NH-); 3.23 (q, 2H, *J* = 6.9 Hz, pyridine-NH-CH_2_-); 2.55 (t, 2H, *J* = 6.9 Hz, Ar-CH_2_-NH-CH_2_-); 1.59–1.46 (m, 4H, -NH-CH_2_-CH_2_-CH_2_-CH_2_-CH_2_-NH-); 1.43–1.35 (m, 2H, -NH-CH_2_-CH_2_-CH_2_-CH_2_-CH_2_-NH-). ^13^C NMR (100 MHz) (MeOD) δ (ppm): 160.4; 147.8; 140.4; 138.7; 129.6; 129.5; 128.2; 112.9; 109.7; 54.4; 49.8; 42.5; 30.3; 30.0; 25.8. ESI-MS (*m*/*z*): [M + H]^+^ = 270.00. I.R. (cm^–1^): 3286; 1289; 1152; 982. Anal. (C_17_H_23_N_3_) C, H, N calcd: C 75.80%, H 8.61%, N 15.60%; found: C 75.86%, H 8.59%, N 15.55%.

##### *N*^1^-(4-Bromobenzyl)-*N*^5^-(pyridin-2-yl)pentane-1,5-diamine (**24**)

Compound **24** was prepared using *N*^1^-(pyridin-2-yl)pentane-1,5-diamine (**7**) (0.075 g, 0.42 mmol), 4-bromobenzaldehyde (0.078 g, 0.42 mmol), K_2_CO_3_ (1.161 g, 8.4 mmol), and NaBH_4_ (0.048 g, 1.26 mmol) following procedure 1 of GP-D described above. Column chromatography: silica gel, AcOEt/MeOH 1:1, *R*_f_ = 0.33. White solid, 0.070 g, 48% yield. ^1^H NMR (400 MHz) (CD_3_CN) δ (ppm): 7.96 (dd, 1H, *J_1_* = 4.9 Hz, *J_2_* = 1.0 Hz, pyridine); 7.46 (m, 2H, aromatic); 7.39–7.34 (m, 1H, pyridine); 7.25 (m, 2H, aromatic); 6.50–6.47 (m, 1H, pyridine); 6.40 (d, 1H, *J* = 8.4 Hz, pyridine); 5.07 (bs, 1H, pyridine-NH-); 3.68 (s, 2H, Ar-CH_2_-NH-); 3.23 (q, 2H, *J* = 6.9 Hz, pyridine-NH-CH_2_-); 2.53 (t, 2H, *J* = 6.8 Hz, Ar-CH_2_-NH-CH_2_-); 1.59–1.35 (m, 6H, -NH- CH_2_-CH_2_-CH_2_-CH_2_-CH_2_-NH-). ^13^C NMR (100 MHz) (MeOD) δ (ppm): 160.4; 147.8; 140.0; 138.7; 132.5; 131.5; 121.8; 112.9; 109.7; 53.7; 49.8; 42.5; 30.3; 30.2; 25.8. ESI-MS (*m*/*z*): [M + H]^+^ = 347.54 (100); 349.60 (90). I.R. (cm^–1^): 3284; 1287; 1156; 1135; 1069; 1009; 980. m.p. = 65–68 °C. Anal. (C_17_H_22_BrN_3_) C, H, N calcd: C 58.63%, H 6.37%, N 12.07%; found: C 58.59%, H 6.36%, N 12.04%.

##### *N*^1^-Benzyl-*N*^6^-(pyridin-2-yl)hexane-1,6-diamine (**25**)

Compound **25** was prepared using *N*^1^-(pyridin-2-yl)hexane-1,6-diamine (**8**) (0.106 g, 0.55 mmol), benzaldehyde (56 μL, *d* = 1.045 g/mL, 0.55 mmol), K_2_CO_3_ (1.520 g, 11.0 mmol), and NaBH_4_ (0.062 g, 1.65 mmol) following procedure 1 of GP-D described above. Column chromatography: silica gel, CH_2_Cl_2_/MeOH 7:3, *R*_f_ = 0.3. Yellow oil, 0.083 g, 53% yield. ^1^H NMR (400 MHz) (CD_3_CN) δ (ppm): 7.96 (dd, 1H, *J_1_* = 5.0 Hz, *J_2_* = 1.1 Hz, pyridine); 7.39–7.34 (m, 1H, pyridine); 7.33–7.20 (m, 5H, aromatic); 6.48 (ddd, 1H, *J_1_* = 7.0 Hz, *J_2_* = 5.1 Hz, *J_3_* = 0.8 Hz, pyridine); 6.40 (d, 1H, *J* = 8.4 Hz, pyridine); 5.10 (bs, 1H, pyridine-NH-); 3.72 (s, 2H, Ar-CH_2_-NH-); 3.23 (q, 2H, *J* = 6.9 Hz, pyridine-NH-CH_2_-); 2.54 (t, 2H, *J* = 7.0 Hz, Ar-CH_2_-NH-CH_2_-); 1.59–1.52 (m, 2H, -NH-CH_2_-CH_2_-); 1.50–1.43 (m, 2H, -NH-CH_2_-CH_2_-); 1.40–1.27 (m, 4H, -NH-CH_2_-CH_2_-CH_2_-CH_2_-CH_2_-CH_2_-NH-). ^13^C NMR (100 MHz) (CD_3_CN) δ (ppm): 160.2; 148.8; 142.2; 137.8; 129.1; 129.0; 127.5; 112.8; 108.1; 54.3; 49.9; 42.2; 30.7; 30.2; 27.8; 27.6. ESI-MS (*m*/*z*): [M + H]^+^ = 283.98. I.R. (cm^–1^): 3279; 1288; 1152; 982. Anal. (C_18_H_25_N_3_) C, H, N calcd: C 76.28%, H 8.89%, N 14.83%; found: C 76.33%, H 8.87%, N 14.80%.

##### *N*^1^-(4-Bromobenzyl)-*N*^6^-(pyridin-2-yl)hexane-1,6-diamine (**26**)

Compound **26** was prepared using *N*^1^-(pyridin-2-yl)hexane-1,6-diamine (**8**) (0.110 g, 0.57 mmol), 4-bromobenzaldehyde (0.106 g, 0.57 mmol), K_2_CO_3_ (1.576 g, 11.4 mmol), and NaBH_4_ (0.065 g, 1.71 mmol) following procedure 1 of GP-D described above. Column chromatography: silica gel, CH_2_Cl_2_/MeOH 1:1, *R*_f_ = 0.38. Yellow solid, 0.084 g, 40% yield. ^1^H NMR (400 MHz) (CD_3_CN) δ (ppm): 7.96 (dd, 1H, *J_1_* = 5.0 Hz, *J_2_* = 1.1 Hz, pyridine); 7.46 (m, 2H, aromatic); 7.39–7.34 (m, 1H, pyridine); 7.25 (m, 2H, aromatic); 6.48 (ddd, 1H, *J_1_* = 7.0 Hz, *J_2_* = 5.0 Hz, *J_3_* = 0.8 Hz, pyridine); 6.40 (d, 1H, *J* = 8.4 Hz, pyridine); 5.08 (bs, 1H, pyridine-NH-); 3.68 (s, 2H, Ar-CH_2_-NH-); 3.22 (q, 2H, *J* = 6.9 Hz, pyridine-NH-CH_2_-); 2.51 (t, 2H, *J* = 6.9 Hz, Ar-CH_2_-NH-CH_2_-); 1.59–1.51 (m, 2H, -NH-CH_2_-CH_2_-); 1.49–1.42 (m, 2H, -NH-CH_2_-CH_2_-); 1.40–1.27 (m, 4H, -NH-CH_2_-CH_2_-CH_2_-CH_2_-CH_2_-CH_2_-NH-). ^13^C NMR (100 MHz) (CD_3_CN) δ (ppm): 160.2; 148.8; 141.8; 137.8; 132.0; 131.0; 120.6; 112.8; 108.1; 53.5; 49.8; 42.2; 30.7; 30.2; 27.8; 27.6. ESI-MS (*m*/*z*): [M + H]^+^ = 361.76 (90), 363.95 (100). I.R. (cm^–1^): 3269; 1156; 1109; 1067; 1011; 979. m.p. = 42–44 °C. Anal. (C_18_H_24_BrN_3_) C, H, N calcd: C 59.67%, H 6.68%, N 11.60%; found: C 59.69%, H 6.69%, N 11.63%.

##### *N*^1^-(4-Chlorobenzyl)-*N*^6^-(pyridin-2-yl)hexane-1,6-diamine (**27**)

Compound **27** was prepared using *N*^1^-(pyridin-2-yl)hexane-1,6-diamine (**8**) (0.108 g, 0.56 mmol), 4-chlorobenzaldehyde (0.079 g, 0.56 mmol), K_2_CO_3_ (1.548 g, 11.2 mmol), and NaBH_4_ (0.064 g, 1.68 mmol) following procedure 1 of GP-D described above. Column chromatography: silica gel, CH_2_Cl_2_/MeOH 1:1, *R*_f_ = 0.31. Yellow solid, 0.090 g, 51% yield. ^1^H NMR (400 MHz) (CD_3_CN) δ (ppm): 7.96 (d, 1H, *J* = 5.0 Hz, pyridine); 7.36 (t, 1H, *J* = 8.0 Hz, pyridine); 7.31 (s, 4H, aromatic); 6.48 (t, 1H, *J* = 6.0 Hz, pyridine); 6.40 (d, 1H, *J* = 8.4 Hz, pyridine); 5.07 (bs, 1H, pyridine-NH-); 3.70 (s, 2H, Ar-CH_2_-NH-); 3.23 (q, 2H, *J* = 6.6 Hz, pyridine-NH-CH_2_-); 2.52 (t, 2H, *J* = 6.8 Hz, Ar-CH_2_-NH-CH_2_-); 1.59–1.52 (m, 2H, -NH-CH_2_-CH_2_-); 1.49–1.42 (m, 2H, -NH-CH_2_-CH_2_-); 1.40–1.27 (m, 4H, -NH-CH_2_-CH_2_-CH_2_-CH_2_-CH_2_-CH_2_-NH-). ^13^C NMR (100 MHz) (CD_3_CN) δ (ppm): 160.2; 148.8; 141.3; 137.8; 132.6; 130.6; 129.0; 112.8; 108.1; 53.5; 49.8; 42.2; 30.7; 30.1; 27.8; 27.6. ESI-MS (*m*/*z*): [M + H]^+^ = 318.13 (100); 320.13 (35). I.R. (cm^–1^): 3261; 1296; 1156; 1121; 1091; 1084; 1015; 982. m.p. = 41–43 °C. Anal. (C_18_H_24_ClN_3_) C, H, N calcd: C 68.02%, H 7.61%, N 13.22%; found: C 67.97%, H 7.59%, N 13.26%.

##### *N*^1^-(2-Methoxybenzyl)-*N*^6^-(pyridin-2-yl)hexane-1,6-diamine (**28**)

Compound **28** was prepared using *N*^1^-(pyridin-2-yl)hexane-1,6-diamine (**8**) (0.106 g, 0.55 mmol), 2-methoxybenzaldehyde (0.075 g, 0.55 mmol), K_2_CO_3_ (1.520 g, 11.0 mmol), and NaBH_4_ (0.062 g, 1.65 mmol) following procedure 1 of GP-D described above. Column chromatography: silica gel, CH_2_Cl_2_/MeOH 7:3, *R*_f_ = 0.2. Yellow oil, 0.112 g, 65% yield. ^1^H NMR (400 MHz) (CD_3_CN) δ (ppm): 7.96 (dd, 1H, *J_1_* = 5.0 Hz, *J_2_* = 1.1 Hz, pyridine); 7.38–7.34 (m, 1H, pyridine); 7.26–7.20 (m, 2H, aromatic); 6.94–6.88 (m, 2H, aromatic); 6.48 (ddd, 1H, *J_1_* = 7.0 Hz, *J_2_* = 5.0 Hz, *J_3_* = 0.8 Hz, pyridine); 6.40 (d, 1H, *J* = 8.4 Hz, pyridine); 5.07 (bs, 1H, pyridine-NH-); 3.80 (s, 3H, Ar-OCH_3_); 3.68 (s, 2H, Ar-CH_2_-NH-); 3.23 (q, 2H, *J* = 6.9 Hz, pyridine-NH-CH_2_-); 2.52 (t, 2H, *J* = 6.9 Hz, Ar-CH_2_-NH-CH_2_-); 1.59–1.52 (m, 2H, -NH-CH_2_-CH_2_-); 1.49–1.42 (m, 2H, -NH-CH_2_-CH_2_-); 1.40–1.27 (m, 4H, -NH-CH_2_-CH_2_-CH_2_-CH_2_-CH_2_-CH_2_-NH-). ^13^C NMR (100 MHz) (CD_3_CN) δ (ppm): 160.2; 158.5; 148.8; 137.8; 130.2; 129.9; 128.8; 121.1; 112.8; 111.3; 108.1; 55.9; 49.9; 49.3; 42.2; 30.7; 30.2; 27.8; 27.7. ESI-MS (*m*/*z*): [M + H]^+^ = 313.88. I.R. (cm^–1^): 3281; 1289; 1239; 1151; 1100; 1050; 1029; 982. Anal. (C_19_H_27_N_3_O) C, H, N calcd: C 72.81%, H 8.68%, N 13.41%; found: C 72.82%, H 8.71%, N 13.38%.

##### *N*^1^-(Pyridin-2-yl)-*N*^6^-(3,4,5-trimethoxybenzyl)hexane-1,6-diamine (**29**)

Compound **29** was prepared using *N*^1^-(pyridin-2-yl)hexane-1,6-diamine (**8**) (0.102 g, 0.53 mmol), 3,4,5-trimethoxybenzaldehyde (0.104 g, 0.53 mmol), and NaBH_4_ (0.060 g, 1.59 mmol) following procedure 3 of GP-D described above. Column chromatography: silica gel, AcOEt/MeOH/TEA 5:5:0.2, *R*_f_ = 0.53. Orange oil, 0.133 g, 67% yield. ^1^H NMR (400 MHz) (CD_3_CN) δ (ppm): 7.97–7.96 (m, 1H, pyridine); 7.38–7.34 (m, 1H, pyridine); 6.62 (s, 2H, aromatic); 6.48 (ddd, 1H, *J_1_* = 7.0 Hz, *J_2_* = 5.0 Hz, *J_3_* = 0.8 Hz, pyridine); 6.40 (d, 1H, *J* = 8.4 Hz, pyridine); 5.07 (bs, 1H, pyridine-NH-); 3.79 (s, 6H, Ar-(OCH_3_)_2_); 3.68 (s, 3H, -OCH_3_); 3.66 (s, 2H, Ar-CH_2_-NH-); 3.23 (q, 2H, *J* = 6.9 Hz, pyridine-NH-CH_2_-); 2.55 (t, 2H, *J* = 6.9 Hz, Ar-CH_2_-NH-CH_2_-); 1.59–1.52 (m, 2H, -NH-CH_2_-CH_2_-); 1.51–1.45 (m, 2H, -NH-CH_2_-CH_2_-); 1.41–1.27 (m, 4H, -NH-CH_2_-CH_2_-CH_2_-CH_2_-CH_2_-CH_2_-NH-). ^13^C NMR (100 MHz) (MeOD) δ (ppm): 160.5; 154.6; 147.8; 138.7; 138.5; 134.9; 112.9; 109.7; 107.1; 61.1; 56.6; 54.2; 49.5; 42.5; 30.3; 29.6; 28.0; 27.9. ESI-MS (*m*/*z*): [M + H]^+^ = 373.90. I.R. (cm^–1^): 3295; 1234; 1121; 1006. Anal. (C_21_H_31_N_3_O_3_) C, H, N calcd: C 67.53%, H 8.37%, N 11.25%; found: C 67.57%, H 8.34%, N 11.23%.

##### 2-Methoxy-4-(((6-(pyridin-2-ylamino)hexyl)amino)methyl)phenol (**30**)

Compound **30** was prepared using *N*^1^-(pyridin-2-yl)hexane-1,6-diamine (**8**) (0.102 g, 0.53 mmol), 4-hydroxy-3-methoxybenzaldehyde (0.081 g, 0.53 mmol), and NaBH_4_ (0.060 g, 1.59 mmol) following procedure 3 of GP-D with some modifications: after the reaction with NaBH_4_, the addition of HCl, and the removal of the solvent, the residue was washed with warm AcOEt and then the solid was diluted with 20 mL of a saturated aqueous solution of NaHCO_3_ and extracted with AcOEt (3 × 20 mL). The organic layer was dried over Na_2_SO_4_, and the solvent was removed under vacuum. Yellow oil, 0.140 g, 80% yield. ^1^H NMR (400 MHz) (CD_3_CN) δ (ppm): 7.96 (dd, 1H, *J_1_* = 5.0 Hz, *J_2_* = 1.1 Hz, pyridine); 7.39–7.34 (m, 1H, pyridine); 6.92 (s, 1H, aromatic); 6.74 (s, 2H, aromatic); 6.48 (ddd, 1H, *J_1_* = 7.0 Hz, *J_2_* = 5.0 Hz, *J_3_* = 0.8 Hz, pyridine); 6.40 (d, 1H, *J* = 8.4 Hz, pyridine); 5.08 (bs, 1H, pyridine-NH-); 3.82 (s, 3H, -OCH_3_); 3.63 (s, 2H, Ar-CH_2_-NH-); 3.23 (q, 2H, *J* = 6.9 Hz, pyridine-NH-CH_2_-); 2.53 (t, 2H, *J* = 7.0 Hz, Ar-CH_2_-NH-CH_2_-); 1.59–1.52 (m, 2H, -NH-CH_2_-CH_2_-); 1.50–1.43 (m, 2H, -NH-CH_2_-CH_2_-); 1.40–1.27 (m, 4H, -NH-CH_2_-CH_2_-CH_2_-CH_2_-CH_2_-CH_2_-NH-). ^13^C NMR (100 MHz) (MeOD) δ (ppm): 160.5; 149.1; 147.8; 147.3; 138.7; 130.4; 122.7; 116.2; 113.4; 112.9; 109.7; 56.4; 54.0; 49.4; 42.5; 30.3; 29.7; 28.1; 27.9. ESI-MS (*m*/*z*): [M + H]^+^ = 330.20. I.R. (cm^–1^): 1274; 1152; 1125; 1033; 982. Anal. (C_19_H_27_N_3_O_2_) C, H, N calcd: C 69.27%, H 8.26%, N 12.76%; found: C 69.19%, H 8.28%, N 12.80%.

##### 3-(((6-(Pyridin-2-ylamino)hexyl)amino)methyl)phenol (**31**)

Compound **31** was prepared using *N*^1^-(pyridin-2-yl)hexane-1,6-diamine (**8**) (0.075 g, 0.39 mmol), 3-hydroxybenzaldehyde (0.048 g, 0.39 mmol), and NaBH_4_ (0.044 g, 1.17 mmol) following procedure 3 of GP-D described above. In this case, the extraction was carried out with AcOEt instead of CH_2_Cl_2_. Column chromatography: silica gel, AcOEt/MeOH/TEA 5:5:0.1, *R*_f_ = 0.22. White solid, 0.062 g, 53% yield. ^1^H NMR (400 MHz) (acetone-*d*_6_) δ (ppm): 7.98–7.96 (m, 1H, pyridine); 7.36–7.32 (m, 1H, pyridine); 7.09 (t, 1H, *J* = 7.8 Hz, aromatic); 6.86 (s, 1H, aromatic); 6.79 (d, 1H, *J* = 7.5, aromatic); 6.67 (dd, 1H, *J_1_* = 7.8 Hz, *J_2_* = 1.8 Hz, aromatic); 6.46–6.43 (m, 2H, pyridine); 5.64 (bs, 1H, pyridine-NH-); 3.67 (s, 2H, Ar-CH_2_-NH-); 3.33–3.28 (m, 2H, pyridine-NH-CH_2_-); 2.56 (t, 2H, *J* = 6.9 Hz, Ar-CH_2_-NH-CH_2_-); 1.63–1.56 (m, 2H, -NH-CH_2_-CH_2_-); 1.53–1.46 (m, 2H, -NH-CH_2_-CH_2_-); 1.43–1.34 (m, 4H, -NH-CH_2_-CH_2_-CH_2_-CH_2_-CH_2_-CH_2_-NH-). ^13^C NMR (100 MHz) (MeOD) δ (ppm): 160.4; 158.9; 147.8; 140.4; 138.7; 130.6; 120.7; 116.6; 115.6; 112.9; 109.7; 53.9; 49.5; 42.5; 30.3; 29.6; 28.0; 27.9. ESI-MS (*m*/*z*): [M + H]^+^ = 300.40. I.R. (cm^–1^): 3270; 1281; 1153; 1112; 926. m.p. = 86–88 °C. Anal. (C_18_H_25_N_3_O) C, H, N calcd: C 72.21%, H 8.42%, N 14.03%; found: C 72.17%, H 8.44%, N 14.06%.

##### 3-(((6-(Pyridin-2-ylamino)hexyl)amino)methyl)benzene-1,2-diol (**32**)

Compound **32** was prepared using *N*^1^-(pyridin-2-yl)hexane-1,6-diamine (**8**) (0.102 g, 0.53 mmol), 2,3-dihydroxybenzaldehyde (0.073 g, 0.53 mmol), and NaBH_4_ (0.060 g, 1.59 mmol) following procedure 1 of GP-D described above. The compound was purified by washing with CH_3_CN. White solid, 0.084 g, 50% yield. ^1^H NMR (400 MHz) (MeOD) δ (ppm): 7.89–7.88 (m, 1H, pyridine); 7.42–7.38 (m, 1H, pyridine); 6.69 (t, 1H, *J* = 4.6 Hz, aromatic); 6.56 (d, 2H, *J* = 4.7 Hz, aromatic); 6.52–6.48 (m, 2H, pyridine); 3.90 (s, 2H, Ar-CH_2_-NH-); 3.24 (t, 2H, *J* = 7.0 Hz, pyridine-NH-CH_2_-); 2.67 (t, 2H, *J* = 7.2 Hz, Ar-CH_2_-NH-CH_2_-); 1.64–1.55 (m, 4H, -NH-CH_2_-CH_2_-CH_2_-CH_2_-CH_2_-CH_2_-); 1.42–1.40 (m, 4H, -NH-CH_2_-CH_2_-CH_2_-CH_2_-CH_2_-CH_2_-NH-). ^13^C NMR (100 MHz) (*d*_6_-DMSO) δ (ppm): 159.0; 147.6; 146.0; 145.2; 136.5; 123.7; 118.8; 118.0; 114.3; 111.2; 107.9; 51.1; 48.0; 40.7; 29.0; 28.9; 26.59; 26.55. ESI-MS (*m*/*z*): [M + H]^+^ = 315.92. I.R. (cm^–1^): 1292; 1280; 1199; 1067. m.p. = 112–114 °C. Anal. (C_18_H_25_N_3_O_2_) C, H, N calcd: C 68.54%, H 7.99%, N 13.32%; found: C 68.61%, H 7.98%, N 13.28%.

##### *N*^1^-((2,6-Dichloropyridin-4-yl)methyl)-*N*^6^-(pyridin-2-yl)hexane-1,6-diamine (**33**)

Compound **33** was prepared using *N*^1^-(pyridin-2-yl)hexane-1,6-diamine (**8**) (0.108 g, 0.56 mmol), 2,6-dichloropyridine-4-carbaldehyde (0.099 g, 0.56 mmol), K_2_CO_3_ (1.548 g, 11.2 mmol), and NaBH_4_ (0.064 g, 1.68 mmol) following procedure 1 of GP-D described above. Column chromatography: silica gel, AcOEt/MeOH 9.5:0.5, *R*_f_ = 0.32. White solid, 0.131 g, 66% yield. ^1^H NMR (400 MHz) (CD_3_CN) δ (ppm): 7.96 (dd, 1H, *J_1_* = 5.0 Hz, *J_2_* = 1.0 Hz, pyridine); 7.39–7.24 (m, 3H, 1H pyridine and 2H 2,6-dichloropyridine); 6.48 (ddd, 1H, *J_1_* = 7.0 Hz, *J_2_* = 5.0 Hz, *J_3_* = 0.8 Hz, pyridine); 6.40 (d, 1H, *J* = 8.4 Hz, pyridine); 5.07 (bs, 1H, pyridine-NH-); 3.75 (s, 2H, 2,6-dichloropyridine-CH_2_-NH-); 3.23 (q, 2H, *J* = 6.9 Hz, pyridine-NH-CH_2_-); 2.51 (t, 2H, *J* = 6.8 Hz, 2,6-dichloropyridine-CH_2_-NH-CH_2_-); 1.59–1.52 (m, 2H, -NH-CH_2_-CH_2_-); 1.49–1.43 (m, 2H, -NH-CH_2_-CH_2_-); 1.41–1.27 (m, 4H, -NH-CH_2_-CH_2_-CH_2_-CH_2_-CH_2_-CH_2_-NH-). ^13^C NMR (100 MHz) (MeOD) δ (ppm): 160.5; 157.8; 151.5; 147.8; 138.7; 123.6; 112.9; 109.7; 52.4; 50.1; 42.6; 30.5; 30.4; 28.1; 28.0. ESI-MS (*m*/*z*): [M + H]^+^ = 353.05 (90), 354.98 (100), 356.91 (15). I.R. (cm^–1^): 3237; 1246; 1212; 1154; 1128; 1085; 986. m.p. = 61–64 °C. Anal. (C_17_H_22_Cl_2_N_4_) C, H, N calcd: C 57.79%, H 6.28%, N 15.86%; found: C 57.83%, H 6.30%, N 15.81%.

### Enzymatic Assays

#### Materials and Methods

*Electric eel* AChE (*Ee*AChE, EC 3.1.1.7), *equine* BChE (EC 3.1.1.8), acetylthiocholine iodide, 5,5′-dithio-bis-(2-nitrobenzoic acid) (DTNB), tacrine, and donepezil, used as the reference standard, were purchased from Sigma-Aldrich (Milan, Italy). All other chemical and biological reagents and solvents used were of the highest analytical, commercially available grade. The water, utilized for the preparation of the phosphate buffer and of the compound solutions, was distilled and filtered on nylon membrane filters with a 0.2 μm pore size with the Millipore Filtration apparatus before each use. Micropipettes Labmate (BRAND Dig. 10–100 μL; Dig. 100–1000 μL; Dig. 0.1–2 μL) and Transferpette (HIGH TECH LAB LM200: 20–200 μL; LM5000: 1000–5000 μL) were used to collect the samples. The assays were carried out by a double beam UV–vis Lambda 40 Perkin Elmer spectrophotometer using optical polystyrene cuvettes (10 × 10 × 45 mm, 340–800 nm optical transparency); each measure was repeated at least in triplicate. For data processing, UV-WIN Lab version 2.0 (Perkin Elmer Corporation) and SigmaPlot version 8.02 were used. The spectrophotometric method of Ellman^25^ with minor modifications was used to evaluate the inhibition of ChEs. This method is based on the reaction of released thiocholine to give a colored product, at a wavelength of 412 nm, with the chromogenic reagent DTNB. The absorbance was recorded at 412 nm between 0 and 1.6 min, and the absorbance variation, utilized for the kinetic assay, was measured between 0.5 and 1.5 min to allow the stabilization of the UV–vis lamp and of the solution. The method is extremely sensitive to variations in the order of microliters: the standard deviations (less than 5%) of the values obtained are compatible with the experimental errors associated with the use of micropipettes. Each tested compound was dissolved in the opportune quantity of DMSO to obtain a final cuvette DMSO content <0.033%, which does not affect the enzyme activity. *Ee*AChE and *eq*BChE were periodically tested to evaluate the effective enzymatic activity.

#### Determination of Kinetic Parameters of *EeAChE* and *eqBChE*

The Michaelis constant (*K*_m_) and the maximum reaction velocity (*V*_max_) of *Ee*AChE and *eq*BChE were determined using acetylthiocholine as substrate. For the assay, five different concentrations of acetylthiocholine were used (range 50–600 μM for *Ee*AChE and 100–800 μM for *eq*BChE).

Three milliliters of 0.1 M phosphate buffer (pH = 7.4) containing DTNB (0.25 mM) and *Ee*AChE (0.083 UmL^–1^) or *eq*BChE (0.083 UmL^–1^) was placed in a polystyrene cuvette of 1.0 cm path length. With this solution, the blank was made. To start the reaction, the opportune volume of a solution in 0.1 M phosphate buffer (pH = 7.4) of acetylthiocholine (10 mM or 20 mM, to introduce in the cuvette volumes ranging from 15 to 120 μL) was added to obtain the desired final concentration of substrate. Each determination was repeated for five times. The absorbance variations were measured at 412 nm at 25 °C between 0.5 and 1.5 min. From these data, the values of the enzymatic hydrolysis rate, expressed as μmol of substrate hydrolyzed in 1 min by an enzymatic unit, were obtained. The recorded data were analyzed by the enzyme kinetic module of SigmaPlot (version 8.02) based on the Michaelis–Menten equation.

*K*_m_ = 145.8 ± 11.5 μM and *V*_max_ = 348.6 ± 9.9 μmol/min, with *R*^2^ = 0.977, were determined for *Ee*AChE. *K*_m_ = 443.9 ± 16.5 μM and *V*_max_ = 178.5 ± 3.1 μmol/min, with *R*^2^ = 0.997, were determined for *eq*BChE.

#### Percent Inhibition of *EeAChE* and *eqBChE*

For all the synthesized compounds, the percentages of inhibition toward AChE of *Electrophorus electricus* (*Ee*AChE) and equine BChE (*eq*BChE) were evaluated.

Three milliliters of a solution in 0.1 M phosphate buffer (pH = 7.4) containing DTNB (0.25 mM) and *Ee*AChE (0.083 UmL^–1^) or *eq*BChE (0.083 UmL^–1^) was placed in a polystyrene cuvette of 1.0 cm path length; 1 μL of a solution in DMSO of the tested compound was added to obtain a cuvette concentration range of 9–0.09 μM. With this solution, the blank was made. To start the reaction, 30 μL of a solution in 0.1 M phosphate buffer (pH = 7.4) of acetylthiocholine (10 mM) was added to obtain a final concentration of acetylthiocholine equal to 100 μM. The increase in the absorbance, due to the production of the yellow 2-nitro-5-thiobenzoic anion, was recorded at 412 nm at 25 °C, and the absorbance variation was measured between 0.5 and 1.5 min. As a control, an identical solution of the enzyme without the inhibitor was processed following the same protocol to determine 100% of the enzyme activity. Each experiment was repeated at least in triplicate. The potency of each compound to inhibit *Ee*AChE or *eq*BChE activity was expressed as percent inhibition calculated using the following equation:
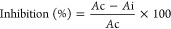
where *A*_i_ and *A*_c_ represent the average of the absorbance variation in the presence of inhibitor and without inhibitor, respectively.

#### Determination of Constant and Mechanism of Inhibition *vs EeAChE* and *eqBChE*

The constants and the mechanisms of inhibition were determined for a selection of compounds that are among the most potent as inhibitors of *Ee*AChE and *eq*BChE (**9**, **13**, **19**, **23**, **25**, and **28** toward *Ee*AChE; **18**, **22**, **25**, **26**, **28**, and **30** toward *eq*BChE). For each compound, a 500 μM stock solution was prepared in H_2_O/DMSO or in 10^–2^ M HCl and diluted in water to prepare solutions of opportune concentrations to introduce in the cuvette volumes ranging from 30 to 100 μL, to obtain the desired final concentrations. The maximum amount of DMSO used for the preparation of the stock solution was calculated to have a DMSO content in cuvette <0.033%.

Three milliliters of 0.1 M phosphate buffer (pH = 7.4) containing DTNB (0.25 mM) and *Ee*AChE (0.083 UmL^–1^) or *eq*BChE (0.083 UmL^–1^) was mixed with the opportune volume of the inhibitor solution to obtain a final inhibitor concentration in cuvette between 90 and 1800 nM. With this solution, the blank was made. The reaction was started by adding to the enzyme–inhibitor mixture the proper volume of a solution in 0.1 M phosphate buffer (pH = 7.4) of acetylthiocholine (10 mM) to obtain a final concentration of substrate equal to 100–400 μM. Each determination was repeated five times. The absorbance variations were measured at 412 nm at 25 °C between 0.5 and 1.5 min. From these data, the values of the enzymatic hydrolysis rate, expressed as μmol of substrate hydrolyzed in 1 min by an enzymatic unit, were obtained.

To determine the inhibition constants (*K*_i_) and the inhibition mechanisms, the rates of hydrolysis at three different concentrations of substrate in the presence of five different concentrations of inhibitor were measured. The recorded data were analyzed by the enzyme kinetic module of SigmaPlot (version 8.02) plotting the reciprocal of the rate of hydrolysis (1/v, min/μM) *vs* the concentration of the inhibitor (nM) according to Dixon’s method^26^ to find the best fitting model of inhibition, as indicated by the calculated linear regression coefficient *R*^2^.

#### Determination of IC_50_*vs EeAChE* for Compound **20**, Tacrine, and Donepezil

The IC_50_ value of compound **20***vs Ee*AChE and the IC_50_ values of tacrine and donepezil *vs* both ChEs were calculated. For this purpose, eight different inhibitor solutions were prepared. For compound **20**, solutions in 10^–2^ M HCl were prepared, having suitable concentrations, so as to add 20 μL of these solutions to the assay mixture to obtain a final concentration between 0.05 and 10 μM; for tacrine and donepezil, solutions in DMSO were prepared, having suitable concentrations, so as to add 1 μL of these solutions to the assay mixture to obtain a final concentration between 0.5 and 27,000 nM. For each inhibitor concentration, the percentage of inhibition in the presence of *Ee*AChE or *eq*BChE (0.0833 U mL^–1^) and acetylthiocholine (100 μM) was measured as described above. The recorded data were analyzed by the enzyme kinetic module of SigmaPlot plotting the percentages of inhibition as a function of the different concentrations of the inhibitor, expressed in logarithmic scale, to obtain the sigmoid graph, from which it was possible to determine the IC_50_ value of the tested compound. The IC_50_ was confirmed by repeating the experiment twice.

### Computational Studies and ADME Prediction

All computational studies were carried out by means of Schrödinger Suite 2018-1.^42^ The X-ray crystallographic structures of the *h*AChE in complex to donepezil (PDB code:4EY7)^13^ and the *h*BChE in complex to the naphthamide derivative (PDB code:5NN0)^27^ were used. *h*ChEs structures were prepared by using the Maestro Protein Preparation Wizard^43^ tool and were refined to optimize hydrogen bonds and energy minimized by using OPLS_2005 as force field at pH 7.4.^44−46^ The homology between the human and not human ChE isoforms used for the enzymatic assays was assessed by the sequence alignment performed by Prime.^47^

All derivatives were submitted to 5000 steps of Monte Carlo conformational search applied to all rotatable bonds. The water solvent effect was considered using the implicit model GB/SA.^48^ The global minimum energy structures were used to carry out molecular docking simulations.

Then, target binding sites were defined by means of a regular grid box of about 27,000 Å^3^ centered on the catalytic serine residues. All docking simulations were computed using the Glide^49^ ligand flexible algorithm at the standard-precision (SP) level. The best docked poses of the most potent inhibitors resulted by *in vitro* assays of pyrimidine and pyridine derivatives were submitted to 250 ns of molecular dynamic (MD) simulations by using Desmond ver. 4.2.^50^ The system was solvated in the SPC explicit solvent model, and counter ions were added to neutralize the system net charge. After optimization of the solvated model, we relaxed the system with the Martyna–Tobias–Klein isobaric–isothermal ensemble (MTK_NPT). This preliminary stage included two energy minimizations of 2000 steps: in the first run, the system was restrained with a force constant of 50 kcal mol^–1^ A^–1^, while in the second one, all the system was released without any restrains. The following conditions for MDs were used: the NPT ensemble, a temperature of 300 K, a pressure of 1 bar, a Berendsen thermostat–barostat, and a recording interval equal to 250 ps both for energy and for trajectory collecting 1000 frames for each simulation.

To identify possible PAINS, all synthesized final molecules were screened using *in silico* public tools available at https://zinc15.docking.org/patterns/home/ and http://www.swissadme.ch/.

ADME descriptors of the most potent compounds were predicted using the SwissADME public server.^38^ The following ADME parameters have been selected: molecular weight (MW); number of H-bond acceptors (H-b acc); number of H-bond donors (H-b don); number of heavy atoms (heavy atoms); number of rotatable bonds (rot bonds); topological polar surface area in Å^2^ (TPSA); octanol/water partition coefficient (MLogP); water solubility (LogS ESOL); water solubility class (Sol class); gastrointestinal absorption (GI); blood–brain barrier permeation (BBB); and number of Lipinski’s rule of five violations (Lipinski viol).^39^

### Chelation Studies

#### Materials and Methods

FeCl_3_·6H_2_O, CuSO_4_·5H_2_O, Zn(NO_3_)_2_·6H_2_O, and MeOH used for chelation studies were purchased from Sigma-Aldrich. Micropipettes Labmate (BRAND Dig. 10–100 μL; Dig. 100–1000 μL) and Transferpette (HIGH TECH LAB LM200: 20–200 μL; LM5000: 1000–5000 μL) were used to collect the samples. The assays were carried out by a double beam UV–vis Lambda 40 Perkin Elmer spectrophotometer using quartz cuvettes (Optech, type S/Q/10). Data were elaborated using UV-WIN Lab Version 2.0 (Perkin Elmer Corporation), Microsoft Excel 2010, and Spekwin32. For each measurement, the absorbance values were recorded immediately after mixing the solutions.

#### UV–Vis Titration

For the UV–vis titration, ligand solutions in MeOH were prepared: 10^–3^ M for compounds **9**, **13**, **20**, **25**, **28**, and **30**; 5 × 10^–3^ M for compounds **18** and **19**. Stock solutions (1 M) of CuSO_4_·5H_2_O and Zn(NO_3_)_2_·6H_2_O in water and 0.1 M of FeCl_3_·6H_2_O in MeOH were prepared and diluted with MeOH to obtain different solutions, having suitable concentrations, so as to add 2.85 mL of these solutions to 150 μL of the ligand solution to obtain in 3 mL of the assay mixture a final molar ratio of metal ion to ligand between 0.1 and 20. UV–vis spectra were recorded from 210 to 450 nm.

For the first measure, 150 μL of the ligand solution was diluted in 2.85 mL of MeOH into a sample cuvette, while only MeOH was placed in the reference cuvette and the UV–vis spectrum was recorded. For the subsequent measurements, 2.85 mL of the opportune metal solution was placed in both the sample cuvette and reference cuvette; then 150 μL of the ligand solution was added to the sample cuvette, while 150 μL of MeOH was added to the reference cuvette, and the UV–vis spectra were recorded. The spectra thus obtained were superimposed to observe the variations present between the ligand alone and in the presence of an increasing amount of metal.

#### Job’s Plot

To determine the stoichiometric coefficients of the complexes, Job’s method^33^ was used, which requires to mix, in appropriate proportions, an equimolar solution of metal ion and ligand so that the final volume and the total moles present in the cuvette are equal for each measurement. The absorbance values were recorded at the wavelengths, extrapolated from the UV–vis titration spectra, where the maximum variation of absorbance was observed. At the same time, two solutions in MeOH of equal concentration were prepared, one of ligand and one of metal ion, diluting the solutions 1 M of CuSO_4_·5H_2_O and Zn(NO_3_)_2_·6H_2_O and 0.1 M of FeCl_3_·6H_2_O previously prepared. The concentrations of the solutions used and the wavelengths in which the absorbance was recorded were as follows: 4.96 × 10^–4^ M for **18** and Fe^3+^ −258, 375 nm; 5.02 × 10^–4^ M for **18** and Cu^2+^ −330 nm; 5.66 × 10^–4^ M for **19** and Fe^3+^ −292, 375 nm; 5.66 × 10^–4^ M for **19** and Cu^2+^ −312, 330 nm; 3.10 × 10^–4^ M for **20** and Fe^3+^ −370 nm; 3.35 × 10^–4^ M for **20** and Cu^2+^ −330 nm; 3.00 × 10^–4^ M for **25** and Fe^3+^ −251, 318 nm; 3.99 × 10^–4^ M for **25** and Cu^2+^ −330 nm; 3.00 × 10^–4^ M for **28** and Fe^3+^ −316, 374 nm; 3.99 × 10^–4^ M for **28** and Cu^2+^ −330 nm; 3.28 × 10^–4^ M for **30** and Fe^3+^ −310, 372 nm; 3.58 × 10^–4^ M for **30** and Cu^2+^ −312, 330 nm.

For each determination, 9–24 measurements were made, introducing appropriate volumes of metal and ligand solutions in the sample cuvette to obtain mole fractions of the ligand in the range of 0.1–0.95, while appropriate volumes of ligand solution and MeOH were placed in the reference cuvette, to obtain the same concentration of the ligand in the sample cuvette. The absorbance value of the metal ion was calculated by the Lambert–Beer relation, being known its extinction coefficients ε at used wavelengths; for each measure, the nominal absorbance due to the metal ion was algebraically subtracted from the recorded absorbance values to obtain the exclusive absorbance variations due to the complex formation. The resulting Δ*A* values were reported in a graph as a function of the mole fraction of the ligand, and through the intersections of the linear regression lines, the mole fraction *X*, which caused the maximum variation in absorbance, was determined and used to calculated the value of the coefficient *n*, which corresponds to the number of ligand molecules per cation, applying the following equation:
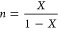


### Antioxidant Activity

#### Materials and Methods

2,2-Diphenyl-1-picrylhydrazyl (DPPH), MeOH, and ascorbic acid, used as the reference standard, were purchased from Sigma-Aldrich. Micropipettes Labmate (BRAND Dig. 10–100 μL; Dig. 100–1000 μL) and Transferpette (HIGH TECH LAB LM200: 20–200 μL; LM5000: 1000–5000 μL) were used to collect the samples. The assays were carried out by a double beam UV–vis Lambda 40 Perkin Elmer spectrophotometer using optical polystyrene cuvettes (10 × 10 × 45 mm, 340–800 nm optical transparency); each measure was repeated at least in triplicate. For data processing, UV-WIN Lab version 2.0 (Perkin Elmer Corporation) was used. The antioxidant activity of the tested compounds was evaluated by the DPPH method^34^ based on the ability of antioxidant compounds to react with the stable radical DPPH, which has a maximum absorption at about 515 nm. Thus, by measuring the decrease in absorbance over time of DPPH solutions to which increasing amounts of the tested compound are added, the antioxidant activity of the compound can be determined.

#### Antioxidant Activity Tests

To assess the antioxidant activity of **18**, **19**, **20**, and **30**, at first, the decrease in absorbance over time at 515 nm of a solution of DPPH and the tested compound was recorded until the completion of the reaction. To 2.90 mL of MeOH, 60 μL of a DPPH solution in MeOH (∼1.2 mM) and 40 μL of an antioxidant solution in MeOH (4.5 mM) were added to have in the cuvette concentrations of DPPH equal to about 25 μM and of antioxidant equal to 60 μM. The exact DPPH concentration was calculated through the Lambert–Beer relation, known the extinction coefficient of DPPH at 515 nm, equal to 1.25 × 10^4^.^34^ The absorbance was recorded until the plateau was reached.

Subsequently, for compounds that showed antioxidant activity (**18**, **20**, and **30**), the EC_50_ values were determined. To obtain the initial DPPH concentration, 2.94 mL of MeOH and 60 μL of the DPPH solution in MeOH (∼1.2 mM) were placed in a polystyrene cuvette and the absorbance was recorded at 515 nm. The exact initial DPPH concentration was calculated through the Lambert–Beer relation. For subsequent measurements, the cuvettes were set up by introducing the opportune volume of MeOH to reach a total volume of 3 mL, 60 μL of the DPPH solution in MeOH (∼1.2 mM, to have a final concentration of ∼25 μM), and the appropriate volumes of the antioxidant stock solution in MeOH to have final concentrations between 1.5 and 12 μM for **20** and between 5 and 60 μM for **18** and **30**; the absorbance was recorded at 515 nm at the plateau and then after 1 min for compound **20** and after 90 min for compounds **18** and **30**. Each measure was repeated four times. With absorbance data, the percentages of residual DPPH at steady state were obtained using the following equation:
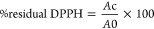
where *A*_c_ is the average of absorbance in the presence of antioxidant at plateau and *A*_0_ is the average of the initial absorbance of DPPH.

By plotting the percentage of residual DPPH at the steady state as a function of the molar ratio of the antioxidant to initial DPPH, it is possible to obtain the value of EC_50_, defined by the ratio
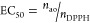
were *n*_ao_ is the moles of antioxidant necessary to reduce the initial concentration of DPPH by 50% and *n*_DPPH_ is the initial moles of DPPH.^35^ The same procedure was performed using ascorbic acid as the antioxidant at cuvette concentrations between 1.5 and 18 μM, recording the absorbance at 515 nm after 2 min from the preparation of the cuvettes. In this way, an EC_50_ equal to 0.265 ± 0.007 was measured, in accordance with literature.^35^

### Inhibition of Amyloid and Tau Aggregation

#### Cloning and Overexpression of the Aβ_42_ Peptide

*E. coli* competent cells BL21 (DE3) were transformed with the pET28a vector (Novagen, Inc., Madison, WI, USA) carrying the DNA sequence of Aβ_42_. Because of the addition of the initiation codon ATG in front of both genes, the overexpressed peptide contains an additional methionine residue at its N terminus. For overnight culture preparation, an amount of 10 mL of the M9 minimal medium containing 50 μg·mL^–1^ of kanamycin was inoculated with a colony of BL21 (DE3) bearing the plasmid to be expressed at 37 °C. For expression of the Aβ_42_ peptide, the required volume of overnight culture to obtain 1:500 dilution was added into the fresh M9 minimal medium containing 50 μg·mL^–1^ of kanamycin and 250 μM Th-S. The bacterial culture was grown at 37 °C and 250 rpm. When the cell density reached OD600 = 0.6, an amount of 980 μL of culture was transferred into Eppendorf tubes of 1.5 mL with 10 μL of each compound to be tested in DMSO and 10 μL of isopropyl 1-thio-β-d-galactopyranoside (IPTG) at 100 mM. The final concentration of the drug was fixed at 100 μM. The samples were grown overnight at 37 °C and 1400 rpm using a ThermoMixer (Eppendorf, Hamburg, Germany). As negative control (maximal amyloid presence), the same amount of DMSO without the drug was added in the sample. In parallel, non-induced samples (in the absence of IPTG) were also prepared and used as positive controls (non-amyloid presence). In addition, these samples were used to assess the potential intrinsic toxicity of the compounds and to confirm the correct bacterial growth.

#### Cloning and Overexpression of the Tau Protein

*E. coli* BL21 (DE3) competent cells were transformed with pTARA containing the RNA-polymerase gene of T7 phage (T7RP) under the control of the promoter PBAD. *E. coli* BL21 (DE3) with pTARA competent cells were transformed with the pRKT42 vector encoding four repeats of the tau protein in two inserts. For overnight culture preparation, 10 mL of the M9 medium containing 0.5% of glucose, 50 μg·mL^–1^ of ampicillin, and 12.5 μg·mL^–1^ of chloramphenicol was inoculated with a colony of BL21 (DE3) bearing the plasmids to be expressed at 37 °C. For expression of the tau protein, the required volume of overnight culture to obtain 1:500 dilution was added to the fresh M9 minimal medium containing 0.5% of glucose, 50 μg·mL^–1^ of ampicillin, 12.5 μg·mL^–1^ of chloramphenicol, and 250 μM Th-S. The bacterial culture was grown at 37 °C and 250 rpm. When the cell density reached OD600 = 0.6, an amount of 980 μL of culture was transferred into Eppendorf tubes of 1.5 mL with 10 μL of each compound to be tested in DMSO and 10 μL of arabinose at 25%. The final concentration of the drug was fixed at 100 μM. The samples were grown overnight at 37 °C and 1400 rpm using a ThermoMixer (Eppendorf, Hamburg, Germany). As negative control (maximal presence of tau), the same amount of DMSO without the drug was added in the sample. In parallel, non-induced samples (in the absence of arabinose) were also prepared and used as positive controls (absence of tau). In addition, these samples were used to assess the potential intrinsic toxicity of the compounds and to confirm the correct bacterial growth.

#### Thioflavin S (Th-S) Steady-State Fluorescence

Th-S (T1892) and other chemical reagents were purchased from Sigma (St. Louis, MO). The Th-S stock solution (2500 mM) was prepared in double-distilled water purified through a Milli-Q system (Millipore, USA). Th-S fluorescence and absorbance were tracked using a DTX 800 plate reader Multimode Detector equipped with a Multimode Analysis Software (Beckman-Coulter, USA). Filters of 430/35 and 485/20 nm were used for the excitation and emission wavelengths, respectively. Filters of 535/25 nm were also used for the absorbance determination. To normalize the Th-S fluorescence as a function of the bacterial concentration, OD600 was obtained using a Shimadzu UV-2401 PC UV–vis spectrophotometer (Shimadzu, Japan). Note that the fluorescence normalization was carried out considering as 100% the Th-S fluorescence of the bacterial cells expressing the peptide or protein in the absence of drug and 0% the Th-S fluorescence of the bacterial cells not expressing the peptide or protein. A minimum of five independent assays (with three replicates for assay) was performed for each tested compound. More assays were performed to obtain a SEM < 5% with a maximum of 10 independent assays.

### Cytotoxicity Assays

The compounds **9**, **13**, **19**, **20**, **22**, **23**, **25**, **26**, and **28** were evaluated for cell viability effects using the MTT assay. Briefly, the U-87 MG cell line from the human brain (glioblastoma astrocytoma) (ATCC, Manassas, VA, USA) was seeded into 96-well microtiter plates (Nunclon, Nunc, Germany) at a density of 1.5 × 10^4^ cells/well.^51,52^ After 24 h, cells were exposed to increased concentrations of tested compounds (1, 5, 10, and 50 μM) in the cell culture medium. After an incubation time of 24 h, the medium was replaced by a new fresh medium containing 0.5 mg/mL MTT (Sigma, Deisenhofen, Germany). After 2 h at 37 °C in 5% CO_2_, unreacted dye was removed and cells were dissolved by DMSO (Merck, Darmstadt, Germany). Absorbance was read at 570 nm by a microtiter spectrophotometer plate reader. The data were expressed as absorbance relative to untreated cells in the same experiment and standardized to 100%. All data points were performed in triplicate and at least three independent experiments.

## Supporting Information Available

4EY7_9 (PDB)
4EY7_13 (PDB)
4EY7_19 (PDB)
4EY7_20 (PDB)
4EY7_22 (PDB)
4EY7_30 (PDB)
Molecular formula strings and some data (CSV)
The plots of enzyme inhibition studies on selected compounds, additional figures of molecular docking studies, UV–vis titration spectra, and Job’s plots for selected compounds (PDF)

## Supplementary Material

cn1c00485_si_001.pdb

cn1c00485_si_002.pdb

cn1c00485_si_003.pdb

cn1c00485_si_004.pdb

cn1c00485_si_005.pdb

cn1c00485_si_006.pdb

## Abbreviations Used

AD: Alzheimer’s disease
ACh: acetylcholine
Aβ: β-amyloid peptide
ChAT: choline acetyl transferase
ChEIs: cholinesterase inhibitors
ChEs: cholinesterases
FDA: Food and Drug Administration
AChE: acetylcholinesterase
BChE: butyrylcholinesterase
CAS: catalytic active site
PAS: peripheral anionic site
*h*AChE: human AChE
*h*BChE: human BChE
TEA: triethylamine
TFA: trifluoroacetic acid
DMF: dimethylformamide
*Ee*AChE: *Electrophorus electricus* AChE
*eq*BChE: equine BChE
*K*_i_: inhibition constant
RMSD: root mean square deviation
MD: molecular dynamics
DPPH: 2,2-diphenyl-1-picrylhydrazyl
TPSA: topological polar surface area in Å^2^
MLogP: octanol/water partition coefficient
LogS ESOL: water solubility
GI: gastrointestinal absorption
BBB: blood–brain barrier permeation
PAINS: pan assay interference compounds
