# Supplementary material for: New Pyrimidine and Pyridine Derivatives as Multitarget Cholinesterase Inhibitors: Design, Synthesis, and In Vitro and In Cellulo Evaluation
Source: ACS Chem Neurosci. 2021 Oct 15;12(21):4090–112. doi: 10.1021/acschemneuro.1c00485 (PMC8569681; doi:10.1021/acschemneuro.1c00485)
Supplement: Supplementary file 8 — cn1c00485_si_008.pdf [file cn1c00485_si_008.pdf]

SUPPORTING INFORMATION

New pyrimidine and pyridine derivatives as multitarget cholinesterases inhibitors: design, synthesis, and *in vitro* and *in cellulo* evaluation

Martina Bortolami [a], Fabiana Pandolfi [a], Valeria Tudino [b], Antonella Messore [b], Valentina Noemi Madia [b], Daniela De Vita [c], Roberto Di Santo [b,d], Roberta Costi [b,d], Isabella Romeo [e,f], Stefano Alcaro [e,f,], Marisa Colone [g], Annarita Stringaro [g], Alba Espargaró [h], Raimon Sabatè [h], Luigi Scipione [b,*]*

[a] Department of Scienze di Base e Applicate per l'Ingegneria, Sapienza University of Rome, via Castro Laurenziano 7, I-00161, Rome, Italy

[b] Department of Chimica e Tecnologia del Farmaco, Sapienza University of Rome, Piazzale Aldo Moro 5, 00185, Rome, Italy

[c] Department of Environmental Biology, Sapienza University of Rome, Piazzale Aldo Moro 5, 00185, Rome, Italy

[d] Istituto Pasteur, Fondazione Cenci Bolognetti, Department of Chemistry and Technology of Drug, Sapienza University of Rome, Piazzale Aldo Moro 5, 00185, Rome, Italy

[e] Net4Science s.r.l., Campus universitario "S. Venuta", Viale Europa, 88100, Catanzaro, Italy

[f] Dipartimento di Scienze della Salute, Università "Magna Græcia" di Catanzaro, Viale Europa, 88100, Catanzaro, Italy

[g] National Center for Drug Research and Evaluation, Istituto Superiore di Sanità, Viale Regina Elena, 00161, Rome, Italy

[h] Department of Pharmacy and Pharmaceutical Technology and Physical-Chemistry, Faculty of Pharmacy and Food Sciences, University of Barcelona, Avda. Joan XXIII, 27-31 Barcelona, Catalonia, Spain

*For L.S.: phone, +39-06-49913737; email, luigi.scipione@uniroma1.it; for S.A.: phone, +39-0961-3694198; email, alcaro@unicz.it.

Table of contents

Dixon's plot of selected compounds towards *Ee*AChE and *eq*BChE (Figures S1-S12) S3-S8

IC_{50} graph of compound 20 towards *Ee*AChE (Figure S13) S9

Molecular docking studies (Figures S14-S17, Table S1) S10-S13

UV-Vis titrations spectra and Job's plots for selected compounds (Figures S18-S46) S14-S30

Dixon's plot of selected compounds towards *Ee*AChE and *eq*BChE

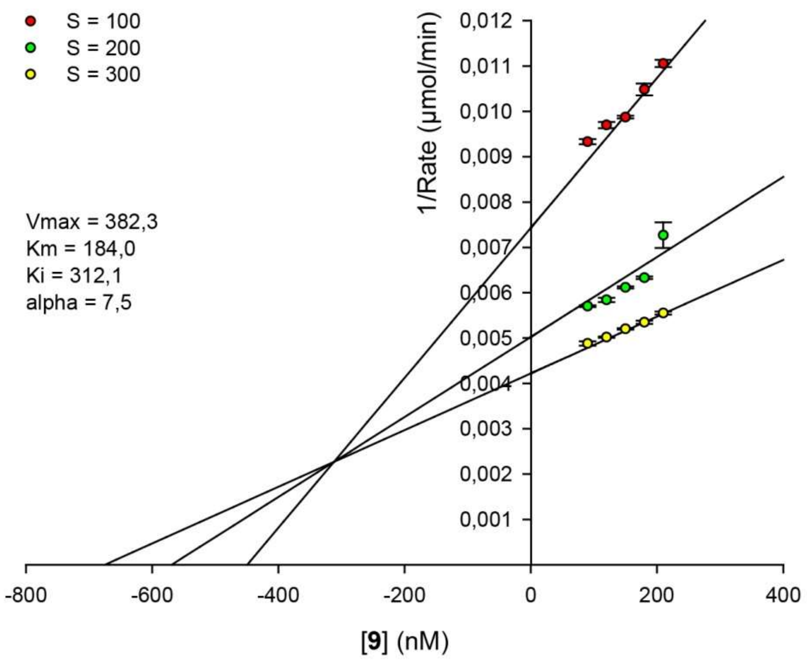

Figure S1. Dixon's plot obtained for **9** (90-210 nM), in presence of *Ee*AChE (0.0833 U/mL) and ASCh (100-300 µM). Mixed inhibition mechanism was observed (K_i = 0.312 ± 0.108 µM, R^2 = 0.982).

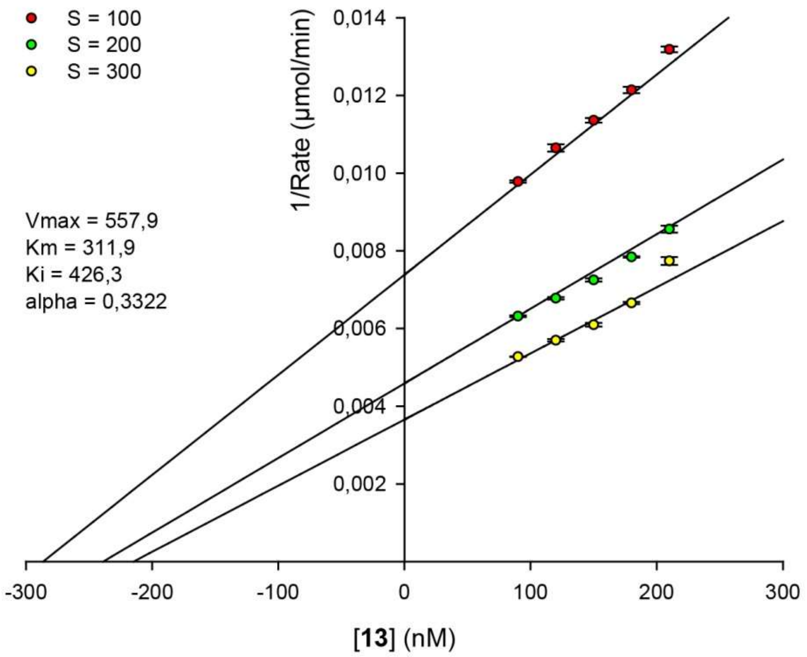

Figure S2. Dixon's plot obtained for **13** (90-210 nM), in presence of *Ee*AChE (0.0833 U/mL) and ASCh (100-300 µM). Mixed inhibition mechanism was observed (K_i = 0.426 ± 0.132 µM, R^2 = 0.991).

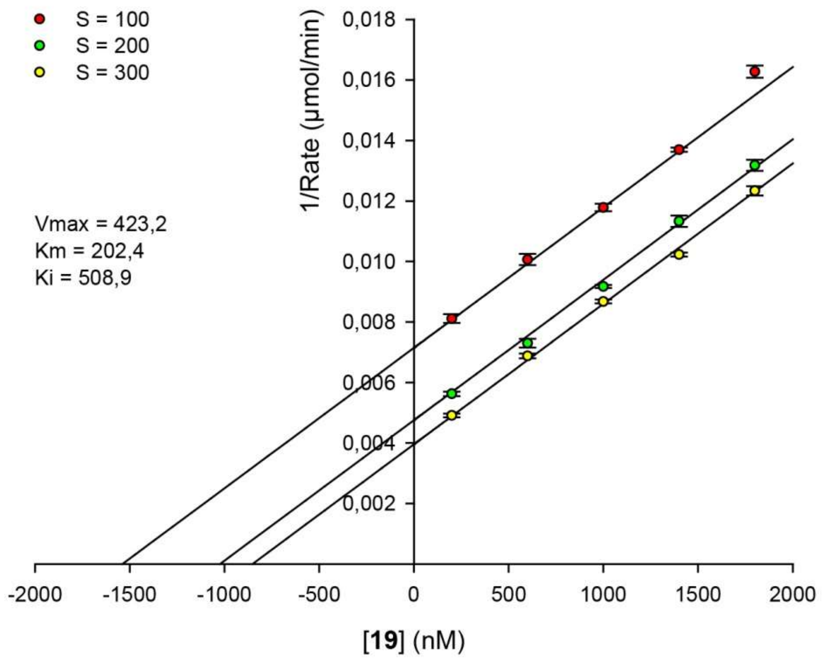

Figure S3. Dixon's plot obtained for **19** (200-1800 nM), in presence of *Ee*AChE (0.0833 U/mL) and ASCh (100-300 µM). Uncompetitive inhibition mechanism was observed ($K_i = 0.509 \pm 0.018$ µM, $R^2 = 0.992$).

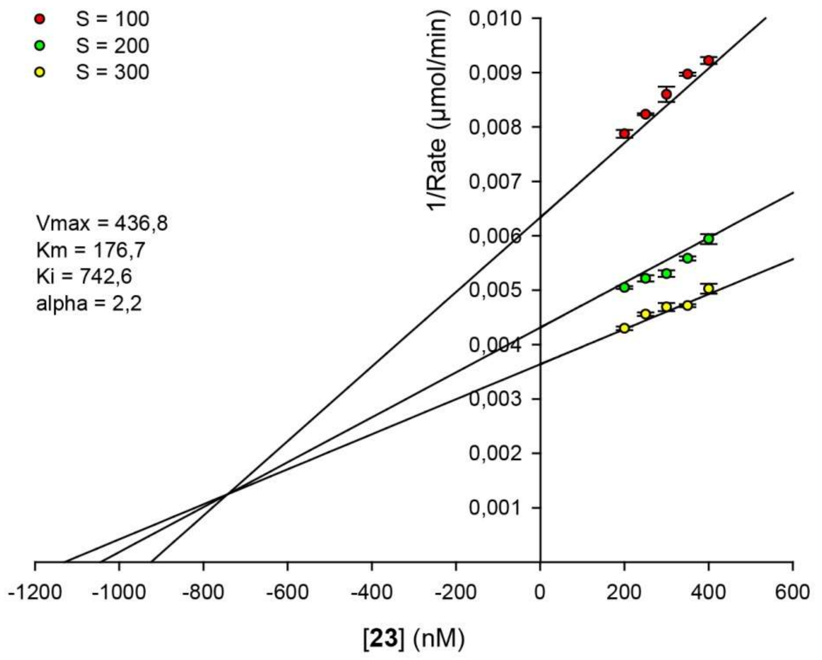

Figure S4. Dixon's plot obtained for **23** (200-400 nM), in presence of *Ee*AChE (0.0833 U/mL) and ASCh (100-300 µM). Mixed inhibition mechanism was observed ($K_i = 0.743 \pm 0.316$ µM, $R^2 = 0.983$).

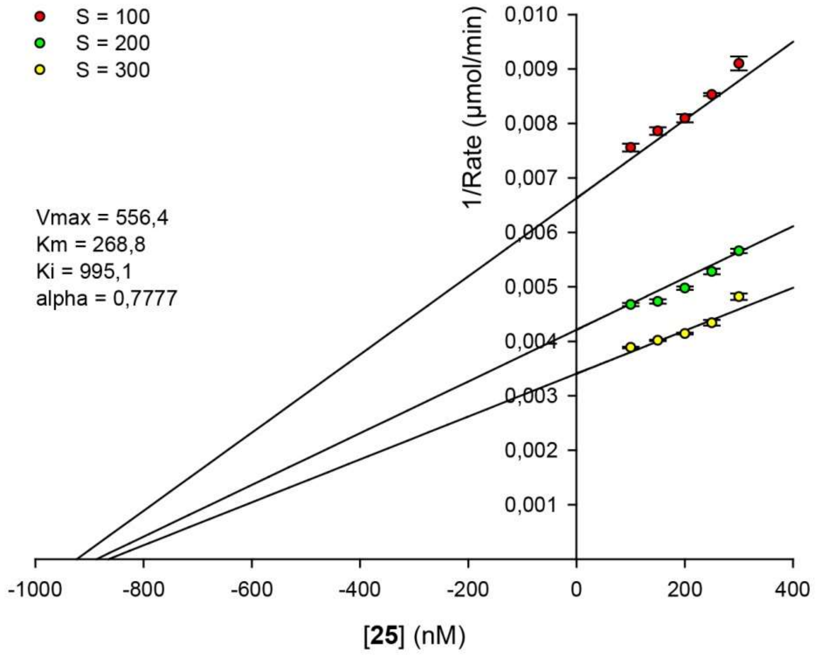

Figure S5. Dixon's plot obtained for **25** (100-300 nM), in presence of *Ee*AChE (0.0833 U/mL) and ASCh (100-300 µM). Mixed inhibition mechanism was observed ($K_i = 0.995 \pm 0.374$ µM, $R^2 = 0.988$).

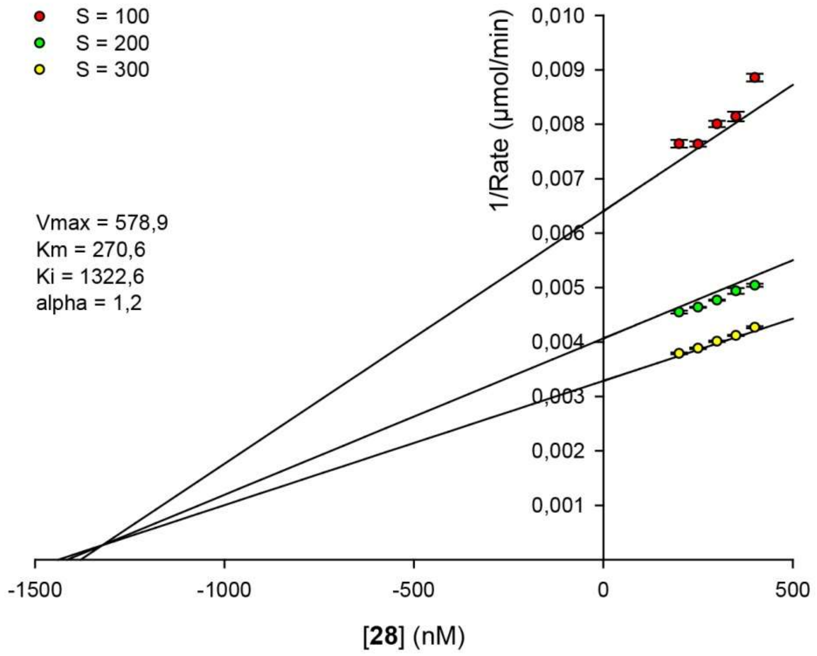

Figure S6. Dixon's plot obtained for **28** (200-400 nM), in presence of *Ee*AChE (0.0833 U/mL) and ASCh (100-300 µM). Mixed inhibition mechanism was observed ($K_i = 1.323 \pm 0.622$ µM, $R^2 = 0.990$).

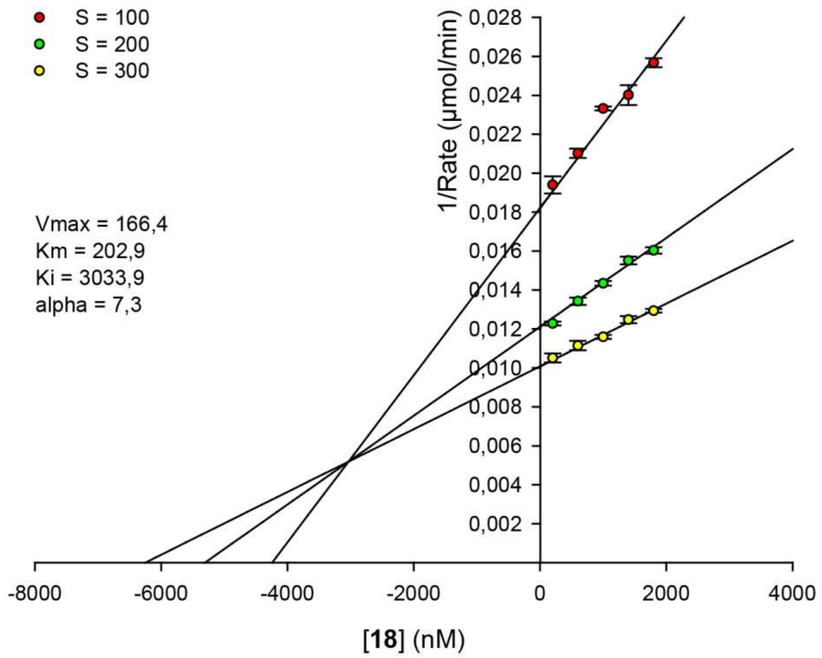

Figure S7. Dixon's plot obtained for **18** (200-1800 nM), in presence of *eq*BChE (0.0833 U/mL) and ASCh (100-300 μM). Mixed inhibition mechanism was observed (K_i = 3.034 ± 0.604 μM, R^2 = 0.986).

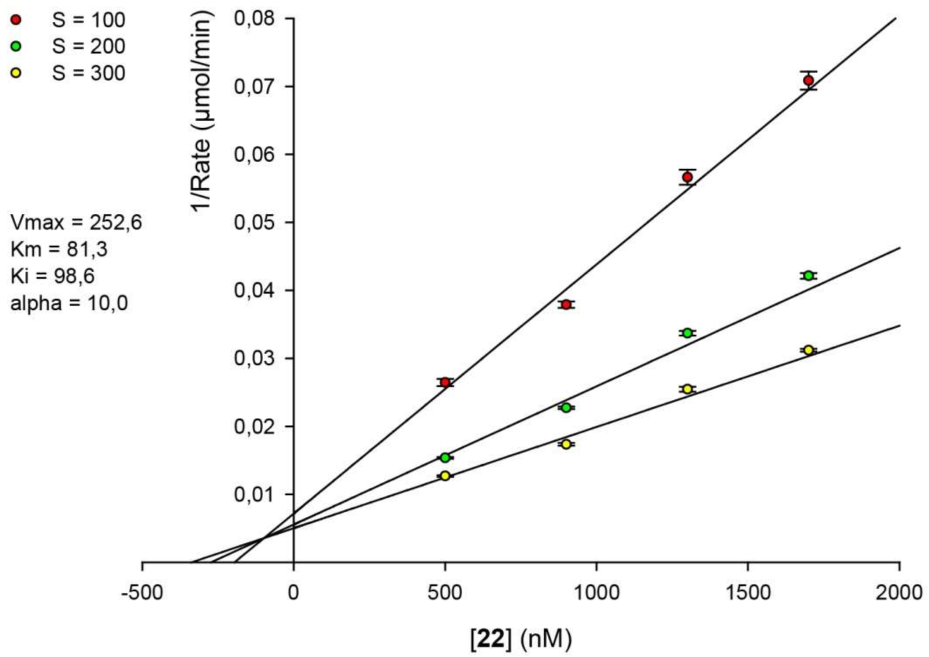

Figure S8. Dixon's plot obtained for **22** (500-1700 nM), in presence of *eq*BChE (0.0833 U/mL) and ASCh (100-300 μM). Mixed inhibition mechanism was observed (K_i = 0.099 ± 0.071 μM, R^2 = 0.990).

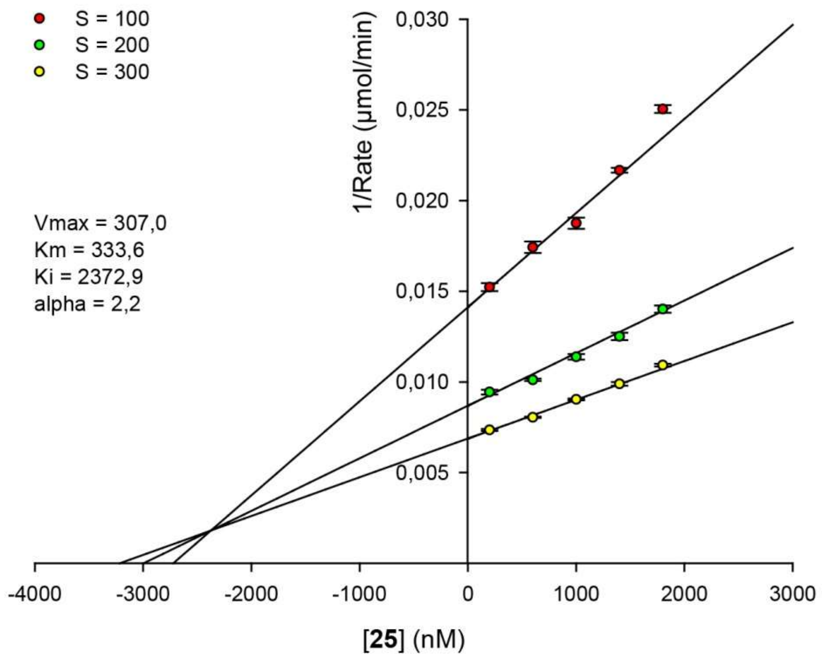

Figure S9. Dixon's plot obtained for **25** (200-1800 nM), in presence of *eq*BChE (0.0833 U/mL) and ASCh (100-300 µM). Mixed inhibition mechanism was observed (K_i = 2.373 ± 0.304 µM, R^2 = 0.992).

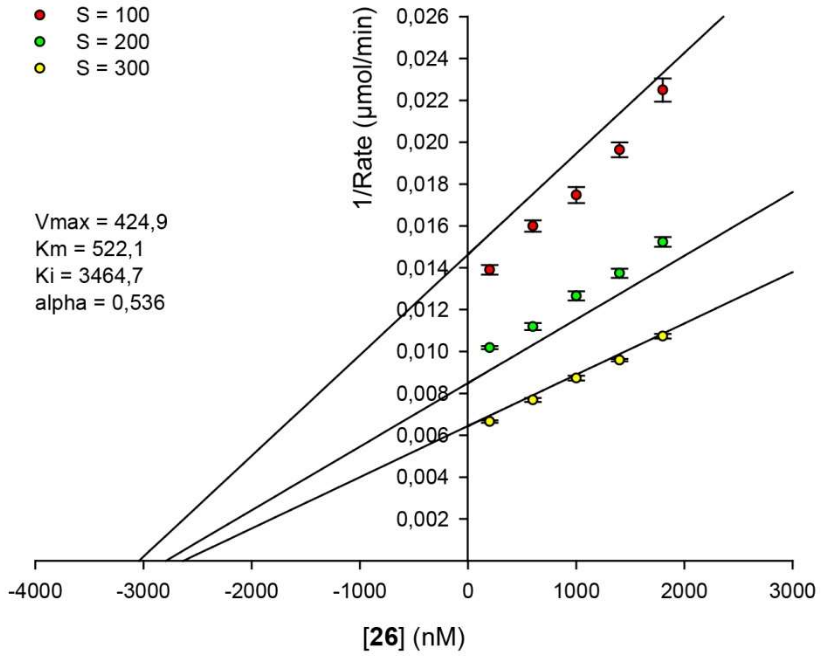

Figure S10. Dixon's plot obtained for **26** (200-1800 nM), in presence of *eq*BChE (0.0833 U/mL) and ASCh (100-300 µM). Mixed inhibition mechanism was observed (K_i = 3.465 ± 1.480 µM, R^2 = 0.950).

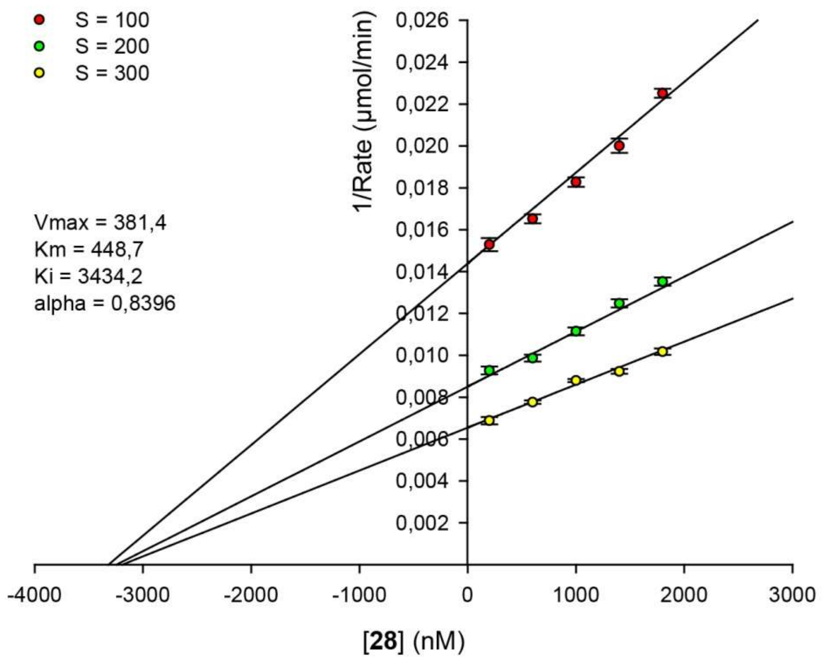

Figure S11. Dixon's plot obtained for **28** (200-1800 nM), in presence of *eq*BChE (0.0833 U/mL) and ASCh (100-300 µM). Mixed inhibition mechanism was observed (K_i = 3.434 ± 0.701 µM, R^2 = 0.988).

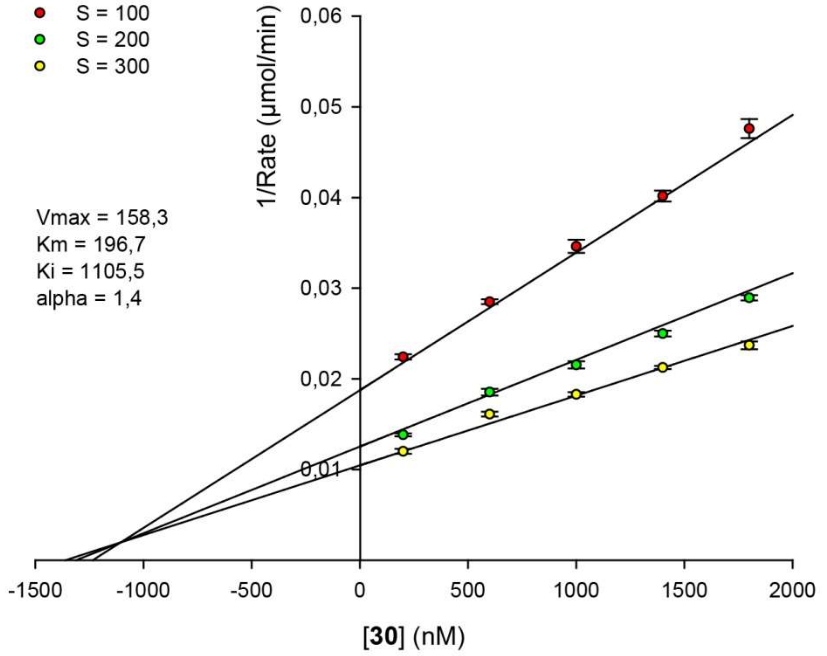

Figure S12. Dixon's plot obtained for **30** (200-1800 nM), in presence of *eq*BChE (0.0833 U/mL) and ASCh (100-300 µM). Mixed inhibition mechanism was observed (K_i = 1.105 ± 0.189 µM, R^2 = 0.983).

IC_{50} graph of compound 20 towards *Ee*AChE

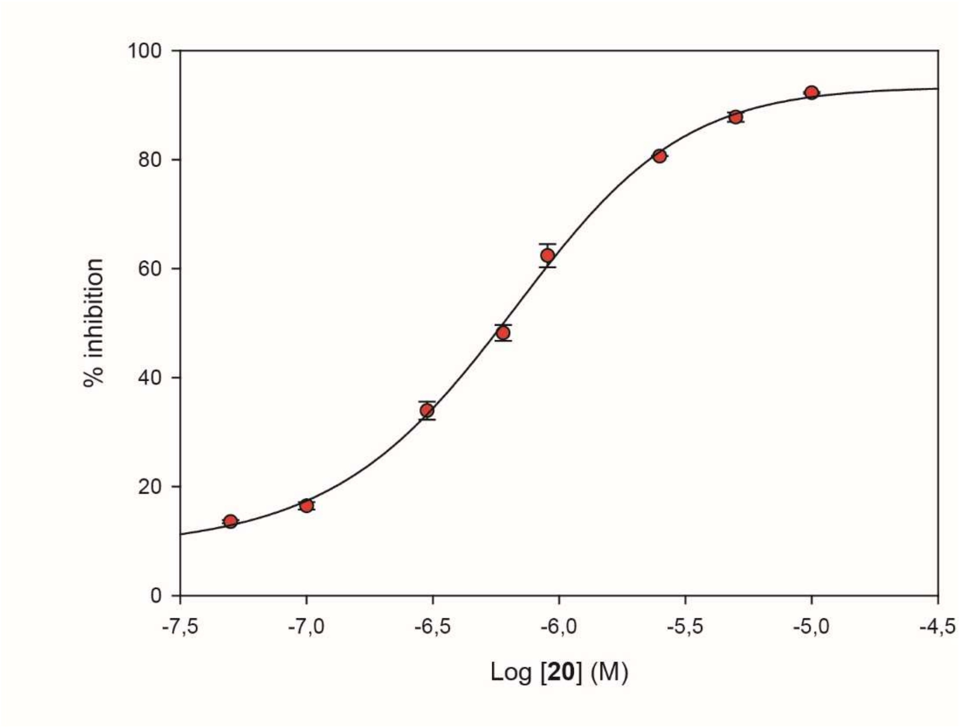

Figure S13. IC_{50} graph for compound **20** (0.05-10 µM), in presence of *Ee*AChE (0.0833 U/mL) and ASCh (100 µM). The IC_{50} value extrapolated from the graph is 622 ± 30 nM**.**

Molecular docking studies

a)

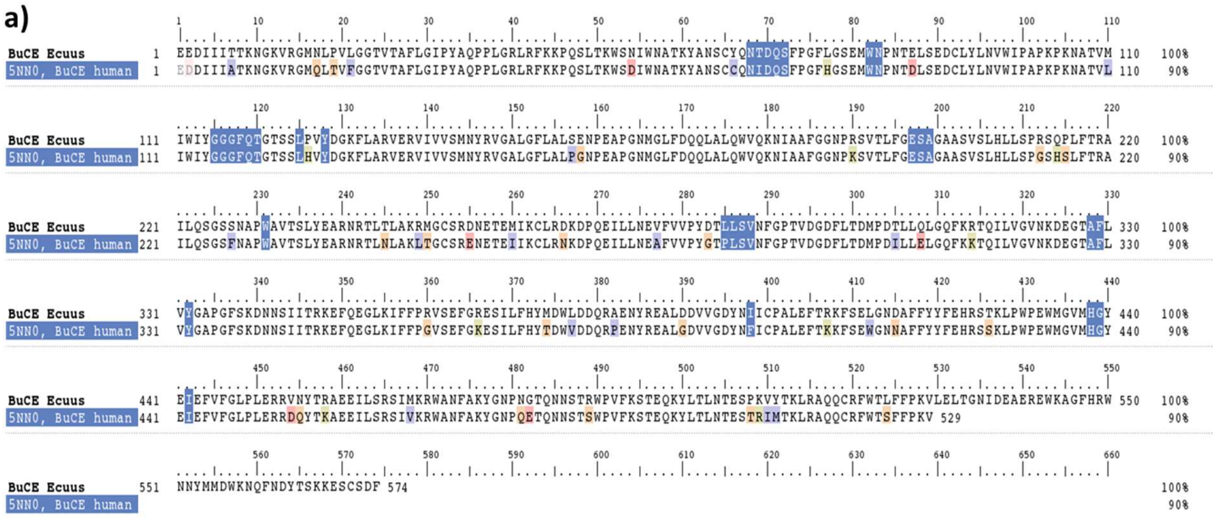

b)

Distance (Å)	Identity (%)	Similarity (%)	Homology (%)
3	90	90	90
4	90	90	90
5	90	90	90
6	90	90	90
7	90	90	90

Figure S14. a) Sequence alignment of equine BChE (entry BuCE_Ecuus, P81908) with human BChE (PDB code: 5NN0) generated by ClustalW algorithm. Among the sequence, only the different residues are coloured. The residues of the binding site are evidenced in blue. **b)** The percentage identity, similarity and homology of the aligned sequences to the selected model within a range of distances from the binding site are reported.

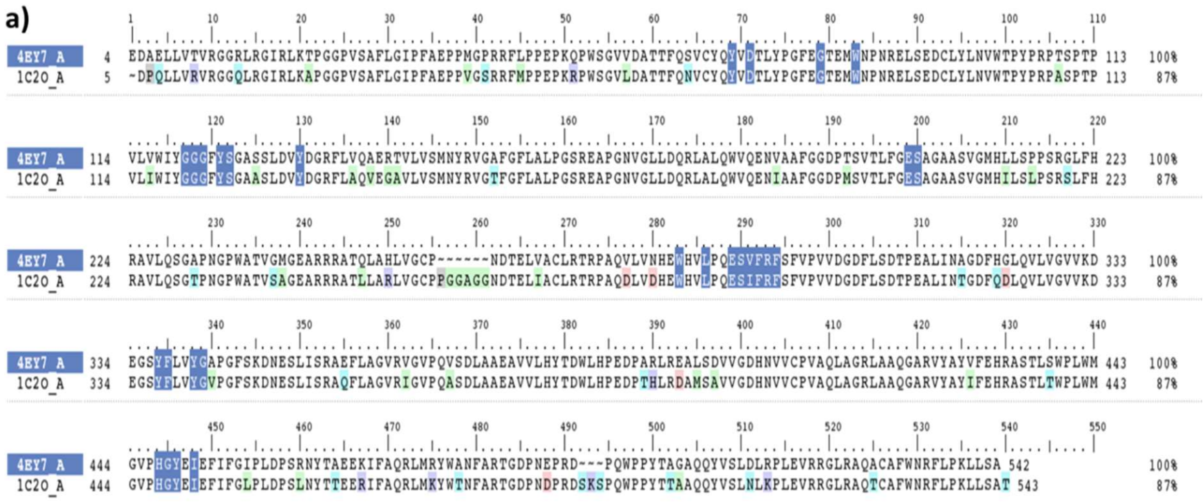

b)

Distance (Å)	Identity (%)	Similarity (%)	Homology (%)
3	96.4	100	100
4	96.4	98.2	100
5	95.2	98.4	100
6	96.1	98.7	100
7	94.7	98.9	98.9

Figure S15. a) Sequence alignment of *Electrophorus electricus* AChE (PDB code: 1C2O) with human AChE (PDB code: 4EY7) generated by ClustalW algorithm. Among the sequence, only the different residues are coloured. The residues of the binding site are evidenced in blue. **b)** The percentage identity, similarity and homology of the aligned sequences to the selected model within a range of distances from the binding site are reported.

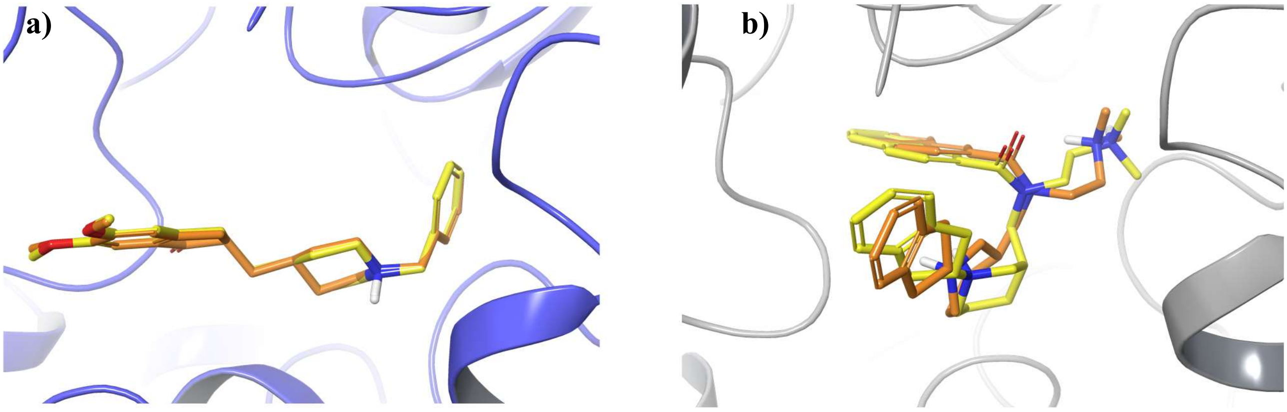

Figure S16. Superimposition of the re-docked (yellow carbons) and co-crystallized (orange carbons) ligand binding modes, into **a)** *h*AChE and **b)** *h*BChE active sites. RMSD was found to be 0.12 Å for *h*AChE ligand and 0.96 Å for *h*BChE ligand.

Table S1. Glide Score values (kcal/mol) of compounds **9-33** for binding *h*AChE and *h*BChE and re-docking values of co-crystallized ligands of 4EY7 and 5NN0 PDB structures.

**Compound**	**Glide Score**	
	***h*AChE**	***h*BChE**
**9**	-7.81	-5.59
**10**	-9.63	-8.44
**11**	-9.35	-7.90
**12**	-10.47	-8.21
**13**	-7.05	-5.67
**14**	-10.12	-8.12
**15**	-10.24	-8.20
**16**	-9.57	-7.13
**17**	-9.83	-6.85
**18**	-11.60	-7.67
**19**	-10.55	-7.21
**20**	-11.33	-7.31
**21**	-10.40	-6.52
**22**	-10.91	-8.04
**23**	-7.78	-5.13
**24**	-9.41	-6.89
**25**	-7.30	-5.51
**26**	-10.66	-8.19
**27**	-10.37	-8.31
**28**	-9.64	-7.46
**29**	-10.70	-6.90
**30**	-11.18	-7.90
**31**	-10.52	-7.61
**32**	-10.96	-7.02
**33**	-9.82	-7.39
4EY7 x-ray ligand	-12.70	-
5NN0 x-ray ligand	-	-9.96

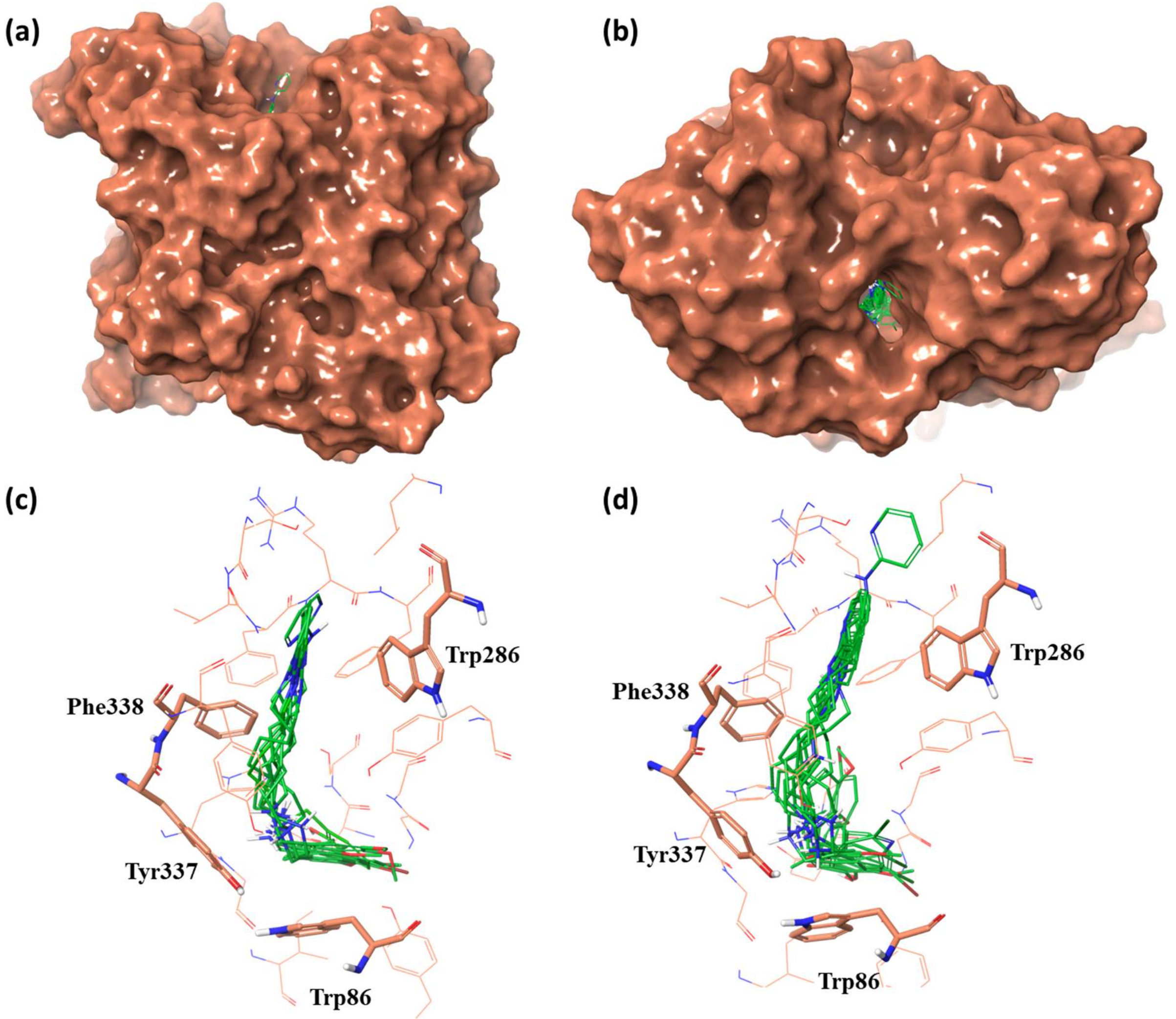

Figure S17. a) Front view and **b)** top view of the best docked poses of compounds **9-33** and donepezil into *h*AChE. Overlay of donepezil and the best docked poses of **c)** pyrimidine and **d)** pyridine derivatives (green carbon sticks) in the active site of *h*AChE. Trp86 (CAS site), Trp286 (PAS site), Tyr337 and Phe338 (mid-gorge region) residues of the *h*AChE pocket were shown as salmon carbon sticks in the receptor.

UV-Vis titrations spectra and Job's plots for selected compounds

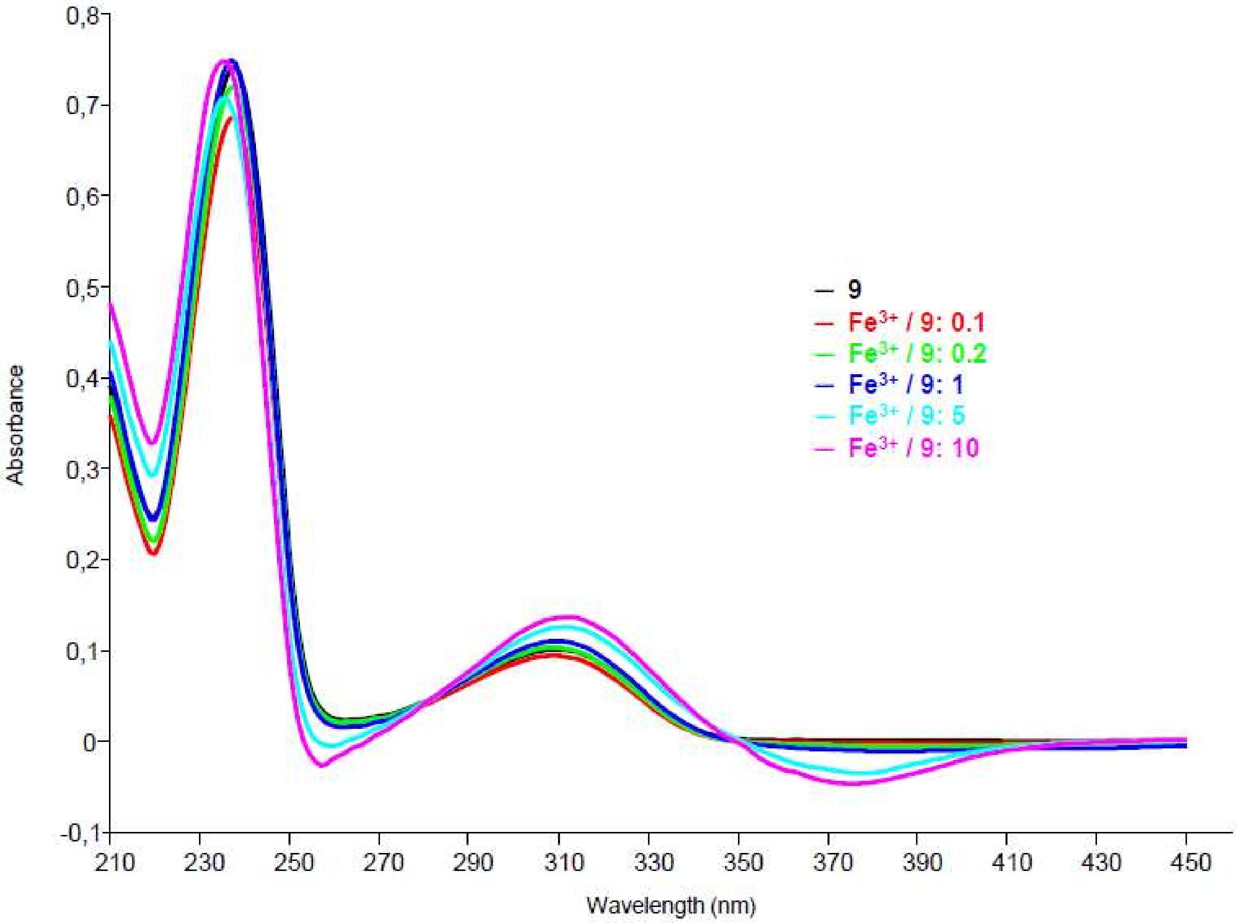

Figure S18. UV-Vis titration of ligand **9** with Fe^{3+}. The greatest variations in spectra with increasing amount of metal are observed at 220 and 312 nm, with an increase of absorbance, and at 256 and 372 nm, with a reduction of absorbance. There are two isosbestic points at 281 and 349 nm.

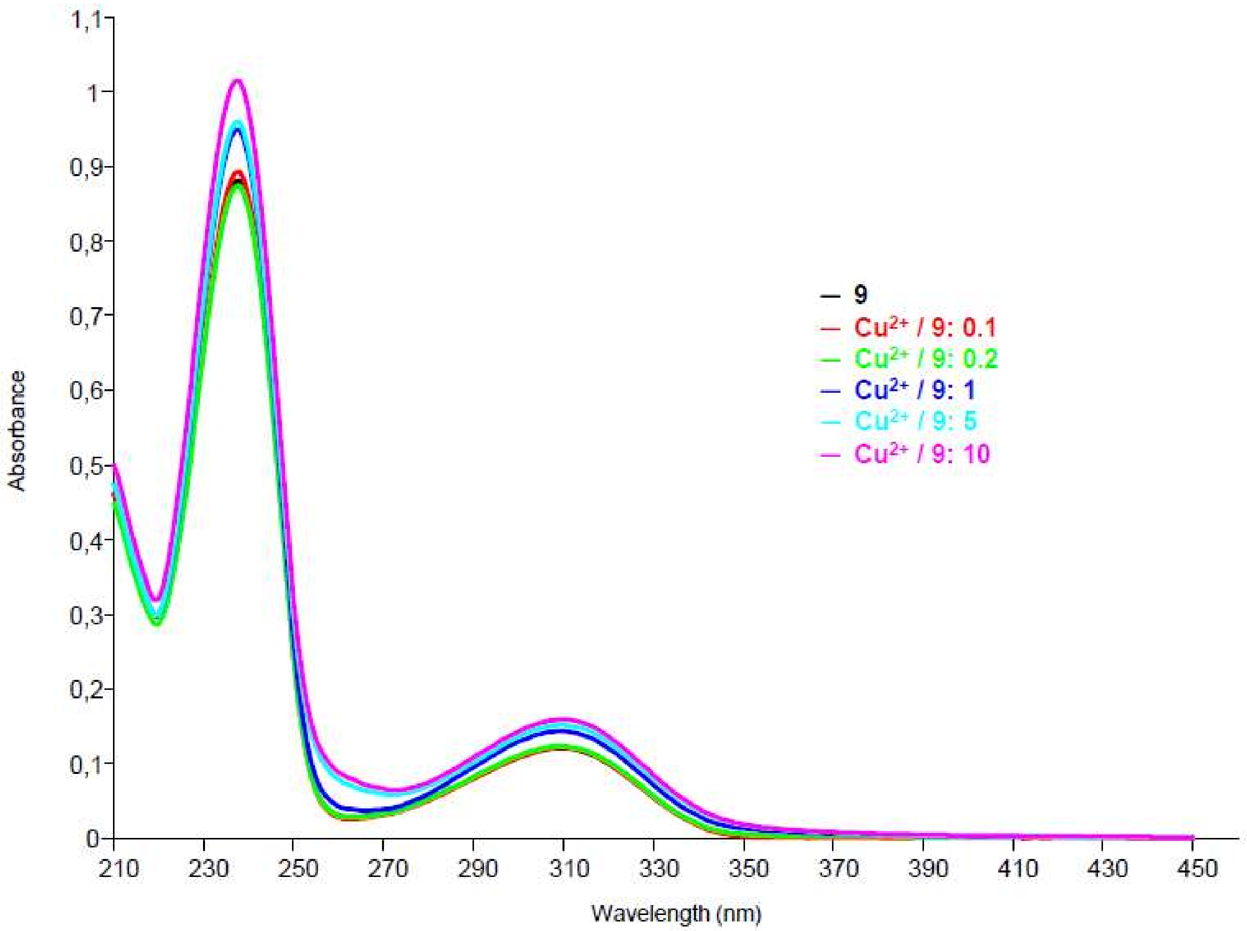

Figure S19. UV-Vis titration of ligand **9** with Cu^{2+}. The greatest variations in spectra with increasing amount of metal are observed at 237, 260 and 310 nm, with an increase of absorbance.

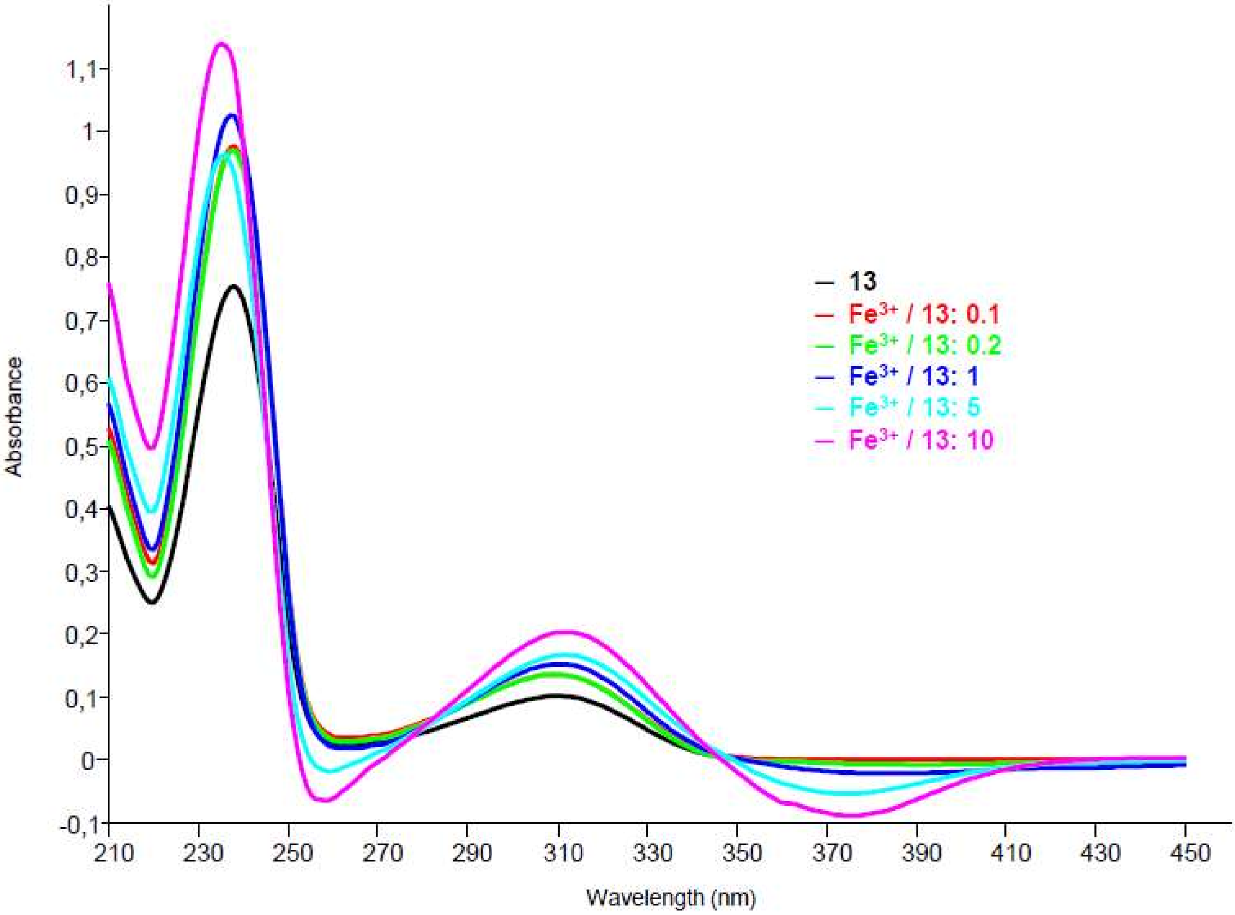

Figure S20. UV-Vis titration of ligand **13** with Fe^{3+}. The greatest variations in spectra with increasing amount of metal are observed at 220 and 312 nm, with an increase of absorbance, and at 256 and 372 nm, with a reduction of absorbance. There are two isosbestic points at 278 and 345 nm.

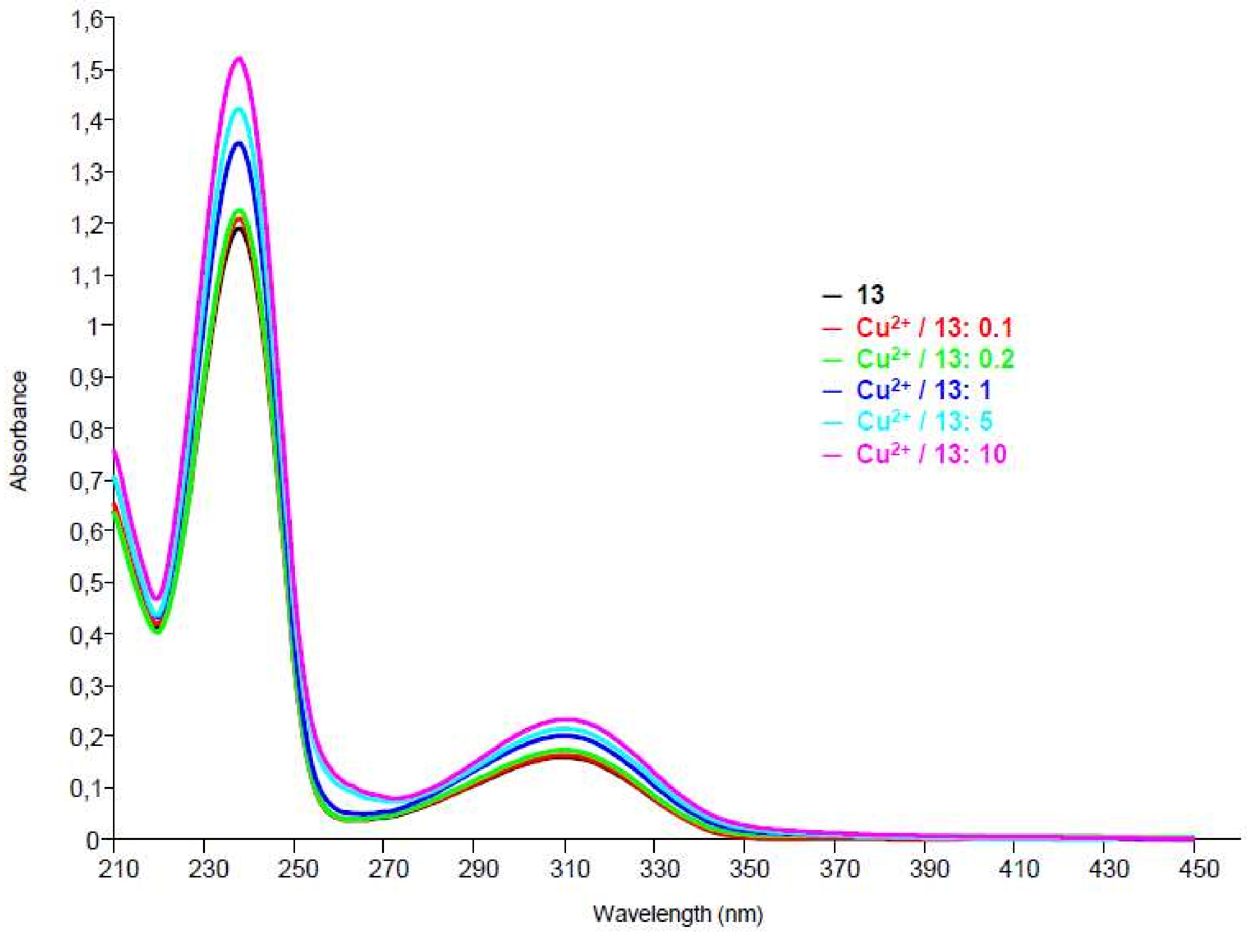

Figure S21. UV-Vis titration of ligand **13** with Cu^{2+}. The greatest variations in spectra with increasing amount of metal are observed at 238, 259 and 310 nm, with an increase of absorbance.

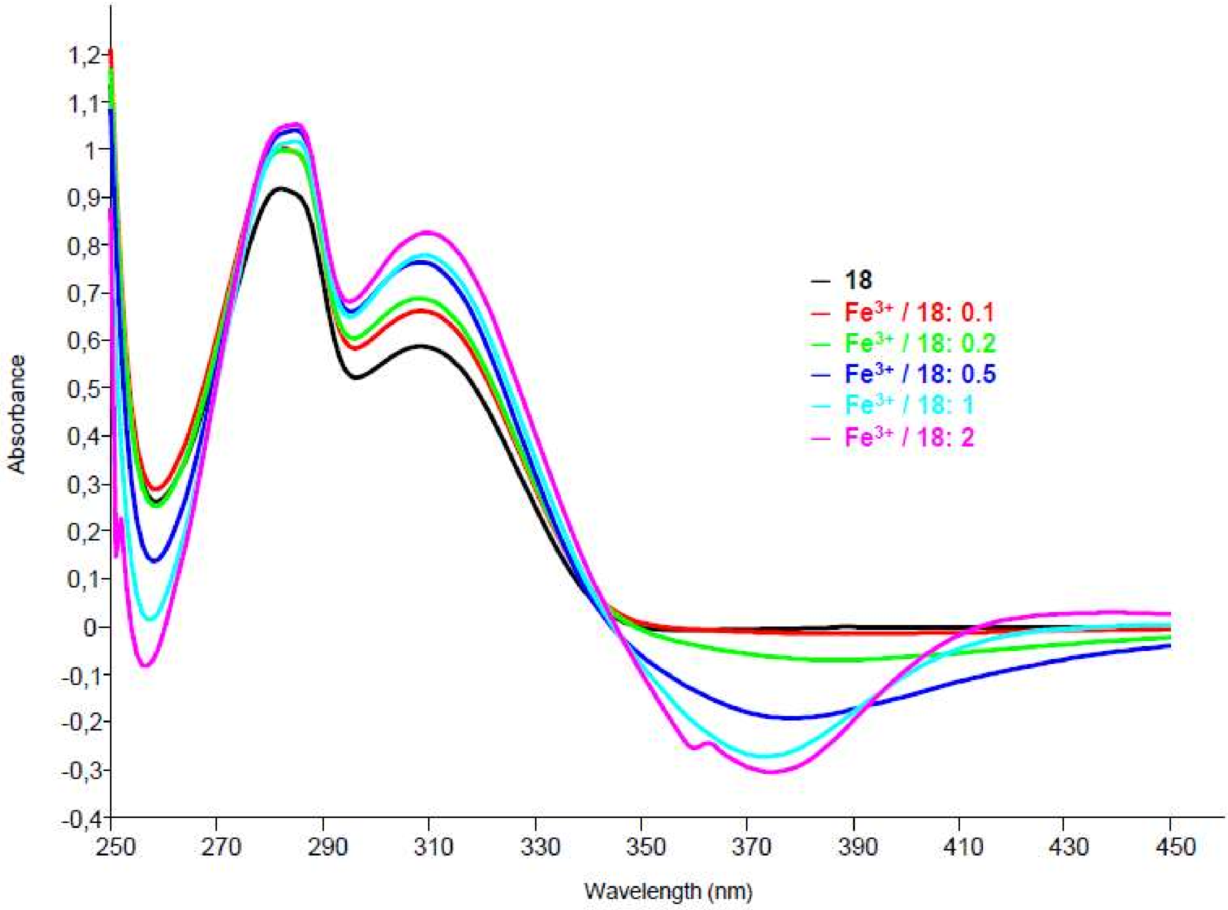

Figure S22. UV-Vis titration of ligand **18** with Fe^{3+}. The greatest variations in spectra with increasing amount of metal are observed at 258 and 375 nm, with a reduction of absorbance, and at 280 and 309 nm, with an increase of absorbance. There are two isosbestic points at 273 and 343 nm.

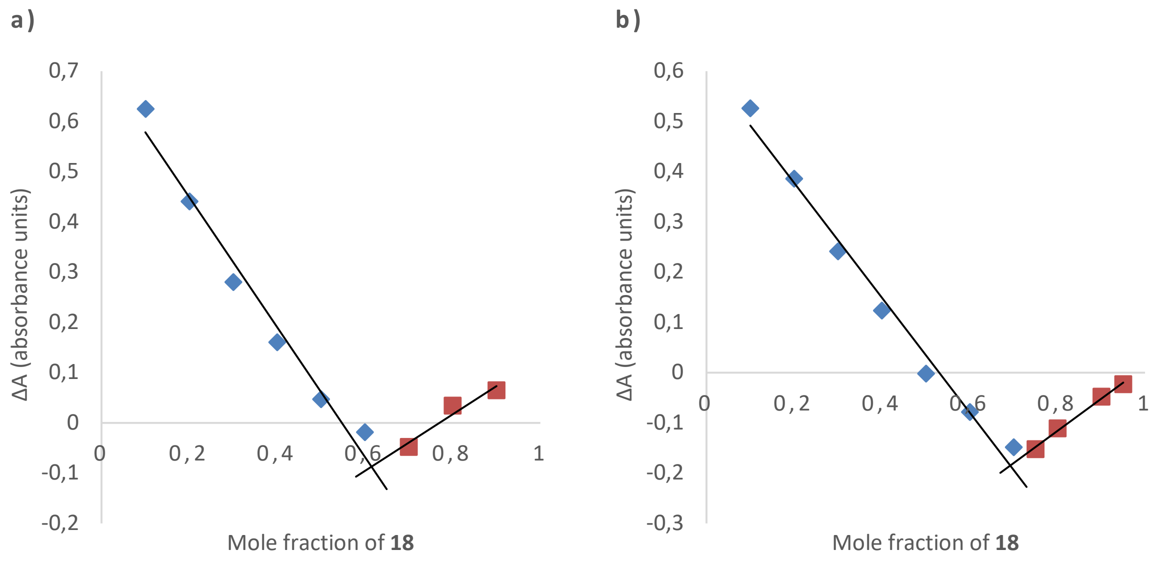

Figure S23. Job's plot of compound **18** in presence of Fe^{3+}: variation of the absorbance (ΔA) at the wavelength of 258 nm in **a)** or 375 nm in **b)**, in ordinate, versus the mole fraction of **18**, in abscissa. X (mole fraction that causes the maximum variation of absorbance) = 0.69; n (number of ligand molecules per cation) = 2.

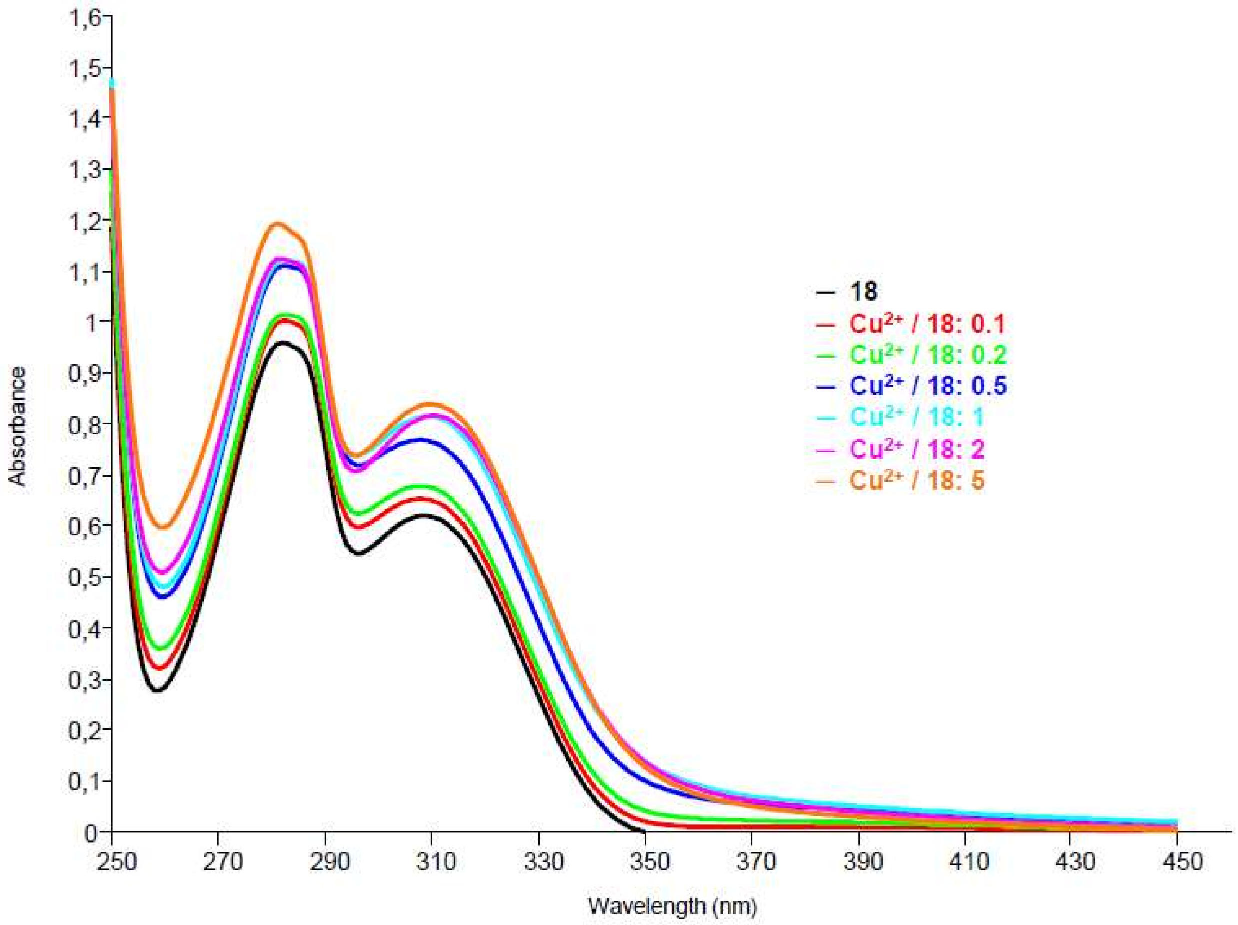

Figure S24. UV-Vis titration of ligand **18** with Cu^{2+}. The greatest variations in spectra with increasing amount of metal are observed at 258, 283 and 309 nm, with an increase of absorbance.

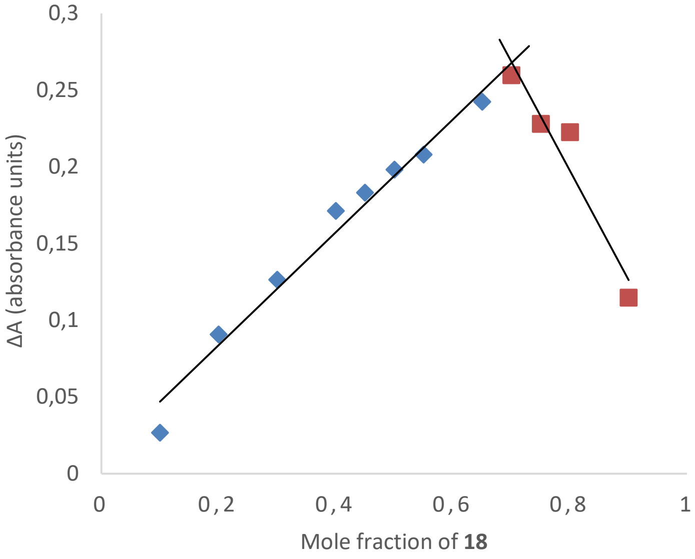

Figure S25. Job's plot of compound **18** in presence of Cu^{2+}: variation of the absorbance (ΔA) at the wavelength of 330 nm, in ordinate, versus the mole fraction of **18**, in abscissa. X (mole fraction that causes the maximum variation of absorbance) = 0.70; n (number of ligand molecules per cation) = 2.

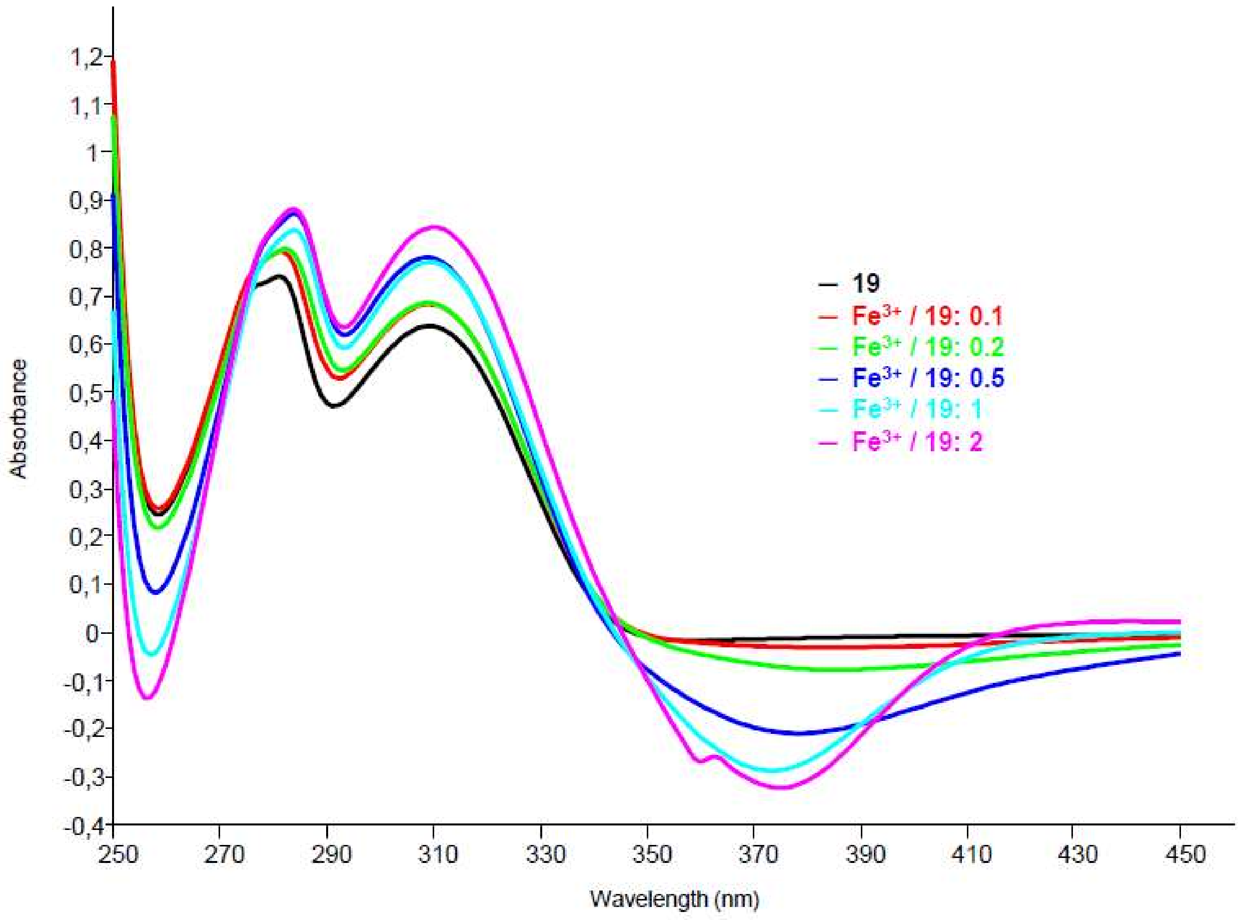

Figure S26. UV-Vis titration of ligand **19** with Fe^{3+}. The greatest variations in spectra with increasing amount of metal are observed at 259 and 375 nm, with a reduction of absorbance, and at 284 and 312 nm, with an increase of absorbance. There are two isosbestic points at 275 and 343 nm.

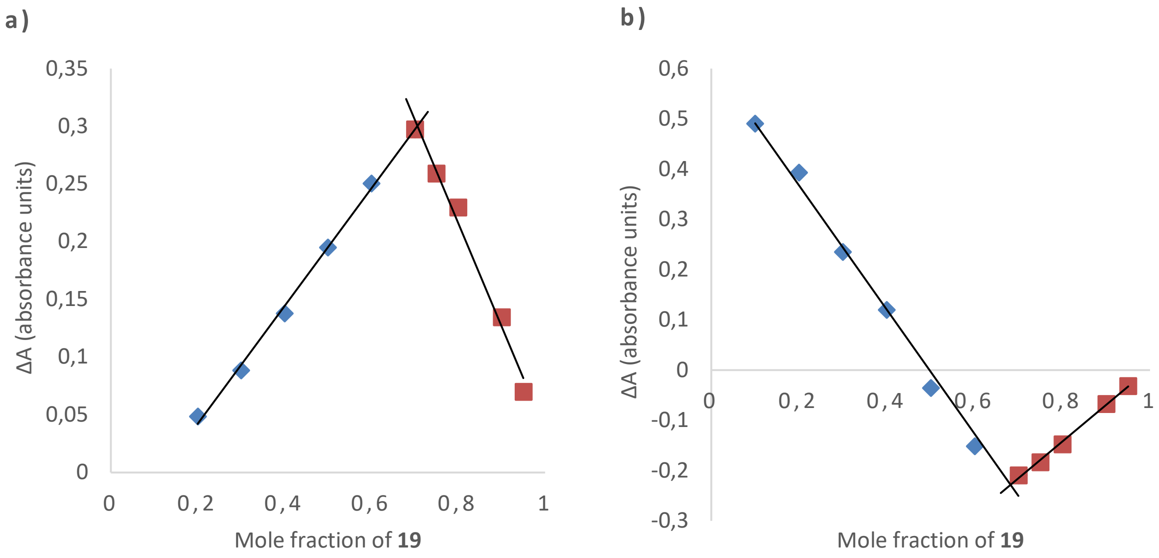

Figure S27. Job's plot of compound **19** in presence of Fe^{3+}: variation of the absorbance (ΔA) at the wavelength of 292 nm in **a)** or at 375 nm in **b)**, in ordinate, versus the mole fraction of **19**, in abscissa. X (mole fraction that causes the maximum variation of absorbance) = 0.71 in **a)**, 0.68 in **b)**; n (number of ligand molecules per cation) = 2.

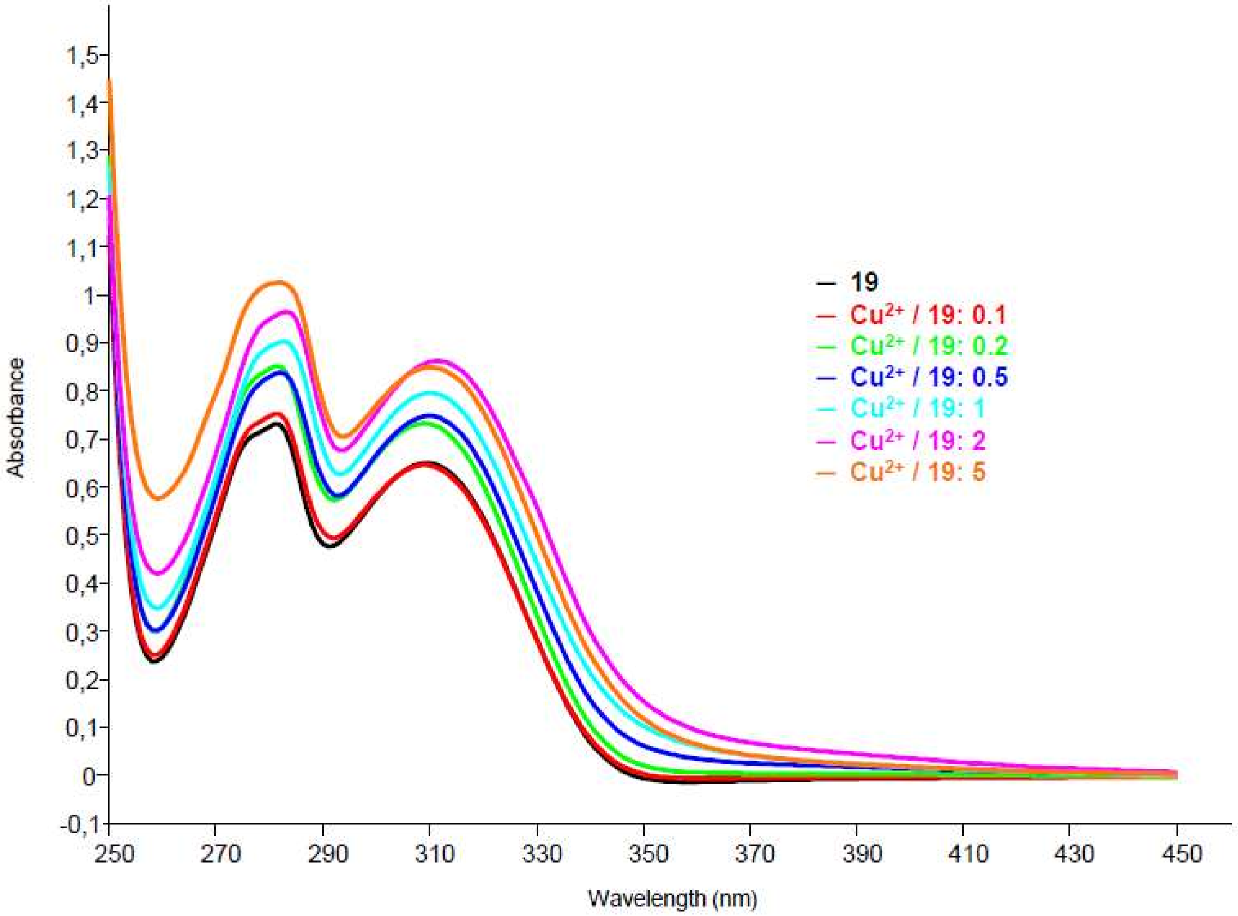

Figure S28. UV-Vis titration of ligand **19** with Cu^{2+}. The greatest variations in spectra with increasing amount of metal are observed at 259, 280 and 312 nm, with an increase of absorbance.

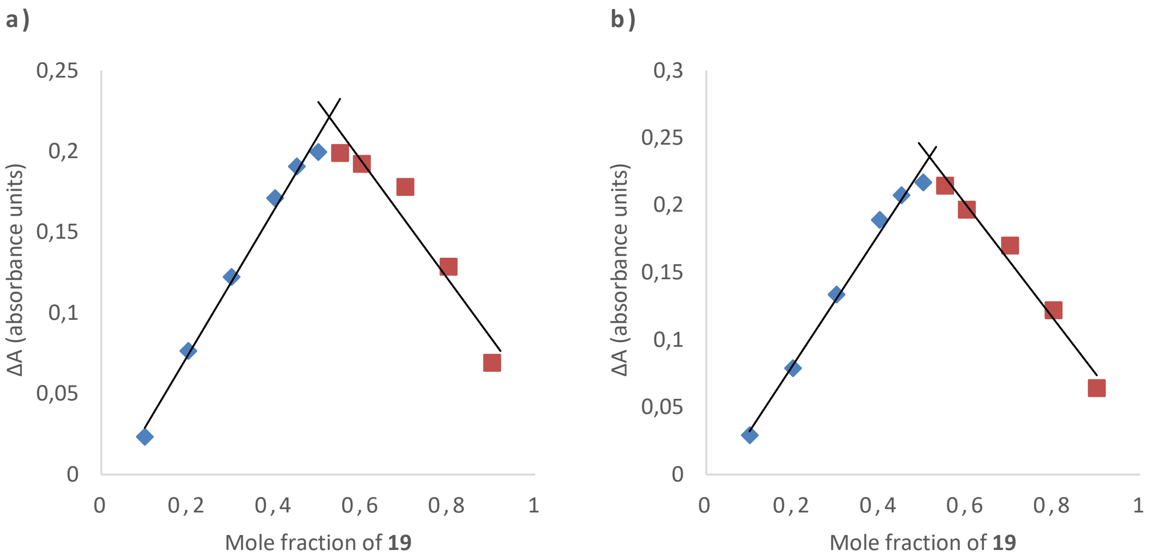

Figure S29. Job's plot of compound **19** in presence of Cu^{2+}: variation of the absorbance (ΔA) at the wavelength of 312 nm in **a)** or at 330 nm in **b)**, in ordinate, versus the mole fraction of **19**, in abscissa. X (mole fraction that causes the maximum variation of absorbance) = 0.53 in **a)**, 0.51 in **b)**; n (number of ligand molecules per cation) = 1.

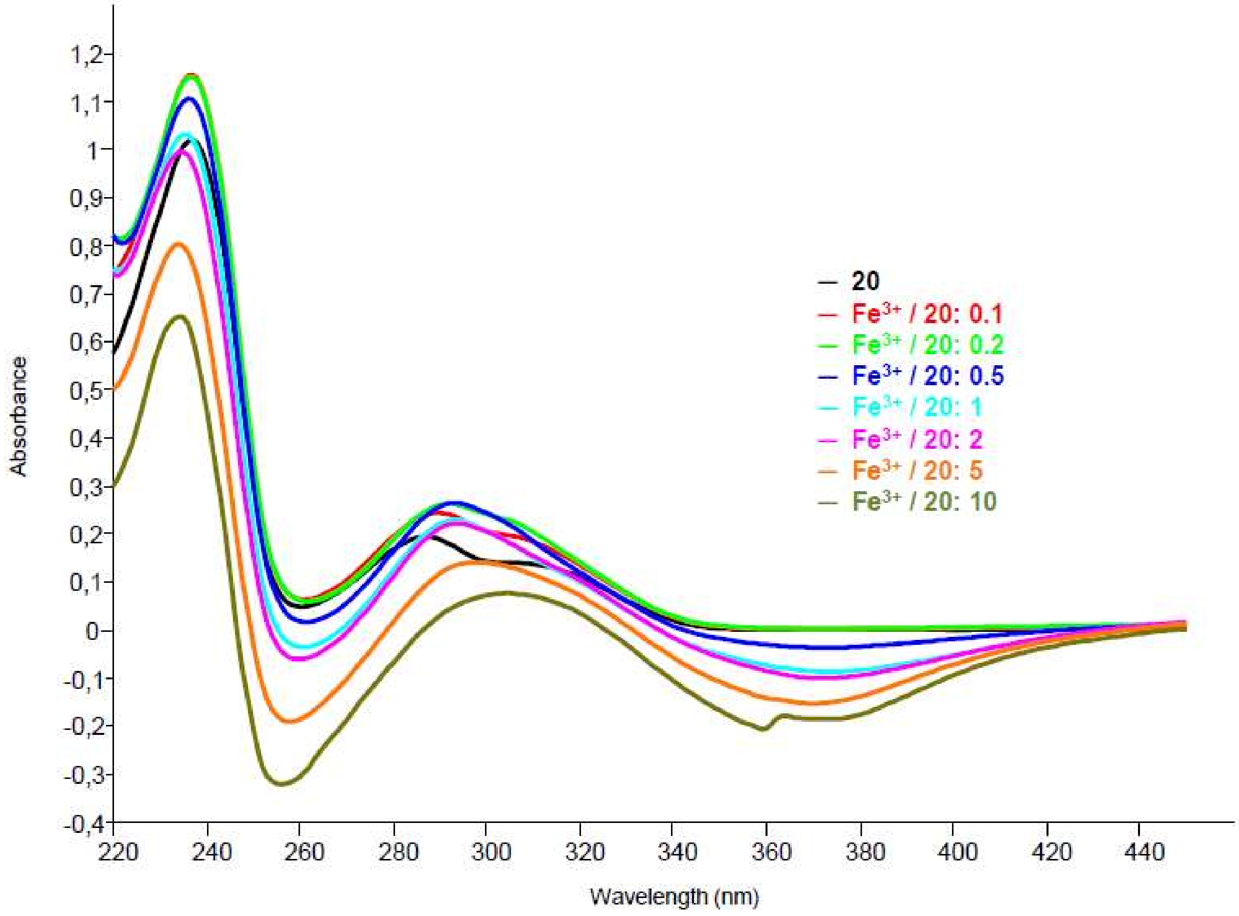

Figure S30. UV-Vis titration of ligand **20** with Fe^{3+}. The greatest variations in spectra with increasing amount of metal are observed at 256 and 370 nm, with a regular reduction of absorbance, at 234 nm and between 270-330 nm.

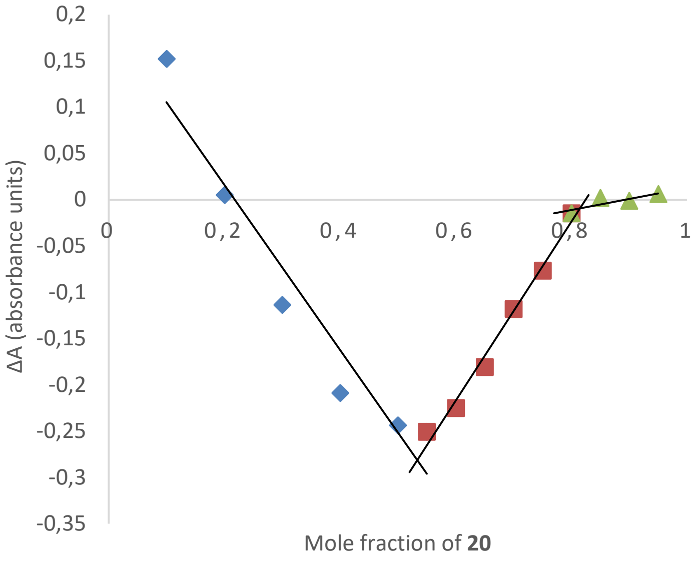

Figure S31. Job's plot of compound **20** in presence of Fe^{3+}: variation of the absorbance (ΔA) at the wavelength of 370 nm, in ordinate, versus the mole fraction of **20**, in abscissa. X_1 (mole fraction that causes the maximum variation of absorbance) = 0.53; X_2 = 0.80; n_1 (number of ligand molecules per cation) = 1; n_2 = 4.

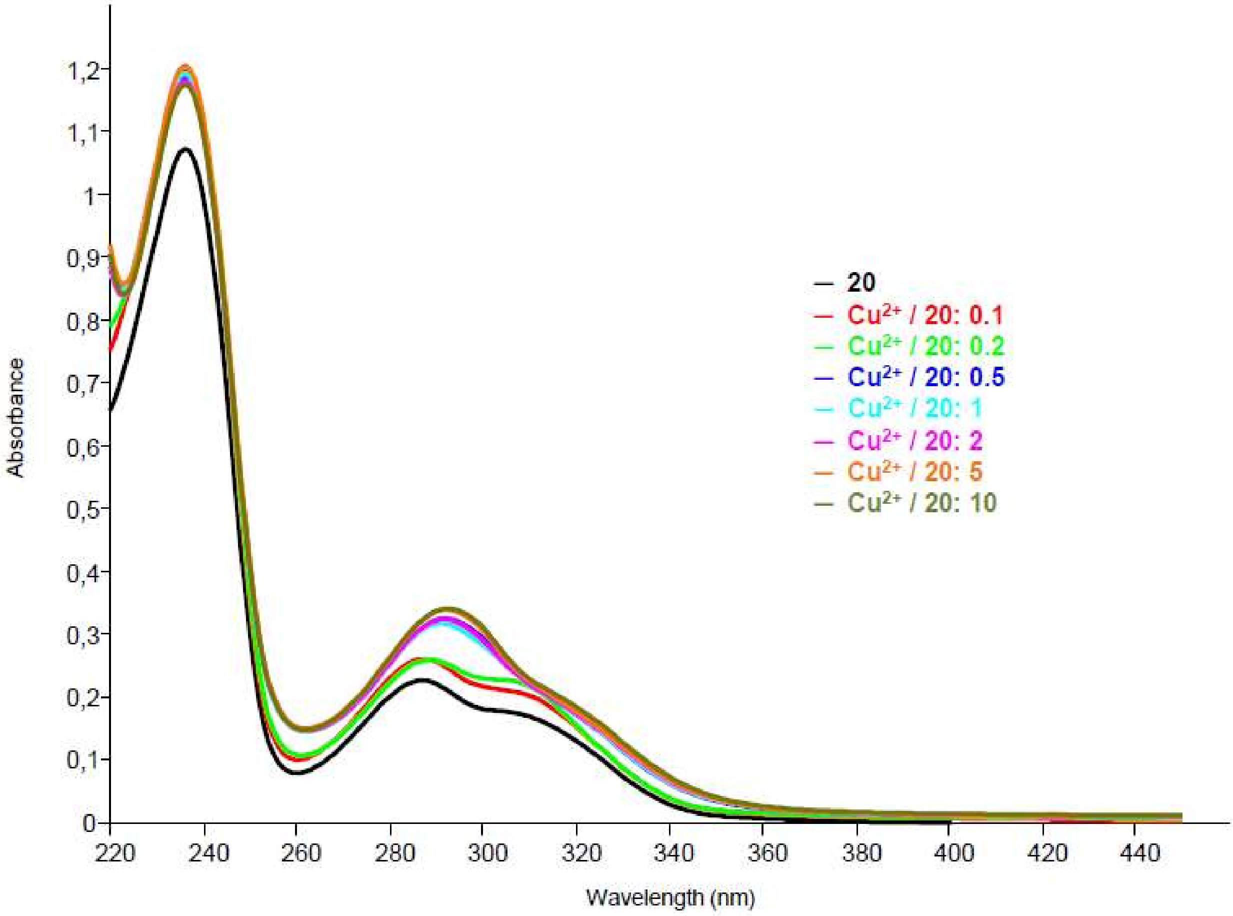

Figure S32. UV-Vis titration of ligand **20** with Cu^{2+}. The greatest variations in spectra with increasing amount of metal are observed at 260, 294 and 308 nm, with an increase of absorbance.

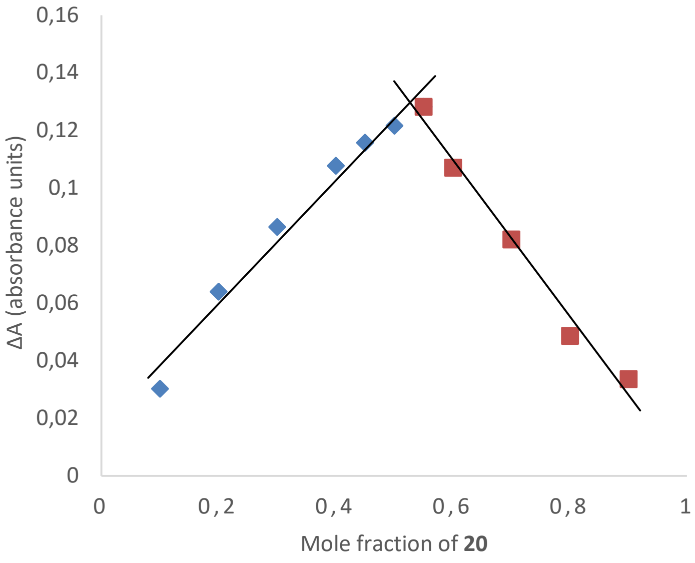

Figure S33. Job's plot of compound **20** in presence of Cu^{2+}: variation of the absorbance (ΔA) at the wavelength of 330 nm, in ordinate, versus the mole fraction of **20**, in abscissa. X (mole fraction that causes the maximum variation of absorbance) = 0.53; n (number of ligand molecules per cation) = 1.

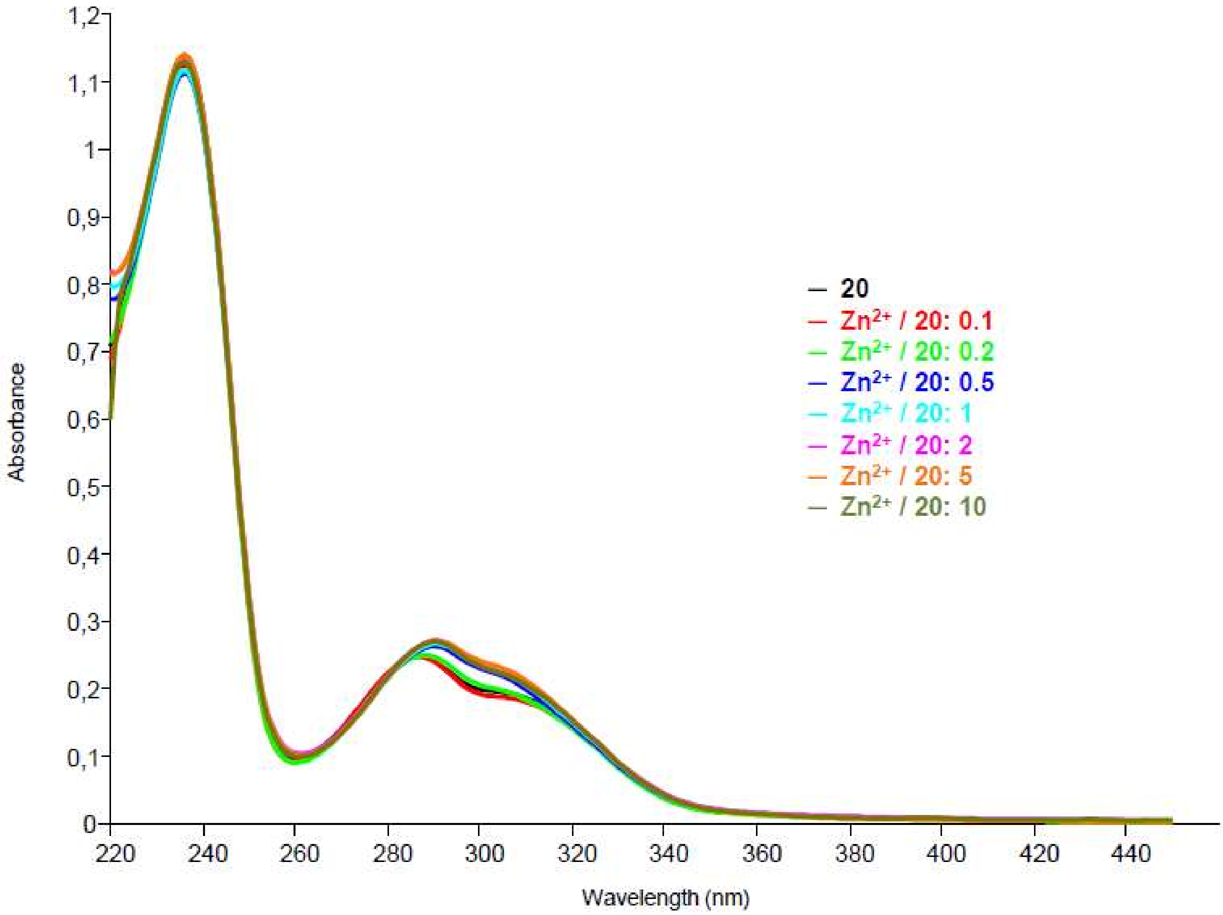

Figure S34. UV-Vis titration of ligand **20** with Zn^{2+}. The variations in spectra with increasing amount of metal are observed only between 285-314 nm, with an increase of absorbance.

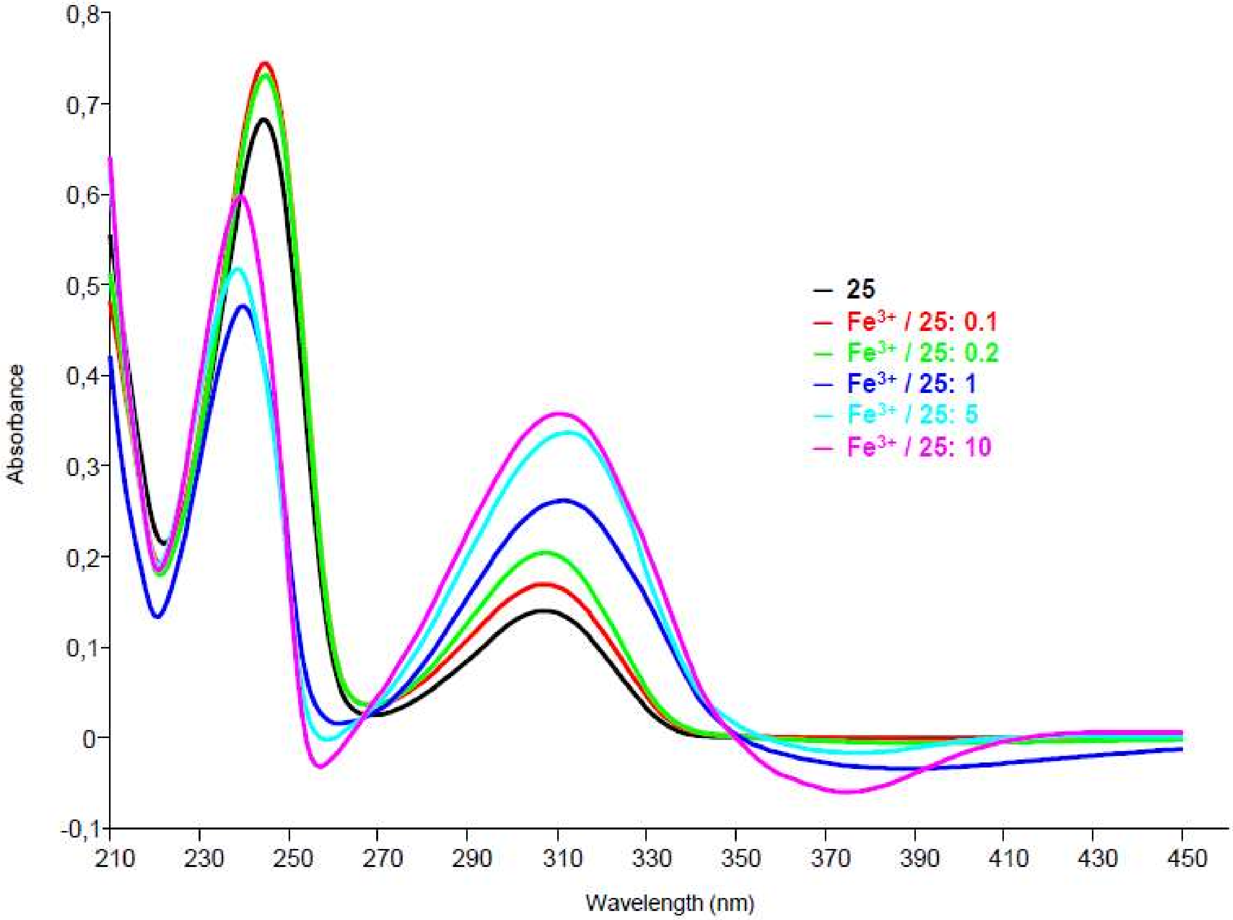

Figure S35. UV-Vis titration of ligand **25** with Fe^{3+}. The greatest variations in spectra with increasing amount of metal are observed at 251 and 374 nm, with a reduction of absorbance, and at 318 nm, with an increase of absorbance. There are two isosbestic points at 270 and 350 nm.

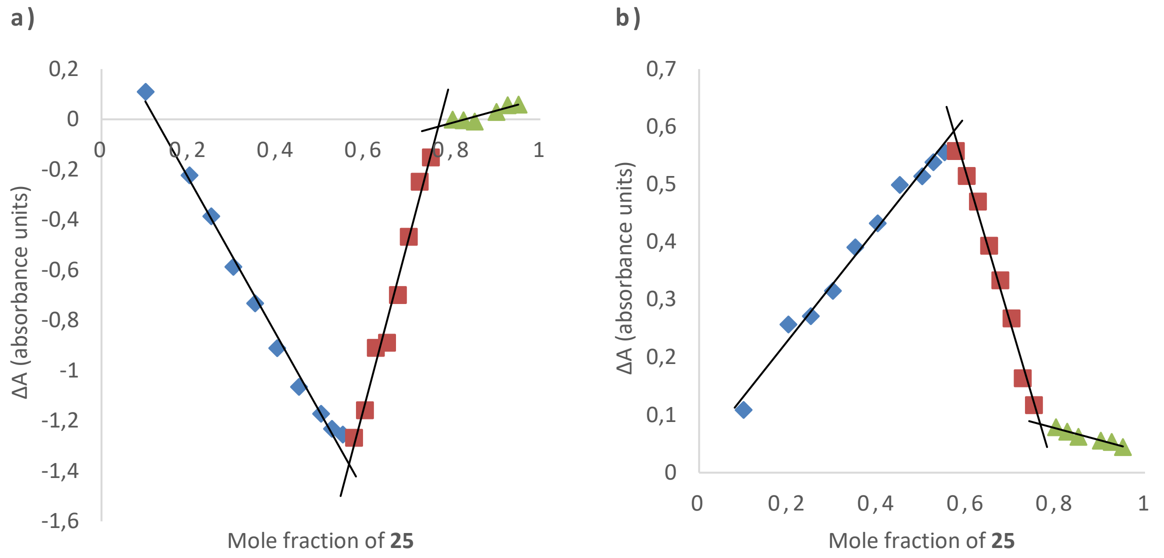

Figure S36. Job's plot of compound **25** in presence of Fe^{3+}: variation of the absorbance (ΔA) at the wavelength of 251 nm in **a)** or at 318 nm in **b)**, in ordinate, versus the mole fraction of **25**, in abscissa. X_1 (mole fraction that causes the maximum variation of absorbance) = 0.57; X_2 = 0.77; n_1 (number of ligand molecules per cation) = 1; n_2 = 3.

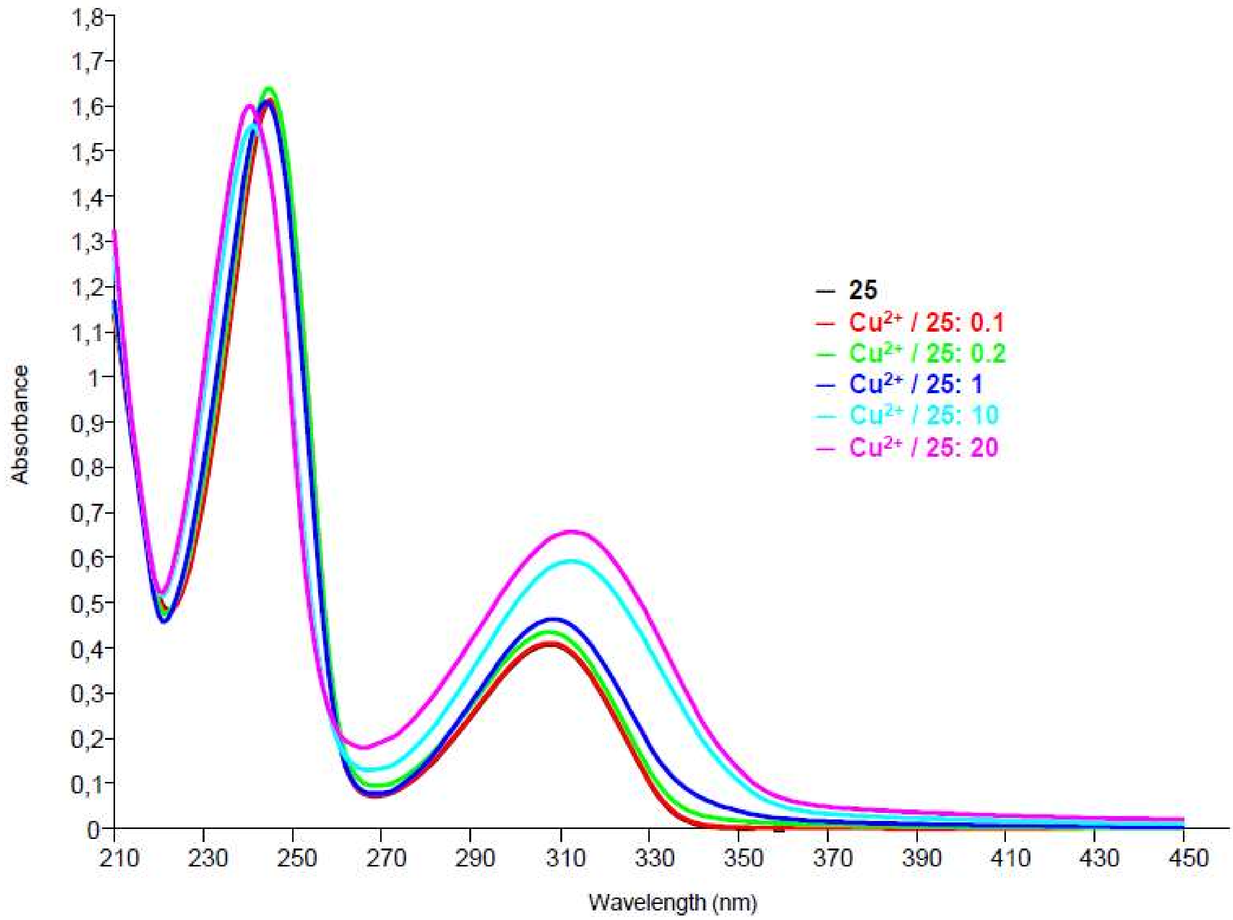

Figure S37. UV-Vis titration of ligand **25** with Cu^{2+}. The greatest variations in spectra with increasing amount of metal are observed at 270 and 330 nm, with an increase of absorbance.

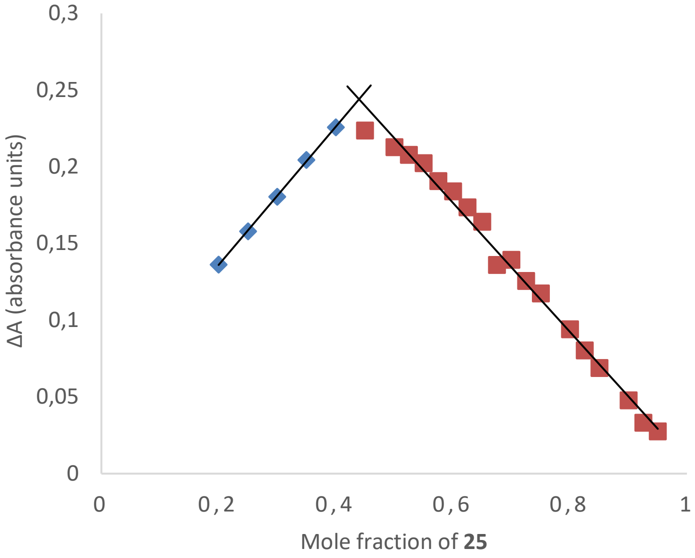

Figure S38. Job's plot of compound **25** in presence of Cu^{2+}: variation of the absorbance (ΔA) at the wavelength of 330 nm, in ordinate, versus the mole fraction of **25**, in abscissa. X (mole fraction that causes the maximum variation of absorbance) = 0.44; n (number of ligand molecules per cation) = 1.

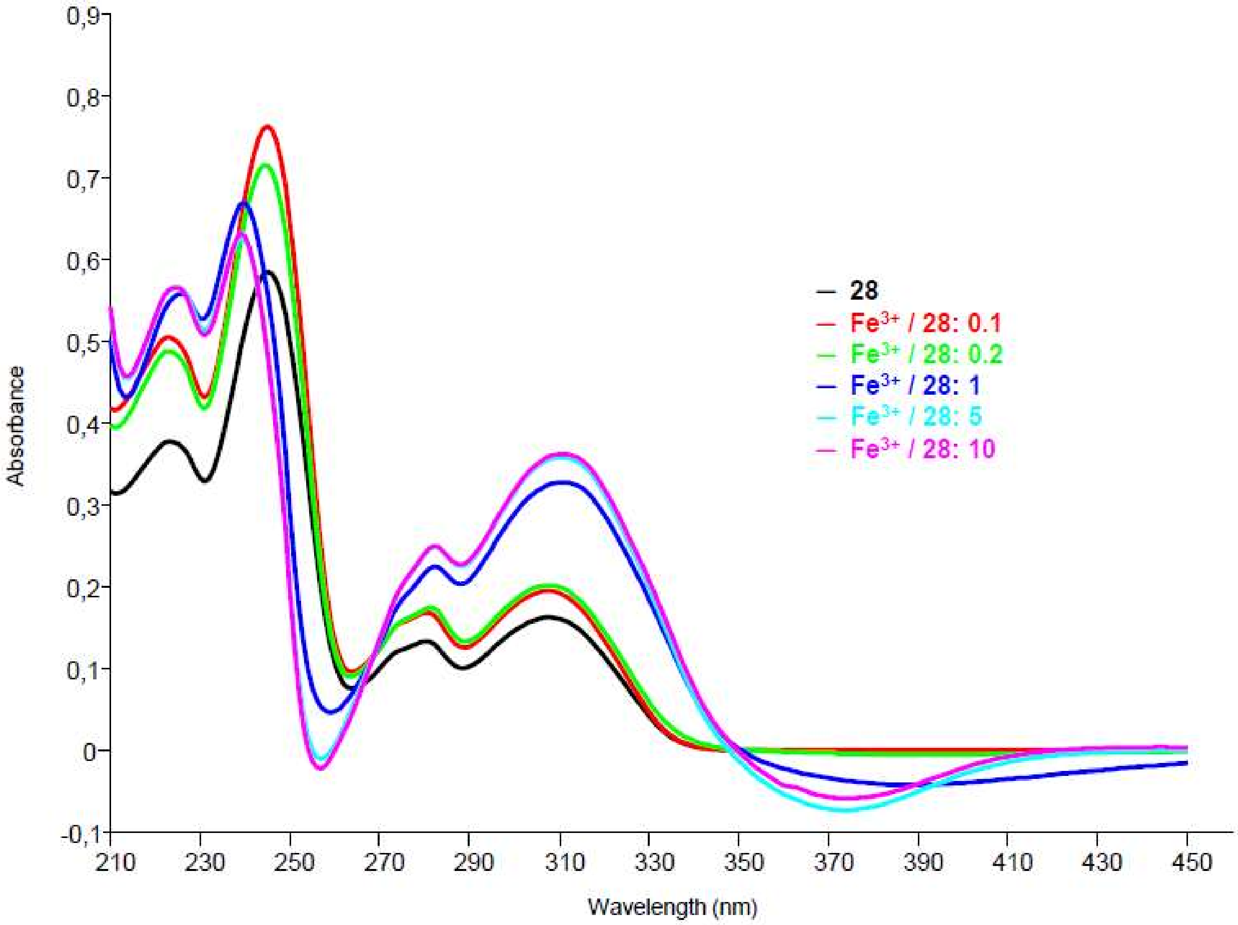

Figure S39. UV-Vis titration of ligand **28** with Fe^{3+}. The greatest variations in spectra with increasing amount of metal are observed at 222, 280 and 316 nm, with an increase of absorbance, and at 252 and 374 nm, with a reduction of absorbance. There are two isosbestic points at 267 and 348 nm.

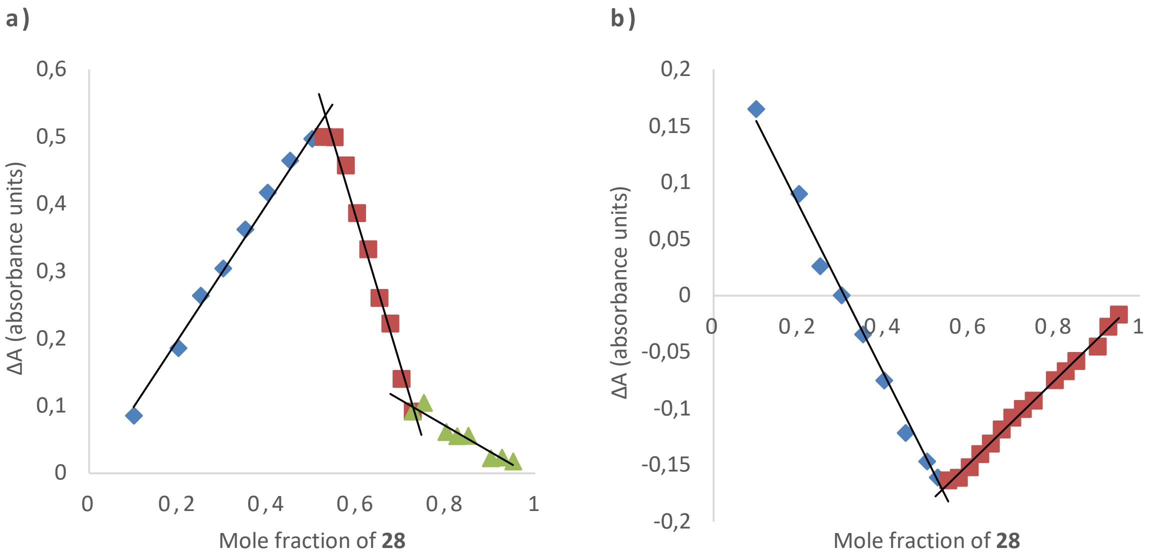

Figure S40. Job's plot of compound **28** in presence of Fe^{3+}: variation of the absorbance (ΔA) at the wavelength of 316 nm in **a)** or at 374 nm in **b)**, in ordinate, versus the mole fraction of **28**, in abscissa. X_1 (mole fraction that causes the maximum variation of absorbance) = 0.53; X_2 = 0.74; n_1 (number of ligand molecules per cation) = 1; n_2 = 3.

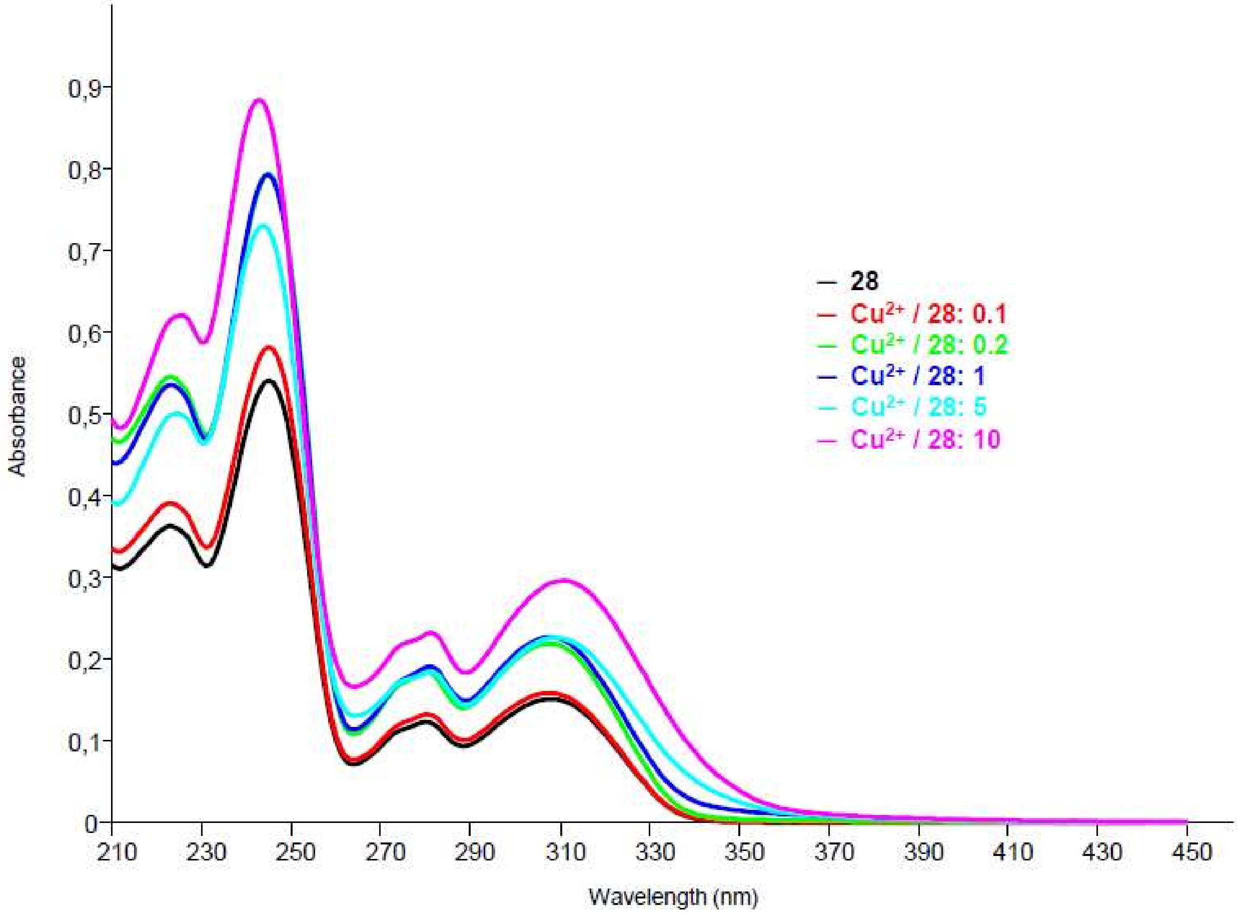

Figure S41. UV-Vis titration of ligand **28** with Cu^{2+}. The greatest variations in spectra with increasing amount of metal are observed at 222, 280 and 330 nm, with an increase of absorbance.

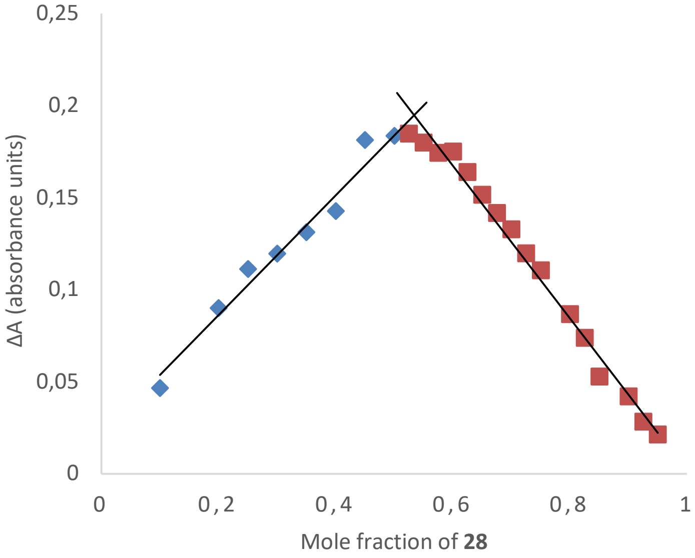

Figure S42. Job's plot of compound **28** in presence of Cu^{2+}: variation of the absorbance (ΔA) at the wavelength of 330 nm, in ordinate, versus the mole fraction of **28**, in abscissa. X (mole fraction that causes the maximum variation of absorbance) = 0.53; n (number of ligand molecules per cation) = 1.

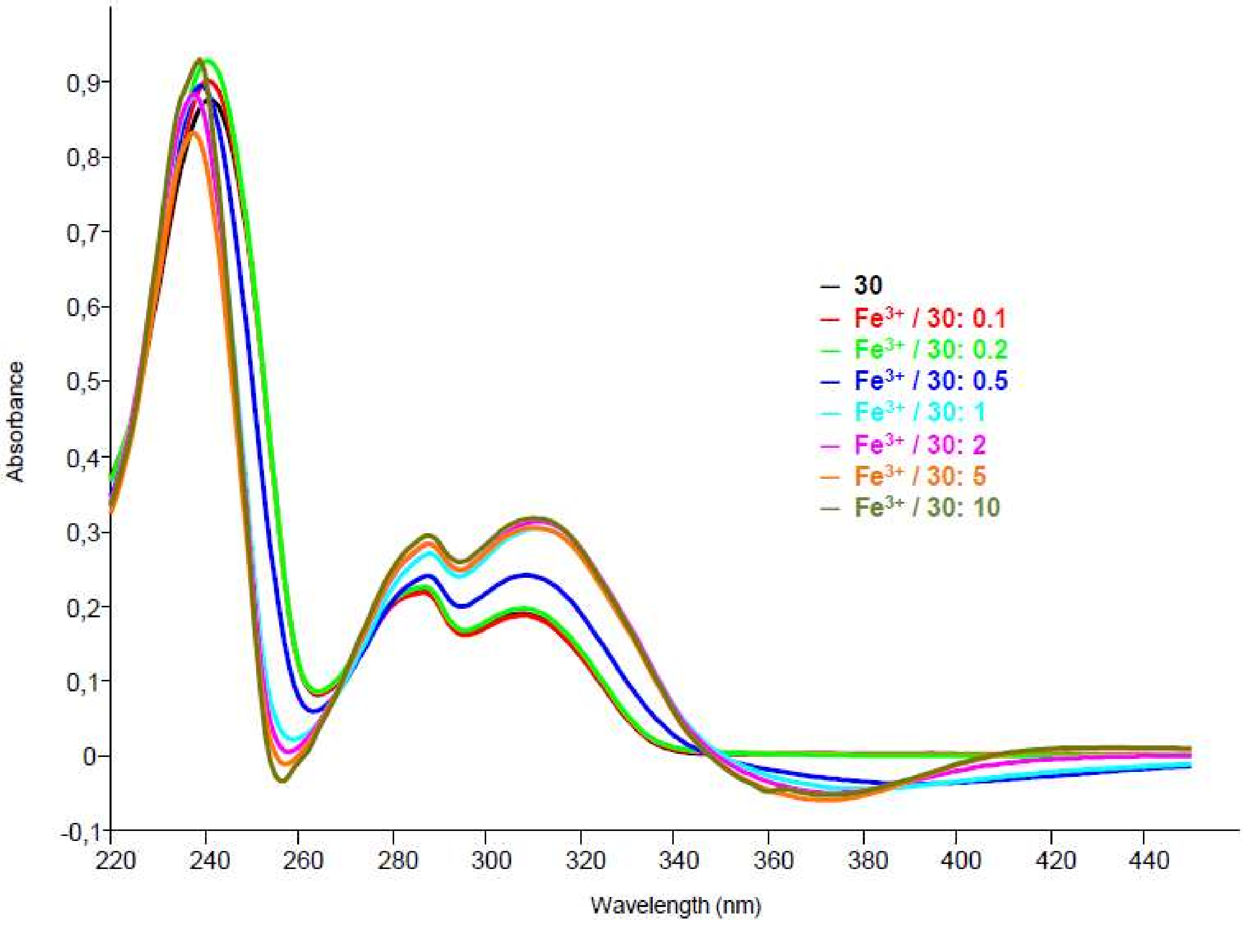

Figure S43. UV-Vis titration of ligand **30** with Fe^{3+}. The greatest variations in spectra with increasing amount of metal are observed at 256 and 372 nm, with a reduction of absorbance, and at 287 and 312 nm, with an increase of absorbance. There are two isosbestic points at 270 and 347 nm.

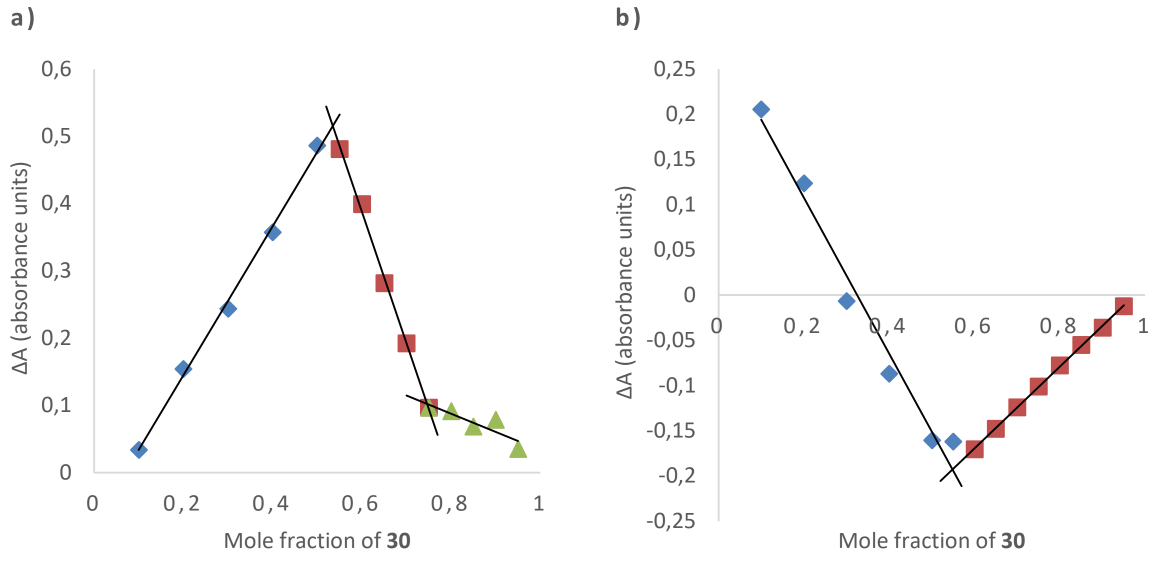

Figure S44. Job's plot of compound **30** in presence of Fe^{3+}: variation of the absorbance (ΔA) at the wavelength of 310 nm in **a)** or at 372 nm in **b)**, in ordinate, versus the mole fraction of **30**, in abscissa. X_1 (mole fraction that causes the maximum variation of absorbance) = 0.53 in **a)**, 0.55 in **b)**; X_2 = 0.74 in **a)**; n_1 (number of ligand molecules per cation) = 1; n_2 = 3.

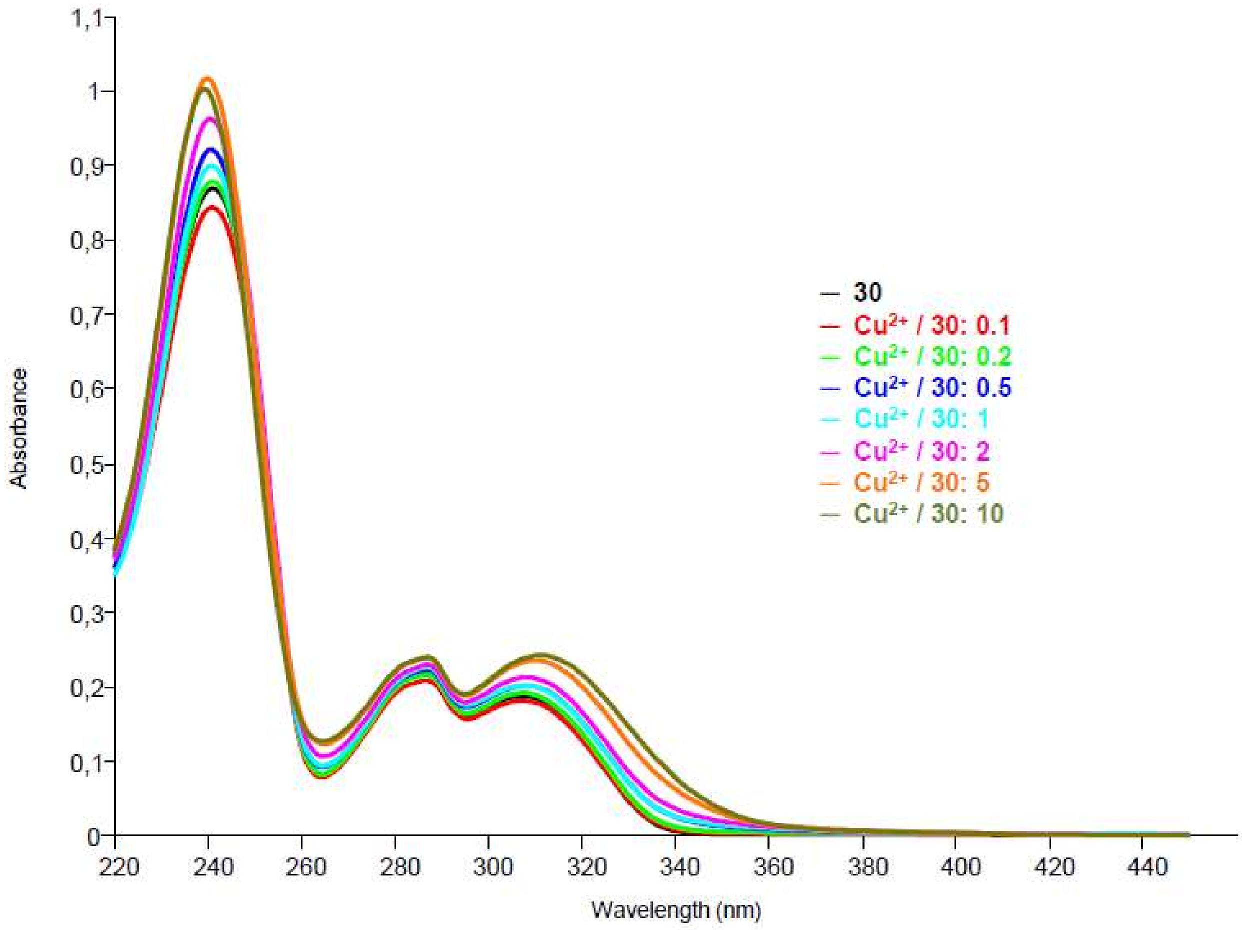

Figure S45. UV-Vis titration of ligand **30** with Cu^{2+}. The greatest variations in spectra with increasing amount of metal are observed at 265, 312 and 330 nm, with an increase of absorbance.

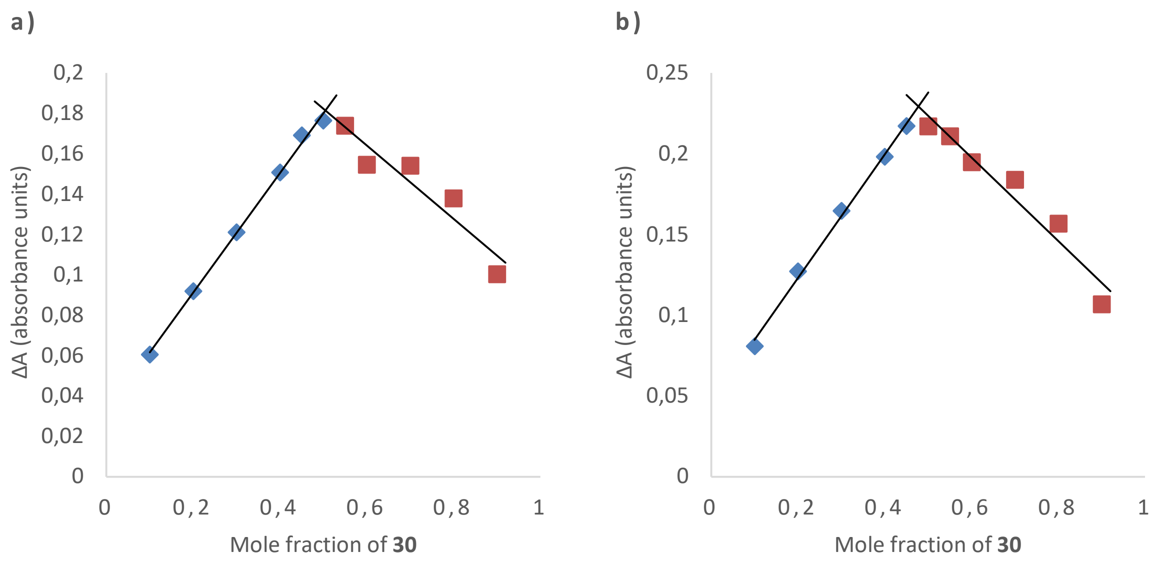

Figure S46. Job's plot of compound **30** in presence of Cu^{2+}: variation of the absorbance (ΔA) at the wavelength of 312 nm in **a)** or at 330 nm in **b)**, in ordinate, versus the mole fraction of **30**, in abscissa. X (mole fraction that causes the maximum variation of absorbance) = 0.50 in **a)**, 0.48 in **b)**; n (number of ligand molecules per cation) = 1.
